# Pt(PPh_3_)_4_ and Pt(PPh_3_)_4_@IL catalyzed hydroboration of ketones

**DOI:** 10.1038/s41598-023-47518-6

**Published:** 2023-11-19

**Authors:** Barbara Krupa, Jakub Szyling, Jędrzej Walkowiak

**Affiliations:** 1 Center for Advanced Technology, Adam Mickiewicz University, Uniwersytetu Poznanskiego 10, 61-614 Poznan, Poland; 2 Faculty of Chemistry, Adam Mickiewicz University, Uniwersytetu Poznanskiego 8, 61-614 Poznan, Poland

**Keywords:** Chemistry, Catalysis, Chemical synthesis

## Abstract

An efficient method for the reduction of various ketones via [Pt(PPh_3_)_4_]-catalyzed hydroboration with HBpin has been successfully developed for the first time. The protocol is suitable for symmetrical and unsymmetrical derivatives possessing electron donating or withdrawing functional groups. O-borylated products were easily converted to 2° alcohols via hydrolysis with high isolated yields. According to the low-temperature NMR spectroscopy, a reaction mechanism was proposed. Additionally, effective immobilization of the catalyst in the ionic liquid [BMIM][NTf_2_] was applied to increase the productivity of the process by carrying out reactions under the repetitive batch mode, obtaining higher TON values and limiting the amount of expensive Pt used. The catalyst stability and almost neglectable leaching were confirmed by ICP-MS analysis of the extracted mixture. A simple separation method via extraction with *n*-heptane, efficient catalyst immobilization, and the commercial availability of the Pt complex, make this protocol an attractive method for the hydroboration of ketones

## Introduction

Hydroboration is one of the most powerful methods for the incorporation of a boryl moiety into the structure of various compounds^1–3^. This reaction occurs by the addition of the B-H bond to compounds possessing unsaturated carbon–carbon or carbon-heteroatom bonds (e.g., C≡C^4^, C=C^1,5^, C=O^6^, C=N^7^). It is a versatile and straightforward synthetic tool that proceeds with high selectivity and excellent atom economy depending on the type of catalyst and reagents, as well as reaction conditions. Organoboron compounds obtained via the hydroboration process are characterized by high reactivity, low toxicity and possibilities for their further, simple functionalization in many catalytic and stoichiometric transformations, e.g., deborylation^8^, Suzuki–Miyaura cross-coupling^9^, cycloaddition^10^, alkenylation^11^ or propargylation^12^ making them important building blocks in the synthesis of fine chemicals^13,14^.

Hydroboration of carbonyl compounds is one of the most useful strategies for the synthesis of alcohols, obtained by the subsequent hydrolysis of boronate ether intermediates^15^. The reduction of aldehydes and ketones generates 1° and 2° alcohols respectively. These compounds are important scaffolds for the synthesis of biologically active molecules in the pharmaceutical industry^14,16^. Moreover, in contrast to conventional methods, which involve dangerous, highly reactive hydrides or flammable pressurized hydrogen gas, hydroboration often proceeds under mild conditions with 100% atomic economy which is in line with the principles of green chemistry. Moreover, the reduction might be carried out selectively, provided that the other unsaturated bonds are unreactive^6,15,17,18^.

Catalytic hydroboration of carbonyl derivatives can be performed in the presence of main group metals^19,20^, transition metals^3^, rare earth metal complexes^21^ and organocatalysts^22–25^. This subject had been comprehensively reviewed^6,8^. Transition metal-based catalysts are still very effective in hydroboration reactions including carbonyl compounds because of their ability to provide high reagent conversion, product yield and reaction selectivity. Their principle advantage over main group catalysts is low catalyst loading and the lack of any need to apply various additives or additional ligands^24,26–28^. These processes are mainly performed under homogeneous conditions, which on the one hand assure excellent selectivity towards the desired products and on the other, generate important economic and ecological issues, such as the lack of any possibility of recycling precious TM-catalysts or the generation of a vast amount of contaminated volatile organic solvents (VOSs). Thus, alternative protocols for the hydroboration of ketones following green chemistry rules and enhancing process sustainability should be developed. For the hydroboration of carbonyl compounds, reusable protocols based on nanoparticles^29,30^, metal−organic frameworks (MOF)^31–39^, metal-coordination polymers^40–42^ or silica-supported metal catalysts ^43,44^ have been reported. Very recently, Peters et al. reported the homogeneous cooperative Lewis acid-ammonium salt catalyzed hydroboration of ketones. The catalyst was recycled eleven times in the hydroboration reaction of acetophenone. For the first 9 runs, the product was obtained with a 99% yield, while in the last cycle, the conversion of ketone dropped to 75%. The accumulative TON value for this process was 2094^45^. An ionic liquid with the organic cation [BMIM]^+^ and metallic anion [FeCl_4_]^–^ has been used as an effective catalyst in the hydroboration of imines by Lin et al*.* The 15 repetitive batches with *N*-benzylideneaniline and pinacolborane were carried out with substrate conversion between 93 and 85%, giving an accumulative TON value of 449^46^.

**Figure 1:**
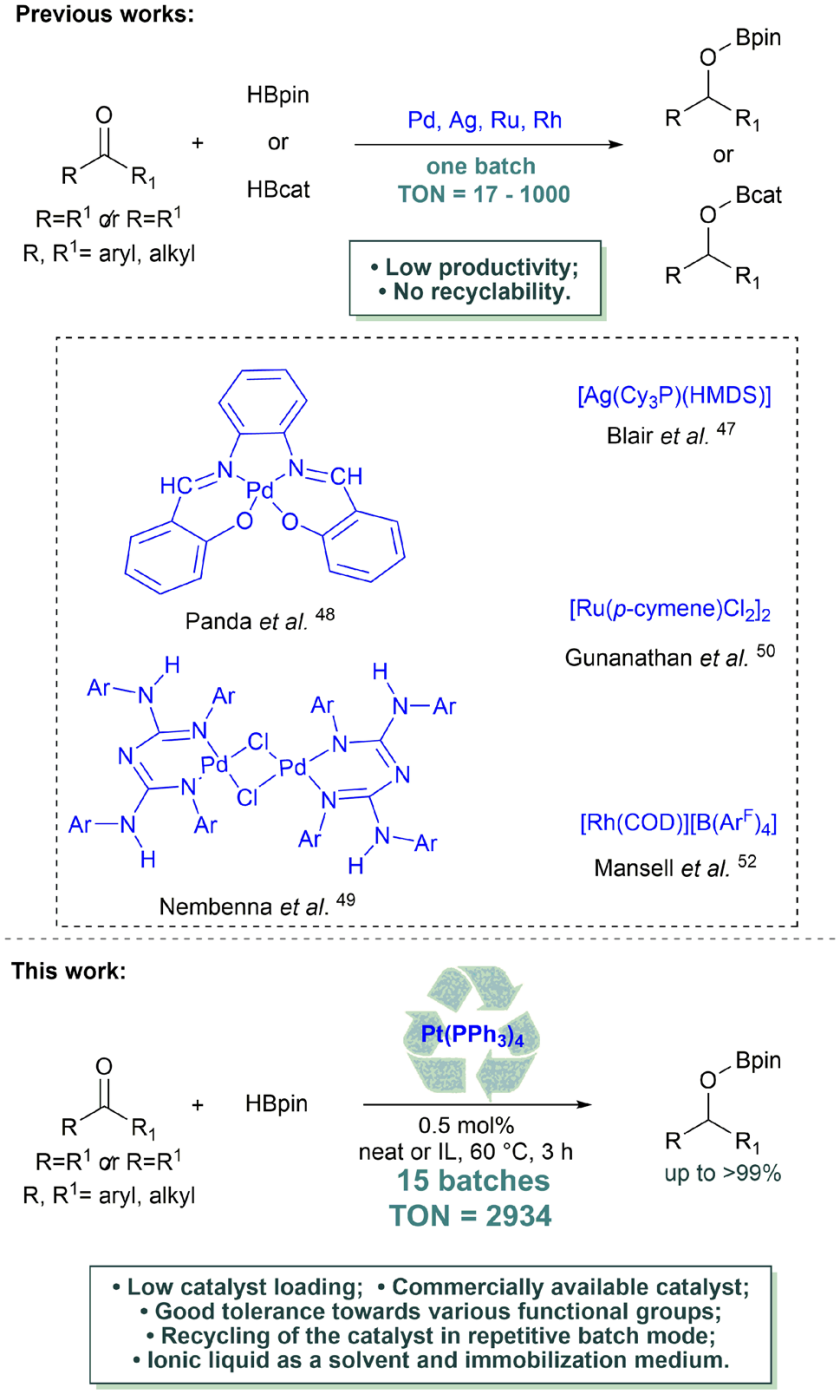
Hydroboration of ketones catalyzed by noble metal complexes.

TM-catalyzed hydroboration of C=O bonds is a powerful synthetic strategy leading to the corresponding alcohol; however, it has mainly been utilized for aldehydes^6^. In contrast to aldehydes, the efficient hydroboration of ketones in the presence of noble metal catalysts based on Ag^47^, Pd,^48,49^ Ru^50,51^ or Rh^52^ is limited to a few examples (Fig. 1). For the reaction catalyzed by silver, palladium and ruthenium compounds in solvent-free conditions, pinacolborane was used as the reducing agent. To achieve full reagents conversion, the reactions were carried out at 60 °C for 12‒15 h with 0.1‒6 mol% of the catalyst. For the rhodium complex, catecholborane was used as a boron source. The reaction was performed in deuterated benzene at room temperature for 30 or 60 min with 0.1 mol% of Rh catalyst. It should be pointed out that, hydroboration of ketones in the presence of precious metals has so far been conducted, in single batch mode, resulting in low productivity of the applied system. The TON (turnover numbers) values for the previously described protocols range from 17 for the palladium dimer complex to 1000 for the rhodium and ruthenium complexes (Fig. 1). Nevertheless, in each case an expensive and probably still active catalyst was sacrificed during the work-up procedure.

Platinum complexes have found application as effective catalysts in the hydroboration of alkenes^53–56^, alkynes^53^, allyl halides^57^ and allens^58^. Our group has already proved that platinum catalysts are effective promoters in hydrosilylation^59–66^, hydroboration and diboration reactions of unsaturated C≡C bonds^67^. However, the application of platinum complexes in the hydroboration of carbonyl compounds was unexplored until now.

The high activity of Pt catalysts in the functionalization of unsaturated C–C bonds and the lack of scientific reports concerning the application of Pt complexes in the hydroboration of ketones prompted us to explore this field. Herein, we present the results for the catalytic hydroboration of ketones in the presence of a commercially available and easily applied, stable platinum catalyst Pt(PPh_3_)_4_ followed by the efficient transformation of the obtained boronate ethers into secondary alcohols. Our main goal was to develop a simple and efficient protocol for the hydroboration of ketones in the presence of Pt catalysts, which could be easily applied in each organic laboratory. Having in mind green chemistry rules, additionally, we proposed an alternative protocol based on the application of the ionic liquid (IL), 1-butyl-3-methylimidazolium bis(trifluoromethanesulfonyl)imide ([BMIM][NTf_2_]), as a greener alternative for VOSs, which are typically used in ketones hydroboration. This approach also permitted catalyst immobilization and its recycling, intensifying the process according to its productivity and effectivity.

## Results and Discussion

In the first step of our study, a catalytic screening of various homogeneous and heterogeneous platinum catalysts (platinum black, PtO_2_, PtCl_2_, PtCl_2_(PPh_3_)_2_, Pt(η^2^-C_2_H_4_)(PPh_3_) and Pt(PPh_3_)_4_) was performed in the model reaction of acetophenone (**1a**) with pinacolborane (HBpin) (**2**). The process was carried out without the solvent in an inert atmosphere with 0.5 mol% of Pt-catalyst loading in 18 h at 60 °C. To exclude non-catalytic hydroboration of **1a** with **2** under these conditions, the control experiment without the catalyst was carried out. The borylated ether **3a** was formed with a poor 17% yield (Table 1, entry 1). The non-catalytic hydroboration of ketones is also feasible^68,69^. However, achieving high conversions requires harsh conditions: a high temperature (80 °C), a high excess of pinacolborane and a long reaction time (24 h)^69^. Additionally, for halogen-substituted acetophenones, these non-catalytic conditions were not sufficient to achieve full conversion of substrates^69^. The heterogeneous catalysts, such as platinum black and PtO_2_, showed moderate activity (Table 1, entries 2‒3). The hydroboration of **1** with **2a** in the presence of catalysts with the chlorine atom (PtCl_2_ and PtCl_2_(PPh_3_)_2_) also proceeded with the moderate conversion of **1a** however, a significant decrease in reaction selectivity was observed. For both systems, the undefined side-products **4** were detected (Table 1, entries 4–5). Pt(PPh_3_)_4_ and Pt(η^2^-C_2_H_4_)(PPh_3_)_2_ showed a great improvement in the conversion of acetophenone (**1a**). The full conversion and excellent selectivity towards **3a** were only observed for hydroboration of acetophenone (**1a**) with **2** in the presence of commercially available and air-stable Pt(PPh_3_)_4_ (Table 1, entry 7). For Pt(η^2^-C_2_H_4_)(PPh_3_)_2_, much lower selectivity was observed (Table 1, entry 6). In the next stage of process optimization, the influence of selected catalyst loading, substrates ratio, solvent type, time and temperature were verified (Table 1). The full conversion of **1a** was observed after 3 h with excellent product yield using 0.5 mol% of Pt(PPh_3_)_4_ (Table 1, entry 8). Shortening the time to 2 h resulted in the incomplete conversion of **1a** (Table 1, entry 9). Solvent choice had no significant influence on process efficiency. The reaction proceeded with quantitative yields in THF and toluene as well as without the solvent (Table 1, entries 10‒11). It should be noted that the Pt(PPh_3_)_4_ was dissolved very well in the substrates mixture, so having in mind green chemistry principles to reduce the amount of solvents, for the next experiments solvent-free conditions were applied. The reduction of catalyst loading from 0.5% to 0.25% provides a slightly lower conversion of **1a** (Table 1, entries 8 and 12). Similarly, lower yields of **3a** were observed when the reaction was performed at room temperature or 50 °C and using an equimolar ratio of reagents **1a** and **2** (Table 1, entries 13‒15). Changing the reducing agent from pinacolborane (**2**) to catecholborane (**2'**), resulted in similar acetophenone (**1**) conversions in both cases (Table 1, entry 16).

**Table 1:** Pt catalysts screening for hydroboration of acetophenone (**1a**) with pinacolborane (**2**).


Entry	Catalyst	Time (h)	Conversion of **1a** [%]^a^	Selectivity of **3a:4**[%]^a^
1	none	18	17	100:0
2	Platinum black	18	49	100:0
3	PtO_2_	18	45	100:0
4	PtCl_2_	18	47	0:100
5	PtCl_2_(PPh_3_)_2_	18	61	87:13
6	Pt(η^2^-C_2_H_4_) (PPh_3_)_2_	18	77	72:28
7	Pt(PPh_3_)_4_	18	> 99	100:0
8	Pt(PPh_3_)_4_	3	> 99	100:0
9	Pt(PPh_3_)_4_	2	96	100:0
10^b^	Pt(PPh_3_)_4_	3	99	100:0
11^c^	Pt(PPh_3_)_4_	3	99	100:0
12^d^	Pt(PPh_3_)_4_	3	97	100:0
13^e^	Pt(PPh_3_)_4_	3	41	100:0
14^f^	Pt(PPh_3_)_4_	3	94	100:0
15^g^	Pt(PPh_3_)_4_	3	95	100:0
16^h^	Pt(PPh_3_)_4_	3	99	100:0

Reaction conditions: acetophenone (**1a**) (0.5 mmol), pinacolborane (**2**) (0.6 mmol), 60 °C, argon atmosphere

^a^Yield was determined using GC–MS analysis

^b^Reaction in THF

^c^Reaction in toluene

^d^Catalyst loading 0.25 mol%

^e^Room temperature

^f^50 °C

^g^Acetophenone (**1a**) (0.5 mmol), pinacolborane (**2**) (0.5 mmol)

^h^Acetophenone (**1a**) (0.5 mmol), catecholborane (**2'**) (0.6 mmol).

**Figure 2:**
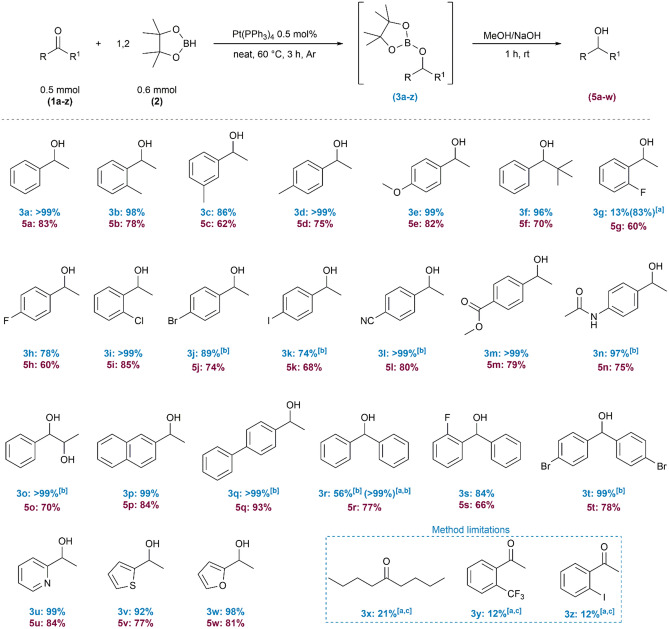
Substrate scope for hydroboration of ketones (**1a‒z**) catalyzed by Pt(PPh_3_)_4_. Yields were based on ^1^H NMR spectroscopy. (**a**) 18 h, (**b**) HBpin (2 mmol); (**c**) yield based on GC–MS analysis.

With the optimized reaction conditions in hand, the substrate scope was determined. Aromatic ketones with electron-donating groups (**1b**–**f**) were smoothly transformed into borylated ethers with high yields (> 86%). A slightly lower conversion was observed for 3-methylacetophenone (**1c**). The replacement of the methyl group with the sterically hindered *t*-Bu substituent had no significant influence on the conversion of ketone **1f**. The hydroboration of aromatic ketones with an electron-withdrawing group like nitriles (**1 l**) proceeded with quantitative yield. Lower reactivity (74‒89%) was observed for aryl ketones possessing halides in *para*-position **1 h**, **1j**, **1 k**. A significant decrease in product yield was observed for *o*-fluoroacetophenone (**1 g**). Extending the time to 18 h, gave the expected product **3 g** with very good yields. The hydroboration of another halogenated arylketone, 2-chloroacetophenone (**1i**), furnished product **3i** with quantitative yield. Pt(PPh_3_)_4_ was found to be the selective catalyst for the reduction of ketone, in the presence of nitrile (**3 l**), ester (**3 m**) and amide (**3n**) groups. The reactions proceeded with an excellent substrates conversion. To investigate the selectivity of hydroboration of carbonyl groups in both ketones and aldehydes, the equimolar ratio of acetophenone (**1a**), benzaldehyde, and pinacolborane (**2**) was used in the presence of a platinum catalyst. The experiment demonstrated that hydroboration of more reactive aldehyde is privileged (see Supplementary Information for details). In the case of diketones (**1o**), both C=O bonds were reduced to hydroxyl groups when 2 equiv. of pinacolborane were used. More bulky ketones with 2-naphtyl (**1p**) or 4-biphenyl (**1q**) substituents also provide products with 99% conversion of ketone and excellent selectivity for products **3p** and **3q** respectively. In the next step of our study, the substrate scope was extended to include diarylsubstituted ketones (**1r**‒**1t**) Hydroboration of benzophenone (**1r**) in the optimized reaction conditions gave product **3r** with moderate reaction yield; however, extending the reaction time to 18 h provided **3r** in almost quantitative yield. Keeping in mind the moderate reactivity of 2-fluorosubstituted acetophenone **1 g**, we were surprised to find that for analogue 2-fluorobenzophenone (**1 s**) the reaction proceeded in 3 h with a high (84%) yield of **3 s**. In turn, hydroboration of 4,4-dibromobenzophenone (**1t**) gave **3t** with excellent yield; however, twofold excess of **2** was needed. The catalytic method was effective for heteroaromatic compounds (**1u**–**1w**). Specifically, 2-acetylpyridine (**1u**) and 2-acetylfuran (**1w**) underwent hydroboration with nearly quantitative yields. However, for 2-acetylthiophene (**1v**), the reaction efficiency was slightly lower, reaching 92% yield. Notably, the developed catalytic system was less suitable for aliphatic compounds, which was exemplified by 5-nonanone (**1x**). Additionally, the reactions with aromatic substrates such as *o*-(trifluoromethyl)acetophenone and *o*-iodoacetophenone (**1y**-**z**) gave very low products yields. We attribute this limitation to the steric effects of the *ortho*-positioned groups on the phenyl ring in these substrates*.* All O-borylated ethers (**3**) were easily converted to the corresponding alcohols (**5**) via simple hydrolysis with 1 M NaOH solution in methanol. Moreover, it was possible to proceed hydrolysis in a one-pot procedure without isolation of borylated ether **3a**‒**w** intermediates. This approach allowed the process time to be shortened by the elimination of time-consuming separation steps of intermediates and reduction of the amount of volatile organic solvents used for purification (Fig. 2). Final products were purified by column chromatography using 9:1 hexane/ethyl acetate, 3:7 hexane/ethyl acetate (in case of **5o**) or 7:3 hexane/ethyl acetate in case of (**5n**), as an eluent. All obtained products **3** and **5** were characterized by nuclear magnetic resonance (^1^H NMR) and quantitative product extraction, the ionic liquid was dried under vacuum to remove any residual of the organic phase. The post-reaction mixture was characterized by GC–MS and ^1^H NMR analyses.

Generally, the hydroboration of ketones under solvent-free conditions at 60 ˚C with other noble metal catalysts (palladium^48,49^ and ruthenium^50^) has been described in the literature. However, by employing the platinum catalyst system, we were able to shorten the reaction time, and catalyst loading compared to the previously described systems.

**Figure 3:**
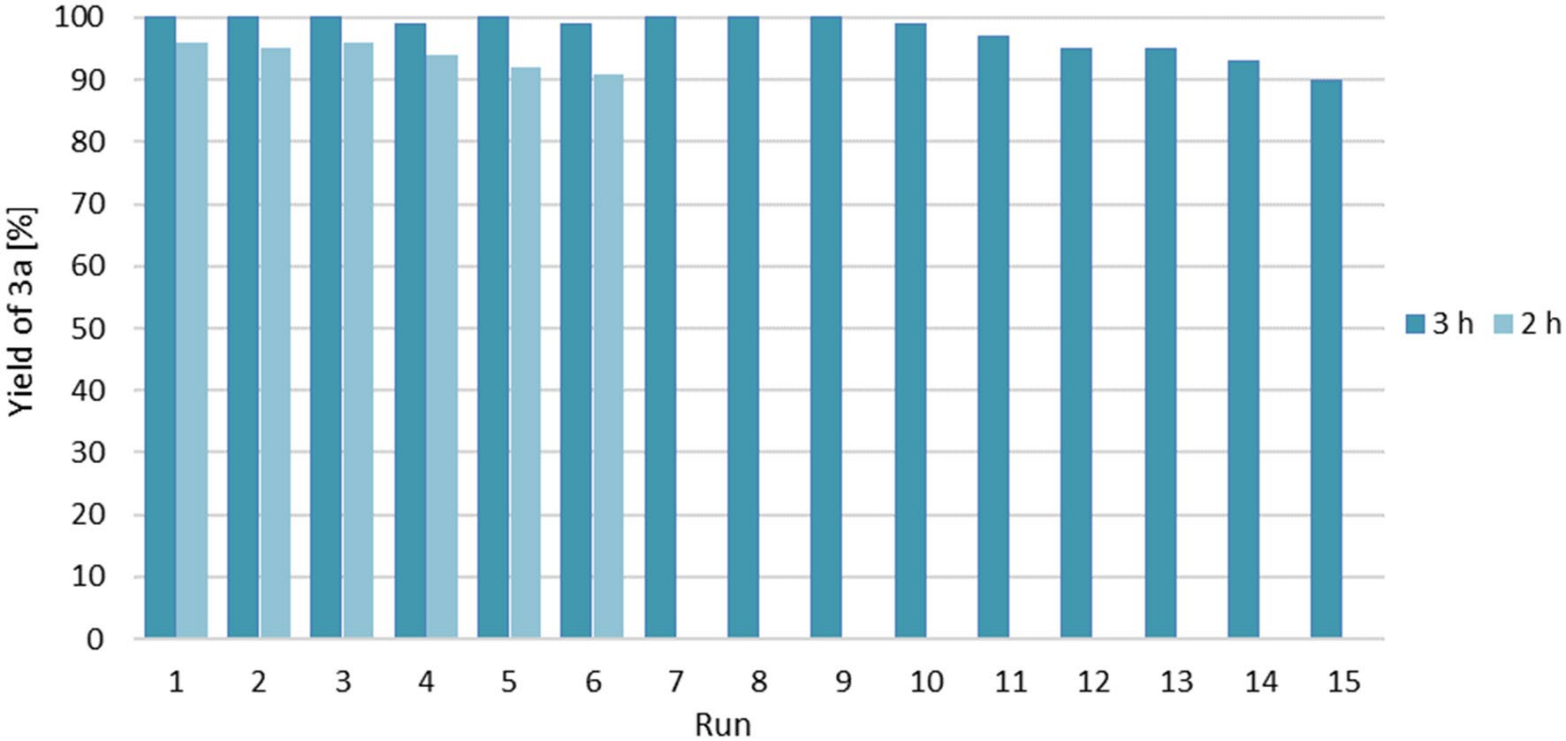
Yields of **3a** in the repetitive batch hydroboration of **1a** (0.5 mmol) with 2 (0.6 mmol) using 0.5 mol% Pt(PPh_3_)_4_@[BMIM][NTf_2_] at 60 °C in 2 and 3 h. Yields were determined by GC–MS and ^1^H NMR analyses.

In the next stage of our study, we verified the feasibility of the catalyst recycling by its immobilization in ILs. Based on our experience in the application of ILs in hydroboration and borylative coupling reactions^70–72^, we examined several ILs with melting points below room temperature (RTILs), such as 1-butyl-3-methylimidazolium bis(trifluoromethanesulfonyl)imide ([BMIM][NTf_2_], 1-butyl-1-methylpyrrolidinium bis(trifluoromethylsulfonyl)imide ([BMPyrr][NTf_2_]), 1-butyl-3-methylimidazolium tetrafluoroborate ([BMIM][BF_4_]) and 1-butyl-1-methylpyrrolidinium trifluoromethanesulfonate ([BMPyrr][OTf]), for catalyst immobilization. When the reaction of **1a** with **2** was carried out in the tested ionic liquids, without the addition of a platinum catalyst, no formation of reaction products was observed. The model reaction of acetophenone (**1a**) with pinacolborane (**2**), under standard reaction conditions (3 h at 60 °C, 0.5 mol% of Pt(PPh_3_)_4_) was used for the immobilization and catalyst recyclability test. Pt(PPh_3_)_4_ was immobilized in 150 mg of IL. A pale-yellow homogeneous solution was obtained after mixing the catalyst with the ionic liquid for 5 min. The reaction was carried out in a single-phase system since the reactants were highly soluble in the ionic liquid as well. After the reaction, the products were extracted with *n*-heptane. When the non-polar solvent was added, a two-phase system was formed. After quantitative product extraction, the ionic liquid was dried under vacuum to remove any residual of the organic phase. The post-reaction mixture was characterized by GC–MS and ^1^H NMR analyses. For [BMPyrr][NTf_2_], [BMPyrr][OTf], and [BMIM][BF_4_], we were able to observe poor conversion of substrate **1a** after 3 h. The best results were obtained by immobilization of the platinum catalyst in [BMIM][NTf_2_]. Almost quantitative (> 99%) formation of **3a** was observed in the first 10 cycles. Afterwards, an insignificant, gradual decrease in catalyst activity was noticed. To pinpoint the onset of catalyst deactivation, an experiment with a shorter reaction time of 2 h was conducted (Fig. 3). The catalyst's gradual deactivation became apparent around the 3^rd^ batch. This suggests that the initial reaction rate slightly decreases after each subsequent run, likely due to catalyst deactivation. It may be caused by introducing moisture or oxygen during the extraction process, and the formation of an inactive catalyst form (homogeneous or heterogeneous). However, we were unable to determine an inactive form of the catalyst. In total, 15 catalytic runs (3 h each) were conducted with the accumulative TON value 2934 (Fig. 3). Catalyst immobilization not only influences process effectivity but also allows for expensive catalyst reuse. It is especially important in the case of metals which have limited abundance. To determine the leaching of metal from the ionic liquid after the 1st, 2nd and 12th runs, ICP-MS analyses were carried out. The amount of platinum in the extracts was at an almost negligible level (not higher than 0.35 ppm), which proves sufficient catalyst immobilization and proper choice of the extractant.

**Figure 4:**
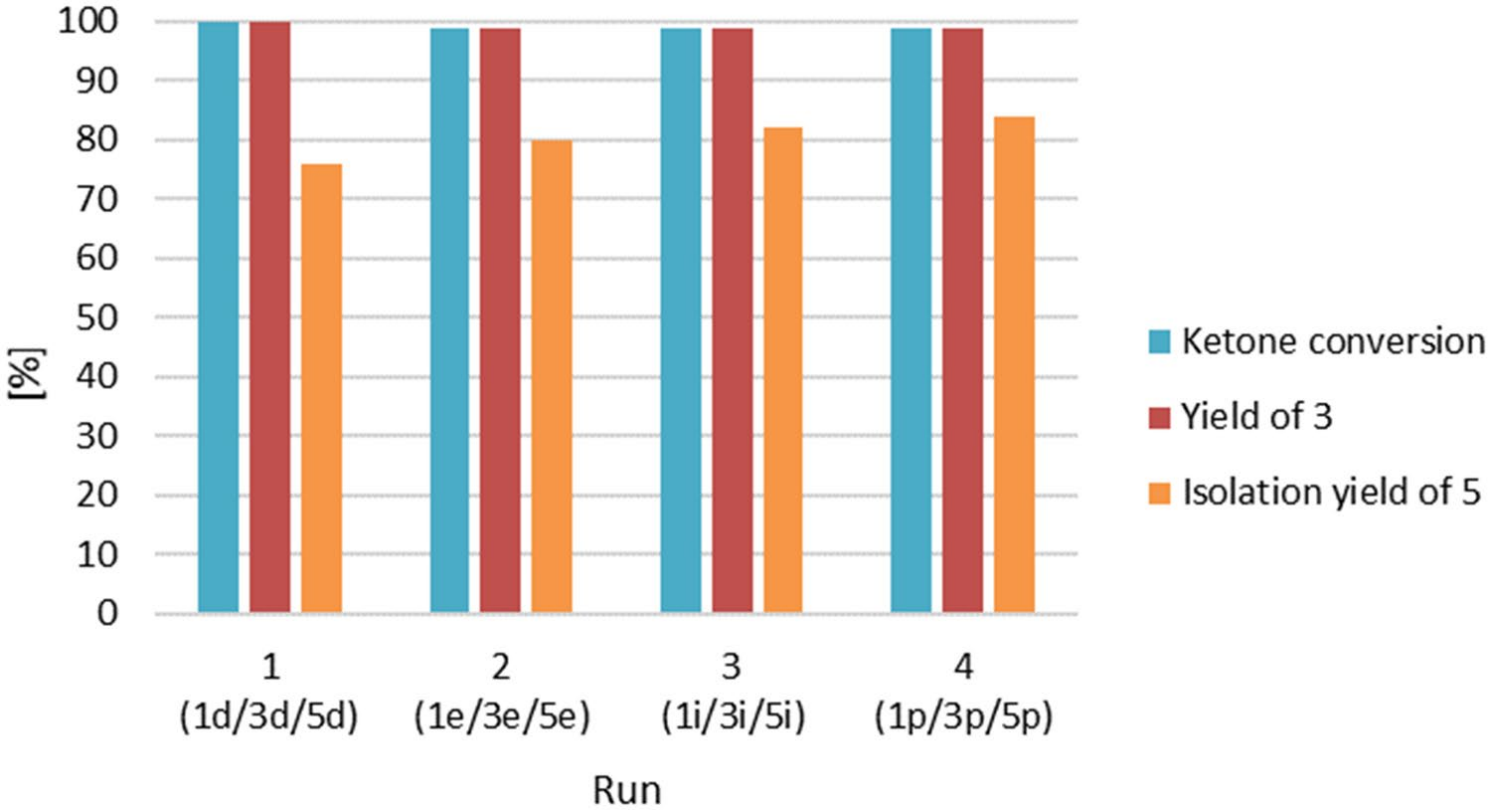
Hydroboration/extraction sequences performed in 0.5 mol% Pt(PPh_3_)_4_@[BMIM][NTf_2_] catalytic system for series of different aryl ketones (**1d-e**, **1i** and **1p**).

In the next stage of the study, to demonstrate the versatility of our protocol, we utilized the catalyst immobilized within an ionic liquid for a series of repetitive batches. Each reaction run involved the reduction of a different substrate. The same procedure that had been previously employed for hydroboration of model acetophenone in the presence of Pt(PPh_3_)_4_@[BMIM][NTf_2_] was applied. The reaction runs were executed sequentially with various ketones: *p*-methylacetophenone (**1d**) in the 1st run, *p*-methoxyacetophenone (**1e**) in the 2nd run, *o*-chloroacetophenone (**1i**) in the 3rd and 2-acetylnaphtalene (**3p**) in the 4th run. Nearly quantitative yields of the expected products (**3d**-**e**, **3i** and **3p**) were achieved (Fig. 4). After each run, the reaction mixture was separated from the catalytic system by *n*-heptane extraction and drying of the catalytic system under vacuum to remove any residual of the organic phase. It is worth noting that the extraction process was highly effective. The extracted solutions were devoid of reactants from previous cycles, which ^1^H NMR and GC–MS confirmed analyses.

**Figure 5:**
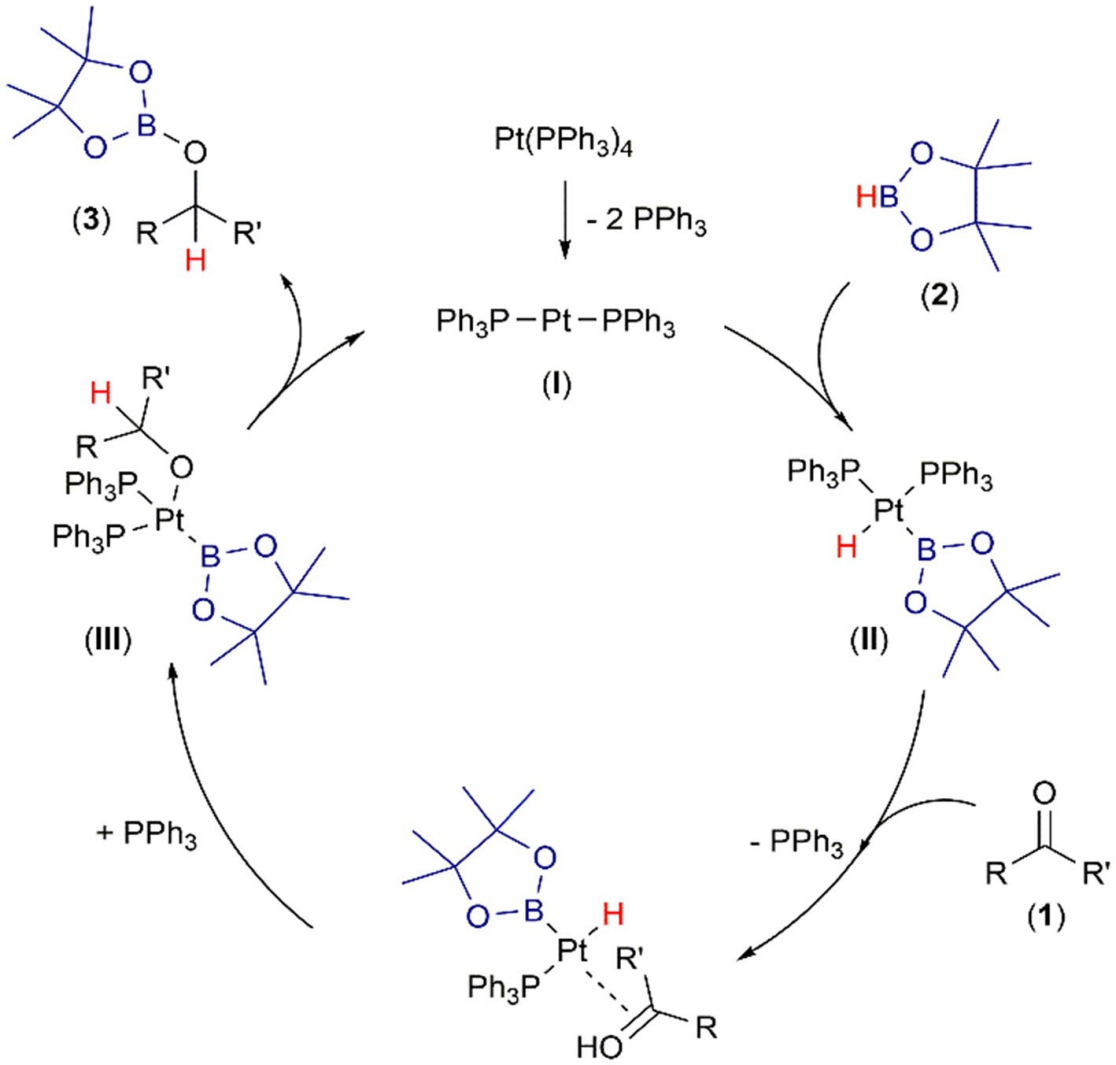
Proposed mechanism of Pt(PPh_3_)_4_-catalyzed hydroboration of ketones.

To better understand catalyst immobilization nature and reaction mechanism the composition of the catalytic system was investigated. After mixing the catalyst with the ionic liquid, and heating the mixture for 3 h in 60 °C, any new signals in the ^1^H and ^31^P NMR spectra were observed (See SI). This suggests that a new catalyst-ionic liquid adduct is not formed, and there is physical immobilization of Pt(PPh_3_)_4_ in [BMIM][NTf_2_]. Therefore, the reaction mechanism was investigated in a deuterated organic solvent without the involvement of IL (Fig. 5). The first step of the mechanism is believed to proceed via the dissociation of PPh_3_ (appearance of free PPh_3_ signal at − 6.3 ppm in ^31^P NMR spectrum) followed by oxidative addition of HBpin to Pt center. The low temperature ^1^H NMR spectrum of the reaction mixture contains a platinum-hydride, high-field shifted resonance at δ = − 16.31 ppm [^1^ J_(Pt–H)_ = 1215 Hz]^73^. The dissociation of another phosphine ligand and coordination of ketone followed by its insertion generates specie (specie III), which after reductive elimination gives O-borylated ether **3a** and regenerates the catalysts (disappearance of platinum-hydride signal) (See SI).

## Conclusion

In conclusion, a new catalytic method for the hydroboration of ketones was developed using a simple and commercially available Pt(PPh_3_)_4_ catalyst for the first time. It was found that 0.5 mol% of the catalyst is required to obtain excellent or very good conversion of various ketones possessing both electron-withdrawing and electron-donating functional groups such as methyl, methoxy, halides, nitrile, ester and amide. Moreover, the reaction time was shortened by up to 3 h in comparison to non-catalytic processes, which required higher temperatures and much longer reaction times. The obtained borylated ethers were easily transformed to the corresponding alcohols, which are important building blocks in organic and medicinal chemistry. Moreover, solvent-free conditions, as well as a one-pot procedure providing alcohols, without the isolation of the intermediates, are in line with the rules of green chemistry. The proposed reaction mechanism involves the oxidative addition of HBpin to the Pt center, followed by ketone coordination and reductive elimination of product (**3**) with the catalyst regeneration. Additionally, having in mind the costs of the catalyst as well as the limited abundance of platinum, the immobilization of Pt(PPh_3_)_4_ in ionic liquids was developed. Pt(PPh_3_)_4_@[BMIM][NTf_2_] allowed for effective catalyst recycling (up to 15 cycles with TON 2934) without a significant drop in catalyst activity and stability. Such strategy has a positive impact on process economy and efficiency.

## Methods

### Catalytic hydroboration of ketones with subsequent deborylation process – one pot procedure

A Schlenk vessel containing a magnetic stirring bar was charged with Pt(PPh_3_)_4_ (0.5 mol%, 3.22 mg). Then, the vessel was evacuated under vacuum and refilled with argon several times. Subsequently, pinacolborane (**2**) (1.2 eq., 0.6 mmol, 90 μL) or catecholborane (**2’**) (1.2 eq., 0.6 mmol, 72 mg) and ketone (**1a‒z**) (1 eq., 0.5 mmol) were added. The reaction was carried out for 3 h at 60 °C. After this time, the crude reaction mixture was analysed by ^1^H NMR and GC–MS analyses. Afterwards, the reaction mixture was quenched with 1 M solution of NaOH in methanol (1 mL) and allowed to stir for 1 h. Then methanol was evaporated, and 5 mL of water was added. The product was extracted with diethyl ether (3 × 5 mL). The combined organic layers were dried over anhydrous Na_2_SO_4_ and purified by column chromatography using 9:1 hexane/ethyl acetate (for product **5o** 3:7 hexane/ethyl acetate and for product **5n** 7:3 hexane/ethyl acetate) as the eluent.

### Hydroboration in a Pt(PPh_3_)_4_@[BMIM][NTf_2_] system under repetitive batch mode

Dry [BMIM][NTf_2_] (0.38 mol, 150 mg) and Pt(PPh_3_)_4_ (0.5 mol%, 3.22 mg) were added to a Schlenk vessel equipped with a magnetic stirring bar and vigorously stirred for 5 min. Then, pinacolborane (**2**) (0.6 mmol, 77 mg, 90 μL) and ketone (0.5 mmol) were added to the bright yellow Pt(PPh_3_)_4_@[BMIM][NTf_2_] solution. After 3 h, the products were extracted with dry *n*-heptane (4 × 1 mL) and analysed by GC–MS and ^1^H NMR analyses. The solvent residues were evaporated under vacuum. The Pt(PPh_3_)_4_@[BMIM][NTf_2_] catalytic system was applied to the next batches according to the procedure described above.

### Procedure of mechanistic studies

The Young’s NMR tube was charged under the argon with Pt(PPh_3_)_4_ (0.0321 mmol, 40 mg) and approx. 0.6 mL of dry CDCl_3_ was added. Subsequently, the 1.2 equiv. of HBpin (**2**) (0.0385 mmol, 4.9 mg) was added. The ^1^H and ^31^P NMR spectra were recorded at the low temperature (− 40 °C). Afterward, to the Young’s NMR tube the 1 equiv. of acetophenone (**1a**) (0.0321 mmol, 3.9 mg) was added under the argon and the reaction mixture was stirred over 18 h at 55 °C. After that, the low-temperature (− 40 °C) ^1^H NMR was performed.

### Supplementary Information

Supplementary Information.

## Data Availability

All data generated or analyzed during this study are included in this published article and its supplementary information file.
