# Supplementary material for: Pt(PPh3)4 and Pt(PPh3)4@IL catalyzed hydroboration of ketones
Source: Sci Rep. 2023 Nov 19;13:20237. doi: 10.1038/s41598-023-47518-6 (PMC10658173; doi:10.1038/s41598-023-47518-6)
Supplement: Supplementary file 1 — Supplementary Information. [file 41598_2023_47518_MOESM1_ESM.pdf]

Supporting Information

$Pt(PPh_3)_4$ and $Pt(PPh_3)_4$@IL catalyzed hydroboration of ketones

Barbara Krupa[a, b], Jakub Szyling[a] and Jędrzej Walkowiak[a]*

[a]Center for Advanced Technology, Adam Mickiewicz University, Uniwersytetu Poznanskiego 10, 61-614 Poznan, Poland.

[b]Faculty of Chemistry, Adam Mickiewicz University, Uniwersytetu Poznanskiego 8, 61-614 Poznan, Poland.

*E-mail: jedrzej.walkowiak@amu.edu.pl

Outline

1. General information S2

1.1. Materials S2

1.2 Methods S2

1.2.1. Nuclear magnetic resonance S2

1.2.2. Gas chromatography–mass spectrometry S3

1.2.3. Inductively coupled plasma mass spectrometry S3

1.2.4 Elemental analysis S3

2. Chemoselectivity test of $Pt(PPh_3)_4$ in hydroboration of ketones and aldehydes S3

3. Product yields in the hydroboration of ketones with pinacolborane (2) under repetitive batch mode S3

4. Products characterization S5

5. NMR spectra of products S20

6. Immobilization of $Pt(PPh_3)_4$ in [BMIM][NTf$_2$] S50

7. Stability of HBpin in [BMIM][NTf$_2$] S51

8. Mechanistic studies S53

9. References S55

1. **General information**

1.1. Materials

Acetophenone, (≥98.5%, Carlo Erba), 1-(*o*-tolyl)ethanone (98%, AmBeed), 3-methylacetophenone (97%, Alfa Aesar), 1-(*p*-tolyl)ethanone (97%, AmBeed), 2,2,2-trimethylacetophenone, (97%, Alfa Aesar), 4-methoxyacetophenone (98%, AmBeed), 2′-fluoroacetophenone (98%, AmBeed), 1-(4-flurophenyl)ethanone (98%, AmBeed), 1-(2-chlorophenyl)ethanone (95%, AmBeed), 4-iodoacetophenone (98%, Sigma-Aldrich), 4-acetylbenzonitrile (97%, AmBeed), benzophenone (≥99%, Acros-Organics), 2-fluorobenzophenone (98%, AmBeed), 4,4'-dibromobenzophenone (98%, abcr), 4-acetylbiphenyl (97%, AmBeed), 2-acetylnaphtalene (99%, abcr), methyl 4-acetylbenzoate (99.91%, AmBeed) *N*-(4-acetylphenyl)acetamide (98%, Fluorochem), 1-phenylpropane-1 2-diol (98%, Angene), 5-nonanone (Acros Organics, 98%), 2'-(trifluoromethyl)acetophenone (AmBeed, 98%), 2'-iodoacetophenone(Acros Organics, >99%), 2-acetylpyridine (AmBeed, 98%), 2-acetylfuran (Thermo Scientific, 99%), 2-acetylthiophene (AmBeed, 98%), catecholborane (98%, Sigma-Aldrich), benzaldehyde (Alfa Aesar, >99%), 4,4,5,5-tetramethyl-1,3,2-dioxaborolane (97%, Apollo Organics), ethylenebis(triphenylphosphine)platinum(0) (98%, AcrosOrganics), *cis*-dichlorobis(triphenylphosphine)platinum(II) (98%, Angene), platinum(II) chloride (99.9%, abcr), platinum(IV) oxide (Pt, 79-83%, Sigma-Aldrich), platinum black (≥99.95%, Sigma-Aldrich), 1-butyl-3-methylimidazolium bis(trifluoromethanesulfonyl)imide (≥ 97%, Sigma-Aldrich), 1-butyl-1-methylpyrrolidinium bis(trifluoromethylsulfonyl)imide (99%, Io-li-tec)), 1-butyl-3-methylimidazolium tetrafluoroborate (≥98%, Sigma-Aldrich), 1-butyl-1-methylpyrrolidinium trifluoromethanesulfonate (97%, Sigma-Aldrich), silica gel (pore size 60Å, 63-200 µm particle size, Sigma-Aldrich), sodium hydroxide (98.8%, Avantor Performance Materials Poland), sodium sulfate, anhydrous (>99.9%, Eurochem), diethyl ether (99.5%, Avantor Performance Materials Poland), ethyl acetate (99%, Avantor Performance Materials Poland), methanol (≥99.9%, Stanlab), *n*-heptane (>99%, Sigma-Aldrich), *n*-pentane (99%, Lach-Ner), (chloroform-d1 (99.8%, Deutero GmbH), dichloromethane-d2 (99.6%, Deutero GmbH). All chemicals were used without further purification and purchased from commercial sources, except acetophenone, which was purified by vacuum distillation and *n*-pentane and *n*-heptane were dried over NaH and distilled. Ionic liquids were dried overnight under vacuum at 60 °C before use. Tetrakis(triphenylphosphine)platinum(0) was synthesized according to the previously described procedure[1].

1.2 Methods

1.2.1. Nuclear magnetic resonance

The ^{1}H NMR and ^{13}C NMR spectra were recorded on a Bruker Ultrashield 300 MHz or Bruker Ascend 400 MHz spectrometers. The ^{11}B NMR spectra were received on Bruker Ascend 400 MHz spectrometer. Chemical shifts were reported in parts per million (ppm) with reference to the residual solvent peaks for ^{1}H and ^{13}C NMR or BF_3-Et_2O for ^{11}B NMR, respectively. The multiplicities are reported as follows: singlet (s), doublet (d), triplet (t), quartet (q), doublet of doublets (dd), multiplet (m), and broad resonances (br).

1.2.2. Gas chromatography–mass spectrometry

The mass spectra of the products were obtained by GC-MS analysis on a Bruker Scion 436-GC with a 30 m Varian DB-5 0.25 mm capillary column and a Scion SQ-MS mass spectrometry detector (temperature program: 60 °C (3 min), 10 °C/min, 250 °C (20 min).

1.2.3. Inductively coupled plasma mass spectrometry

In a 50 mL teflon beaker equipped with a stirrer, 150 mg of extract was placed and 2 mL of aqua regia was added. The mixture was heated for 1 h. The mixture was then diluted in demineralized water and filtered. Metal content was determined by inductively coupled plasma-mass spectroscopy (ICP-MS) with a NexION 350D (PerkinElmer).

1.2.4 Elemental analysis

Elemental analyzes were performed using a Vario EL III instrument.

2. Chemoselectivity test of $Pt(PPh_3)_4$ in hydroboration of ketones and aldehydes

A Schlenk vessel containing a magnetic stirring bar was charged with $Pt(PPh_3)_4$ (0.5 mol%, 3.22 mg). Then, the vessel was evacuated under vacuum and refilled with argon several times. Subsequently, pinacolborane (**2**) (1 eq., 0.5 mmol, 64 mg), acetophenone (**1a**) (1 eq., 0.5 mmol, 60 mg) and benzaldehyde (1 eq., 0.5 mmol, 53 mg) were added. The reaction was carried out for 3 hours at 60 °C. After this time, the crude reaction mixture was analyzed by ^1H NMR analysis.

3. Product yields in the hydroboration of ketones with pinacolborane (2) under repetitive batch mode

Table S1. Yields of **3a** and catalysts leaching for selected runs for the hydroboration of **1a** with **2** in the presence of $Pt(PPh_3)_4$@[BMIM][NTf_2] catalytic system (3 h, 60 °C).

Cycle number	Yield of **3a** (%)[a]	Catalyst leaching [ppm][b]
1	>99	0.35
2	>99	0.29
3	>99	
4	99	
5	>99	
6	99	
7	>99	
8	>99	
9	>99	
10	99	
11	97	
12	95	0.30
13	95	
14	93	
15	90	

[a] Yields were determined by GC-MS and ^1H NMR analyzes. [b] Determined by ICP-MS.

Table S2. Yields of **3a** obtained under repetitive batch hydroboration of **1a** with **2** in the presence of $Pt(PPh_3)_4$@[BMIM][NTf_2] catalytic system (2 h, 60 °C).

Cycle number	Conwersion of **1a** (%)[a]	Yield of **3a** (%)[a]
1	96	96
2	95	95
3	96	96
4	94	94
5	92	92
6	91	91

[a] Yields were determined by GC-MS and ^{1}H NMR analyzes.

Table S3. Yields of **3a** obtained under repetitive batch hydroboration of **1a** with **2** in the presence of $Pt(PPh_3)_4$@[BMIM][NTf_2] catalytic system (3 h, 60 °C).

Cycle number	Conwersion of **1** (%)[a]	Yield of **3** (%)[a]	Isolated yield of **5** (%)[a]
1 (**1d/3d/5d**)	100	100	76
2 (**1e/3e/5e**)	99	99	80
3 (**1i/3i/5i**)	99	99	82
4 (**1p/3p/5p**)	99	99	84

[a] Yields were determined by GC-MS and ^{1}H NMR analyzes.

4. Products characterization

*4,4,5,5-Tetramethyl-2-(1-phenylethoxy)-1,3,2-dioxaborolane (**3a**)*

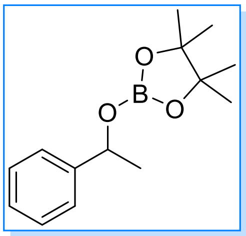

^{1}H NMR (300 MHz, $CDCl_3$, δ, ppm): 7.31 – 7.28 (m, 2H, Ph), 7.26 – 7.12 (m,3H, Ph), 5.17 (q, $J_{H\text{-}H}$ = 6.4 Hz, 1H, C<u>H</u>(CH_3)), 1.42 (d, $J_{H\text{-}H}$ = 6.5 Hz, 3H, CH(C<u>H</u>$_3$)), 1.16 (s, 6H, C(C<u>H</u>$_3$)$_2$), 1.13 (s, 6H, C(C<u>H</u>$_3$)$_2$). **MS (EI) [m/z (%)]**: 248(2), 233(27), 190(26), 129(17), 105(100), 77(21), 83(20), 80(16), 59(12), 55(8), 51(6). Characterized as a crude product. Analytical data are in agreement with the literature.[2,3]

*2-(1-Phenylethoxy)-1,3,2-benzodioxaborole (**3a'**)*

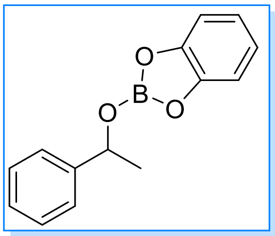

^{1}H NMR (400 MHz, $CDCl_3$, δ, ppm): 7.54 – 7.45 (m, 2H, Ph), 7.43 – 7.38 (m, 2H, Ph), 7.35 – 7.31 (m, 1H, Ph), 7.13 (m, 2H, Ph), 7.05 – 7.02 (m, 2H, Ph), 5.62 (q, $J_{H\text{-}H}$ = 6.5 Hz, 1H, C<u>H</u>(CH_3)), 1.70 (d, $J_{H\text{-}H}$ = 6.5 Hz, 3H, CH(C<u>H</u>$_3$)). **^{11}B NMR** (128 MHz, $CDCl_3$, δ, ppm): 23.20. Characterized as a crude product. Analytical data are in agreement with the literature.[4]

*4,4,5,5-Tetramethyl-2-(1-(o-tolyl)ethoxy)-1,3,2-dioxaborolane (**3b**)*

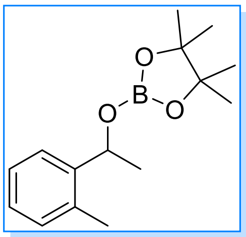

^{1}H NMR (300 MHz, $CDCl_3$, δ, ppm): 7.53 (d, $J_{H\text{-}H}$ = 8.6 Hz, 1H, Ph), 7.22 – 7.07 (m, 3H, Ph), 5.43 (q, $J_{H\text{-}H}$ = 6.4 Hz, 1H, C<u>H</u>(CH_3)), 2.34 (s, 3H, *o*-C<u>H</u>$_3$), 1.46 (d, $J_{H\text{-}H}$ = 6.4 Hz, 3H, CH(C<u>H</u>$_3$)), 1.23 (s, 6H, C(C<u>H</u>$_3$)$_2$), 1.20 (s, 6H, C(C<u>H</u>$_3$)$_2$). **MS (EI) [m/z (%)]**: 262(1), 247(26), 189(9), 136(8), 129(10), 121(33), 115(12), 116(24), 118(100), 119(62), 93(75), 91(73), 83(20), 77(47), 65(18), 57(13), 55(12). Characterized as a crude product. The analytical data are in agreement with the literature and were additionally supplemented with MS analysis.[5]

*4,4,5,5-Tetramethyl-2-(1-(m-tolyl)ethoxy)-1,3,2-dioxaborolane (**3c**)*

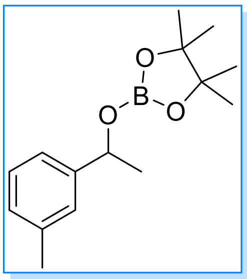

^{1}H NMR (400 MHz, $CDCl_3$, δ, ppm): 7.22 – 7.14 (m, 3H, Ph), 7.05 (d, J_{H-H} = 7.5 Hz, 1H, Ph), 5.22 (q, J_{H-H} = 6.4 Hz, 1H, C<u>H</u>(CH_3)), 2.34 (s, 3H, *m*-C<u>H</u>$_3$), 1.48 (d, J_{H-H} = 6.5 Hz, 3H, CH(C<u>H</u>$_3$)), 1.25 (s, 6H, C(C<u>H</u>$_3$)$_2$), 1.22 (s, 6H, C(C<u>H</u>$_3$)$_2$). **MS (EI) [m/z (%)]**: 262(13), 247(52), 204(28), 188(15), 129(22), 119(100), 117(23), 91(31), 83(19), 59(7). Characterized as a crude product. Analytical data are in agreement with the literature.[2,6]

*4,4,5,5-Tetramethyl-2-(1-(p-tolyl)ethoxy)-1,3,2-dioxaborolane (**3d**)*

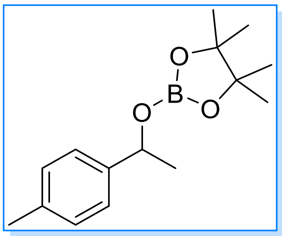

^{1}H NMR (400 MHz, $CDCl_3$, δ, ppm): 7.26 (d, J_{H-H} = 8.1 Hz, 2H, Ph), 7.13 (d, J_{H-H} = 7.9 Hz, 2H, Ph), 5.23 (q, J_{H-H} = 6.4 Hz, 1H, C<u>H</u>(CH_3)), 2.33 (s, 3H, *p*-C<u>H</u>$_3$), 1.48 (d, J_{H-H} = 6.4 Hz, 3H, CH(C<u>H</u>$_3$)), 1.25 (s, 6H, C(C<u>H</u>$_3$)$_2$), 1.22 (s, 6H, C(C<u>H</u>$_3$)$_2$). **MS (EI) [m/z (%)]**: 262(88), 261(17), 184(18), 183(100), 152(10), 115(6), 108(48), 107(25), 77(6), 51(13). Characterized as a crude product. Analytical data are in agreement with the literature.[2,3]

*2-(1-(4-Methoxyphenyl)ethoxy)-4,4,5,5-tetramethyl-1,3,2-dioxaborolan (**3e**)*

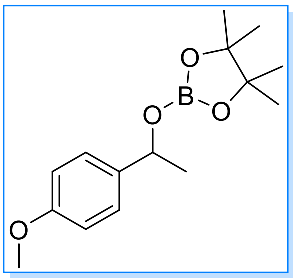

^{1}H NMR (400 MHz, $CDCl_3$, δ, ppm): 7.29 (d, J_{H-H} = 8.5 Hz, 2H, Ph), 6.85 (d, J_{H-H} = 8.8 Hz, 2H, Ph), 5.20 (q, J_{H-H} = 6.4 Hz, 1H, C<u>H</u>(CH_3)), 3.79 (s, 3H, *p*-OC<u>H</u>$_3$), 1.47 (d, J_{H-H} = 6.4 Hz, 3H, CH(C<u>H</u>$_3$)), 1.24 (s, 6H, C(C<u>H</u>$_3$)$_2$), 1.21 (s, 6H, C(C<u>H</u>$_3$)$_2$). **MS (EI) [m/z (%)]**: 278(4), 263(34), 181(8), 135(100), 129(13), 118(5), 105(10), 91(11), 83(7), 77(8), 59(4). Characterized as a crude product. Analytical data are data in agreement with a literature.[2,3]

*2-(2,2-Dimethyl-1-phenylpropoxy)-4,4,5,5-tetramethyl-1,3,2-dioxaborolane (**3f**)*

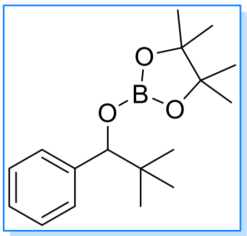

^{1}H NMR (400 MHz, $CDCl_3$, δ, ppm): 7.36 – 7.25 (m, 5H, Ph), 4.79 (s, 1H, C<u>H</u>(CH_3)), 1.23 (s, 6H, C(C<u>H</u>$_3$)$_2$), 1.17 (s, 6H, C(C<u>H</u>$_3$)$_2$), 0.93 (s, 9H, (C(C<u>H</u>$_3$)$_3$). **MS (EI) [m/z (%)]**: 290(4), 233(100), 232(24), 151(12), 129(12), 105(14), 91(20), 83(31), 55(11). Characterized as a crude product. The analytical data are in agreement with the literature.[7]

*2-(1-(2-Fluorophenyl)ethoxy)-4,4,5,5-tetramethyl-1,3,2-dioxaborolane (**3g**)*

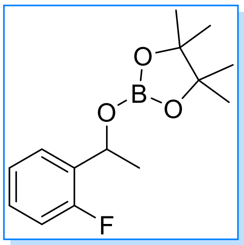

^{1}H MNR (300 MHz, $CDCl_3$, δ, ppm): 7.57 – 7.51 (m, 1H, Ph), 7.25 – 7.17 (m, 1H, Ph), 7.14 – 7.09 (m, 1H, Ph), 7.01 – 6.94 (m, 1H, Ph), 5.55 (q, $J_{H\text{-}H}$ = 6.4 Hz, 1H, C<u>H</u>(CH_3)), 1.49 (d, $J_{H\text{-}H}$ = 6.2 Hz, 3H, CH(C<u>H</u>$_3$)), 1.24 (s, 6H, C(C<u>H</u>$_3$)$_2$), 1.21 (s, 6H, C(C<u>H</u>$_3$)$_2$). **MS (EI) [m/z (%)]**: 266(4), 251(30), 208(25), 207(9), 165(6), 149(47), 129(25), 123(100), 103(28), 96(6), 83(17), 77(17), 59(16), 55(6). Characterized as a crude product. Analytical data are in agreement with the literature and were additionally supplemented with MS analysis.[8]

*2-(1-(4-Fluorophenyl)ethoxy)-4,4,5,5-tetramethyl-1,3,2-dioxaborolane (**3h**)*

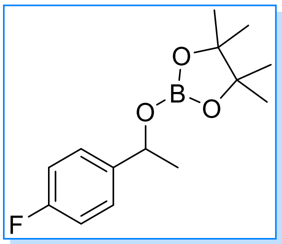

^{1}H NMR (400 MHz, $CDCl_3$, δ, ppm): 7.35 – 7.29 (m, 2H, Ph), 7.02 – 6.95 (m, 2H, Ph), 5.21 (q, $J_{H\text{-}H}$ = 6.4 Hz, 1H, C<u>H</u>(CH_3)), 1.46 (d, $J_{H\text{-}H}$ = 6.5 Hz, 3H, (CH(C<u>H</u>$_3$)), 1.23 (s, 6H, C(C<u>H</u>$_3$)$_2$), 1.20 (s, 6H, C(C<u>H</u>$_3$)$_2$). **MS (EI) [m/z (%)]**: 266(4), 251(17), 208(13), 193(10), 182(11), 151(5), 129(23), 123(100), 122(44), 103(23), 97(9), 83(24), 77(12), 69(5), 59 (11), 55(10). Characterized as a crude product. Analytical data are in agreement with the literature.[2,3]

*2-(1-(2-Chlorophenyl)ethoxy)-4,4,5,5-tetramethyl-1,3,2-dioxaborolane (**3i**)*

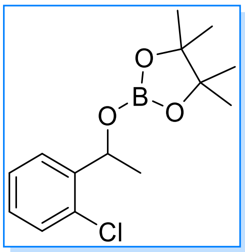

^{1}**H NMR** (400 MHz, CDCl$_3$, δ, ppm): 7.65 (dd, $J_{H\text{-}H}$ = 7.8, 1.6 Hz, 1H, Ph), 7.34 – 7.26 (m, 2H, Ph), 7.19 (m, 1H, Ph), 5.60 (q, $J_{H\text{-}H}$ = 6.3 Hz, 1H, C$\underline{H}$(CH$_3$)), 1.50 (d, $J_{H\text{-}H}$ = 6.3 Hz, 3H, CH(C$\underline{H}_3$)), 1.27 (s, 6H, C(C$\underline{H}_3$)$_2$), 1.24 (s, 6H, C(C$\underline{H}_3$)$_2$). **MS (EI) [m/z (%)]**: 283(4), 267(28), 247(30), 224(14), 189(46), 161(13), 147(11), 141(36), 139(100), 129(36), 103(54), 83(20), 77(32), 59(15), 55 (8). Characterized as a crude product. Analytical data are in agreement with the literature.[2,6]

*2-(1-(4-Bromophenyl)ethoxy)-4,4,5,5-tetramethyl-1,3,2-dioxaborolane (**3j**)*

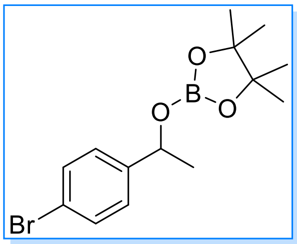

^{1}**H NMR** (400 MHz, CDCl$_3$, δ, ppm): 7.45 – 7.41 (m, 2H, Ph), 7.24 (d, $J_{H\text{-}H}$ = 8.3 Hz, 2H, Ph), 5.19 (q, $J_{H\text{-}H}$ = 6.4 Hz, 1H, C$\underline{H}$(CH$_3$)), 1.46 (d, $J_{H\text{-}H}$ = 6.5 Hz, 3H, CH(C$\underline{H}_3$)), 1.24 (s, 6H, C(C$\underline{H}_3$)$_2$), 1.21 (s, 6H, C(C$\underline{H}_3$)$_2$). **MS (EI) [m/z (%)]**: 327(15), 311(22), 268(18), 212(21), 202(40), 200(46), 185(100), 158(55), 147(32), 129(51), 120(13), 108(17), 99(17), 92(46), 82(10), 78(36), 70(30), 60(26). Characterized as a crude product. Analytical data are in agreement with the literature.[2,3]

*2-(1-(4–Iodophenyl)ethoxy)-4,4,5,5-tetramethyl-1,3,2-dioxaborolane (**3k**)*

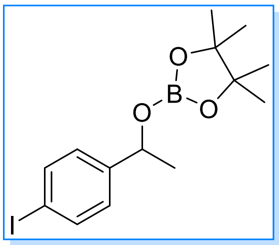

^{1}**H NMR** (400 MHz, CDCl$_3$, δ, ppm): 7.64 – 7.60 (m, 2H, Ph), 7.12 – 7.08 (m, 2H, Ph), 5.17 (q, $J_{H\text{-}H}$ = 6.4 Hz, 1H, C$\underline{H}$(CH$_3$)), 1.44 (d, $J_{H\text{-}H}$ = 6.5 Hz, 3H, CH(C$\underline{H}_3$)), 1.22 (s, 6H, C(C$\underline{H}_3$)$_2$), 1.20 (s, 6H, C(C$\underline{H}_3$)$_2$). **MS (EI) [m/z (%)]**: 374(5), 359(19), 248(21), 233(68), 231(93), 204(9), 189(21), 160(50), 147(100), 129(67), 121(14), 117(16), 102(36), 91(10), 85(24), 78(82), 77(58), 63(6), 59(70), 58(41), 50(31). Characterized as a crude product. The analytical data are in agreement with the literature and were additionally supported with MS analysis.[9]

*4-(1-((4,4,5,5-Tetramethyl-1,3,2-dioxaborolan-2-yl)oxy)ethyl)benzonitrile (**3l**)*

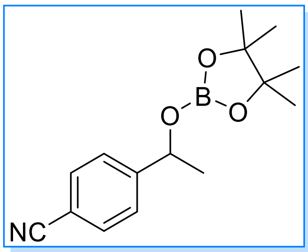

^{1}H NMR (300 MHz, $CDCl_3$, δ, ppm): 7.58 (d, $J_{H\text{-}H}$ = 8.3 Hz, 2H, Ph), 7.43 (d, $J_{H\text{-}H}$ = 8.2 Hz, 2H, Ph), 5.23 (q, $J_{H\text{-}H}$ = 6.4 Hz, 1H, $C\underline{H}(CH_3)$), 1.44 (d, $J_{H\text{-}H}$ = 6.5 Hz, 3H, $CH(C\underline{H}_3)$), 1.21 (s, 6H, $C(C\underline{H}_3)_2$), 1.18 (s, 6H, $C(C\underline{H}_3)_2$). **MS (EI) [m/z (%)]**: 273(13), 258(21), 215(100), 200(22), 173(31), 158(15), 131(42), 130(91), 129(53), 103(25), 85(25), 83(24), 77(12), 59(45), 55(13). Characterized as a crude product. The analytical data are in agreement with the literature and were additionally supplemented with MS analysis.[10]

*Methyl 4-(1-((4,4,5,5-tetramethyl-1,3,2-dioxaborolan-2-yl)oxy)ethyl)benzoate (**3m**)*

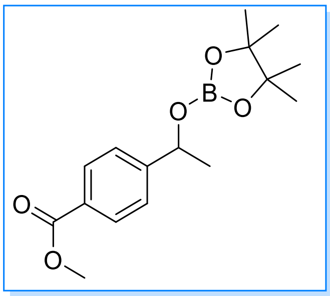

^{1}H NMR (300 MHz, $CDCl_3$, δ, ppm): δ 8.01 – 7.96 (m, 2H, Ph), 7.44 – 7.40 (m, 2H, Ph), 5.27 (q, $J_{H\text{-}H}$ = 6.5 Hz, 1H, $C\underline{H}(CH_3)$), 3.89 (s, 3H, $OC\underline{H}_3$), 1.48 (d, $J_{H\text{-}H}$ = 6.5 Hz, 3H, $CH(C\underline{H}_3)$), 1.23 (s, 6H, $C(C\underline{H}_3)_2$), 1.20 (s, 6H, $C(C\underline{H}_3)_2$). **MS (EI) [m/z (%)]**: 306(2), 291(37), 275(13), 263(19), 248(44), 223(12), 189(100), 175(13), 163(92), 147(21), 131(44), 129(44), 103(39), 91(20), 83(31), 78(19), 59(43). Characterized as a crude product. Analytical data are in agreement with the literature.[11]

*N-(4-(1-((4,4,5,5-tetramethyl-1,3,2-dioxaborolan-2-yl)oxy)ethyl)phenyl)acetamide (**3n**)*

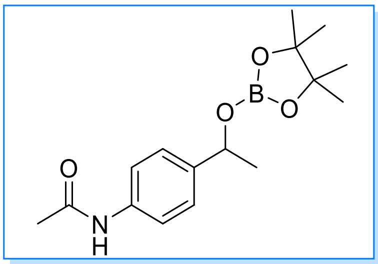

^{1}H NMR (300 MHz, $CDCl_3$, δ, ppm): 7.74 (s, 1H, $N\underline{H}$), 7.47 – 7.42 (m, 2H,Ph), 7.30 – 7.25 (m, 2H,Ph), 5.19 (q, J = 6.4 Hz, 1H, $C\underline{H}(CH_3)$), 2.13 (s, 3H, $C(C\underline{H}_3)$), 1.45 (d, J = 6.5 Hz, 3H, $CH(C\underline{H}_3)$, 1.23 (s, 6H, $C(C\underline{H}_3)_2$), 1.20 (s, 6H, $C(C\underline{H}_3)_2$). **^{13}C NMR** (75 MHz, $CDCl_3$, δ, ppm): 168.66(CO), 140.55 (Ph), 137.10(Ph), 126.08(Ph), 119.89 (Ph), 83.28 ($\underline{C}(CH_3)_2$),, 72.34, ($\underline{C}(CH_3)$), 24.67 $C(\underline{C}H_3)$), 24.64($C(\underline{C}H_3)_2$), 24.62 $CH(\underline{C}H_3)$) **^{11}B NMR** (128 MHz, $CDCl_3$, δ, ppm):. 22.31 **MS (EI) [m/z (%)]**: 305(10), 290(100), 248(17), 237(43), 215(51), 161(39), 137(82), 129(39), 119(81), 91(20), 77(90), 59(67). **Elem. Anal**. calcd (%) for

$C_{16}H_{24}BNO_4$: C, 62.97; H, 7.93; found C, 63.02; H, 7.99. Colorless liquid. Isolation yield 89% (150.7 mg).

*2,2'-((1-Phenylpropane-1,2-diyl)bis(oxy))bis(4,4,5,5-tetramethyl-1,3,2-dioxaborolane) (**3o**)*

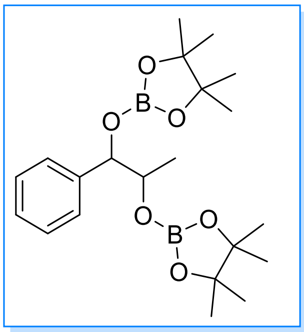

^{1}H NMR (300 MHz, $CDCl_3$, δ, ppm): 7.40 – 7.23 (m, 5H, Ph), 5.02 (d, J_{H-H} = 5.2 Hz, 0.82H, CHC<u>H</u>- *syn*), 4.87 (d, J_{H-H} = 7.4 Hz, 0.18H, CHC<u>H</u>-*anti*), 4.82 – 4.74 (m, 0.18H, CHC<u>H</u>-*anti*), 4.34 – 4.17 m, 0.82H, (CHC<u>H</u>-*syn*), 1.27 – 1.14 (m, 27H, CH(C<u>H</u>$_3$) overlap). **^{13}C NMR** (101 MHz, $CDCl_3$, δ, ppm): 140.26 (Ph), 127.99(Ph), 127.49(Ph), 127.03(Ph), 82.98(<u>C</u>(CH$_3$)$_2$), 82.64 (<u>C</u>(CH$_3$)$_2$),79.45 (CH<u>C</u>H), 74.27(<u>C</u>HCH), 24.75 (C(<u>C</u>H$_3$)$_2$), 24.69 (C(<u>C</u>H$_3$)$_2$), 24.63 (C(<u>C</u>H$_3$)$_2$), 24.56 (C(<u>C</u>H$_3$)$_2$), 17.01 (CH(<u>C</u>H$_3$)). **^{1}B NMR** (128 MHz, $CDCl_3$, δ, ppm): 22.20, 21.08. **MS (EI) [m/z (%)]**: 404(2), 346(3), 303(3), 289(4), 259(12), 241(12), 233(100), 185(13), 155(12), 129(5), 117(11), 82(8), 55(10), 43(9). **Elem. Anal**. calcd (%) for $C_{21}H_{34}B_2O_6$: C, 62.42; H, 8.48; found C, 62.29; H, 8.41. Colorless liquid. Isolation yield 90% (196.6 mg).

*4,4,5,5-Tetramethyl-2-(1-(naphthalen-2-yl)ethoxy)-1,3,2-dioxaborolane (**3p**)*

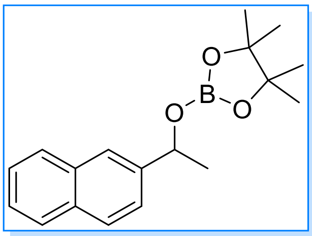

^{1}H NMR (400 MHz, $CDCl_3$, δ, ppm): 7.88 – 7.81 (m, 4H, napht), 7.54 – 7.43 (m, 3H, napht), 5.44 (q, J_{H-H} = 6.4 Hz, 1H, C<u>H</u>(CH$_3$)), 1.60 (d, J_{H-H} = 6.5 Hz, 3H, CH(C<u>H</u>$_3$)), 1.27 (s, 6H, C(C<u>H</u>$_3$)$_2$), 1.24 (s, 6H, C(C<u>H</u>$_3$)$_2$). **MS (EI) [m/z (%)]**: 298(15), 283(37), 240(9), 215(6), 155(100), 154(24), 153(28), 129(22), 115(7), 83(7), 59(6). Characterized as a crude product. The analytical data are in agreement with the literature and were additionally supplemented with MS analysis[2].

*2-(1-([1,1'-Biphenyl]-4-yl)ethoxy)-4,4,5,5-tetramethyl-1,3,2-dioxaborolane (**3q**)*

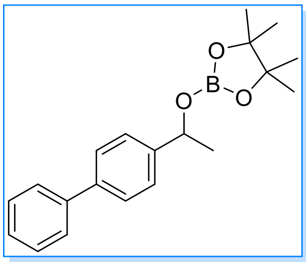

^{1}H NMR (300 MHz, $CDCl_3$, δ, ppm): 7.47 – 7.41 (m, 4H, Ph), 7.34 – 7.27 (m, 4H, Ph), 7.19 (m, 1H, Ph), 5.17 (q, $J_{H\text{-}H}$ = 6.4 Hz, 1H, C<u>H</u>(CH_3)), 1.41 (d, $J_{H\text{-}H}$ = 6.5 Hz, 3H, CH(C<u>H</u>$_3$)), 1.12 (s, 6H, C(C<u>H</u>$_3$)$_2$), 1.09 (s, 6H, C(C<u>H</u>$_3$)$_2$). **^{13}C NMR** (101 MHz, $CDCl_3$, δ, ppm): 143.7 (Ph), 141.0 (Ph), 140.1 (Ph), 128.8 (Ph), 127.2 (Ph), 127.1 (Ph), 127.0 (Ph), 125.8 (Ph), 82.8 (<u>C</u>(CH_3)$_2$), 72.4 (<u>C</u>H(CH_3)), 25.5 (C(<u>C</u>H$_3$)$_2$), 24.6 (CH(<u>C</u>H$_3$)). **^{11}B NMR** (128 MHz, $CDCl_3$, δ, ppm): 22.24. **MS (EI) [m/z (%)]**: 324(4), 309(8), 262(81), 261(18), 198(12), 183(100), 181(19), 155(17), 152(19), 129(9), 108(35), 107(22), 77(14), 51(17). **Elem. Anal**. calcd (%) for $C_{20}H_{25}BO_3$: C, 74.09; H, 7.77; found C, 76.16; H, 7.61. White solid. Isolation yield 99% (160.1 mg).The compound **3q** was synthesized and characterized for the first time.

*2-(Benzhydryloxy)-4,4,5,5-tetramethyl-1,3,2-dioxaborolane (**3r**)*

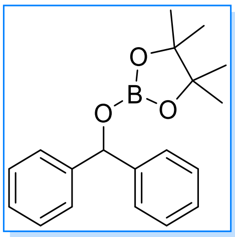

^{1}H NMR (400 MHz, $CDCl_3$, δ, ppm): 7.41 – 7.37 (m, 4H, Ph), 7.32 – 7.27 (m, 4H, Ph), 7.25 – 7.20 (m, 2H, Ph), 6.18 (s, 1H, C<u>H</u>(Ph)$_2$), 1.20 (s, 12H, C(C<u>H</u>$_3$)$_2$). **MS (EI) [m/z (%)]**: 310(3), 252(23), 227(35), 210(17), 183(15), 167(100), 165(45), 152(21), 105(7), 84(22), 77(6), 59(7). Characterized as a crude product. Analytical data are in agreement with the literature.[2,12]

*2-((2-Fluorophenyl)(phenyl)methoxy)-4,4,5,5-tetramethyl-1,3,2-dioxaborolane (**3s**)*

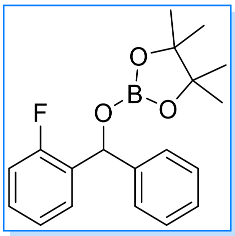

^{1}H NMR (400 MHz, $CDCl_3$, δ, ppm): 7.53 – 7.49 (m, 1H, Ph), 7.36 (dd, $J_{H\text{-}H}$ = 7.5, 1.7 Hz, 2H, Ph), 7.25 – 7.21 (m, 2H, Ph), 7.19 – 7.12 (m, 2H, Ph), 7.07 – 7.04 (m, 1H, Ph), 6.93 – 6.90 (m, 1H, Ph), 6.44 (s, 1H, C<u>H</u>(Ph)$_2$), 1.13 (s, 12H, C(C<u>H</u>$_3$)$_2$). **MS (EI) [m/z (%)]**: 328(4), 270(9), 245(18), 227(44), 201(9), 185(100), 183(38), 170(8), 165(50), 84(21), 59(9). Characterized as a crude product. The analytical data are in agreement with the literature and were additionally supplemented with MS analysis.[13]

*2-(Bis(4-bromophenyl)methoxy)-4,4,5,5-tetramethyl-1,3,2-dioxaborolane (**3t**)*

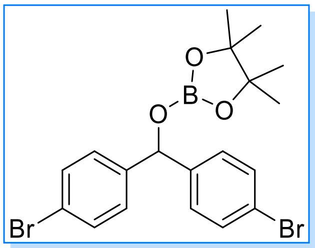

^{1}H NMR (300 MHz, $CDCl_3$, δ, ppm): 7.35 (d, $J_{H\text{-}H}$ = 8.4 Hz, 4H, Ph), 7.14 (d, $J_{H\text{-}H}$ = 8.4 Hz, 4H, Ph), 5.99 (s, 1H, CH(Ph)$_2$), 1.12 (s, 12H, C(CH$_3$)$_2$). **^{13}C NMR** (101 MHz, $CDCl_3$, δ, ppm): 141.7 (C(COH)), 131.6 (Ph), 128.3 (Ph), 121.7 (CBr), 83.4 (C(CH$_3$)$_2$)), 76.8 (PhCPh), 24.7 (C(CH$_3$)$_2$). **^{11}B NMR** (128 MHz, $CDCl_3$, δ, ppm): 22.42. **MS (EI) [m/z (%)]**: 468(5), 410(5), 278(44), 277(94), 201(28), 199(16), 185(35), 183(55), 165(23), 152(14), 129(11), 84(22), 82(19), 77(100), 63(11). **Elem. Anal**. calcd (%) for $C_{19}H_{21}BBr_2O_3$: C, 48.76; H, 4.52; found C, 59.09; H, 4.50. White solid. Isolation yield 99% (231.6 mg). The compound **3q** was synthesized and characterized for the first time.

*2-(1-((4,4,5,5-Tetramethyl-1,3,2-dioxaborolan-2-yl)oxy)ethyl)pyridine (**3u**)*

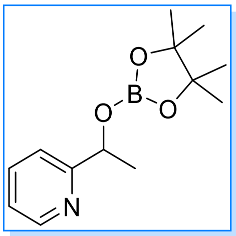

^{1}H NMR (300 MHz, $CDCl_3$, δ, ppm): 8.67 – 8.57 (m, 1H, Ph), 8.03 – 7.92 (m, 1H, Ph), 7.53 – 7.43 (m, 2H, Ph), 5.25 (q, $J_{H\text{-}H}$ = 6.6 Hz, 1H, CH(CH$_3$)), 1.58 (d, $J_{H\text{-}H}$ = 6.7 Hz, 3H, CH(CH$_3$)), 1.33 (s, 6H, C(CH$_3$)$_2$), 1.30 (s, 6H, C(CH$_3$)$_2$). **MS (EI) [m/z (%)]**: 249(2), 234(11), 191(73), 190(32), 150(54), 149(87), 148(35), 133(40), 132(47), 105(100), 79(41), 78(65). Characterized as a crude product. The analytical data are in agreement with the literature and were additionally supplemented with MS analysis.[14]

*4,4,5,5-Tetramethyl-2-(1-(thiophen-2-yl)ethoxy)-1,3,2-dioxaborolane (**3v**)*

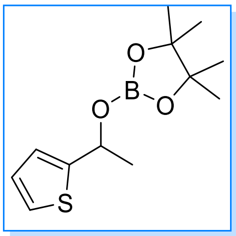

^{1}H NMR (300 MHz, $CDCl_3$, δ, ppm): 7.20 (dd, $J_{H\text{-}H}$ = 4.9, 1.4 Hz, 1H, CHS), 6.98 – 6.91 (m, 2H, CHCH), 5.48 (q, $J_{H\text{-}H}$ = 6.4, 1H, CH(CH$_3$), 1.60 (d, $J_{H\text{-}H}$ = 6.4 Hz, 3H, CH(CH$_3$)), 1.25 (s, 6H, C(CH$_3$)$_2$), 1.24 (s, 6H, C(CH$_3$)$_2$). **MS (EI) [m/z (%)]**: 254(17), 239(100), 196(27), 171(52), 157(11), 128(26), 111(87), 84(11), 57(10), 43(27). Characterized as a crude product. Analytical data are in agreement with the literature and were additionally supplemented with MS analysis .[15]

*2-(1-(Furan-2-yl)ethoxy)-4,4,5,5-tetramethyl-1,3,2-dioxaborolane (**3w**)*

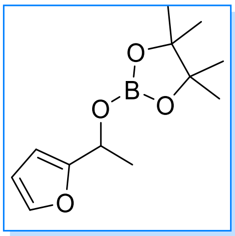

1H NMR (400 MHz, CDCl3, δ, ppm): δ 7.32 (s, 1H, CHO), 6.31 – 6.24 (m, 1H, CHCH), 6.24 – 6.18 (m, 1H, CHCH), 5.23 (q, $J_{H\text{-}H}$ = 6.6 Hz, 1H, CH(CH_3), 1.54 – 1.51 (m, 3H, CH(CH_3)), 1.24 (s, 12H, C(CH_3)$_2$). **MS (EI) [m/z (%)]:** 254(15), 237(10), 223(62), 195(16), 155(52), 129(76), 95(100), 94(39), 67(22), 43(39). Characterized as a crude product. The analytical data are in agreement with the literature and were additionally supplemented with MS analysis.[15]

*1-Phenylethan-1-ol (**5a**)*

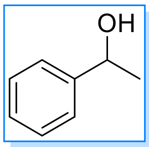

^{1}H NMR (300 MHz, $CDCl_3$, δ, ppm): 7.37 – 7.27 (m, 4H, Ph), 7.26 – 7.19 (m, 1H, Ph), 4.86 (q, $J_{H\text{-}H}$ = 6.5 Hz, 1H, CH(CH_3)), 1.77 (s, 1H, OH), 1.46 (d, $J_{H\text{-}H}$ = 6.5 Hz, 3H, CH(CH_3)). **MS (EI) [m/z (%)]**: 122(21), 107(80), 105(12), 80(7), 79(100), 78(27), 77(63), 64(3), 52(6), 50(24). Colorless liquid. Isolation yield 83% (50.6 mg). Analytical data are in agreement with the literature.[16,17]

*1–(2–Tolyl)ethan-1-ol (**5b**)*

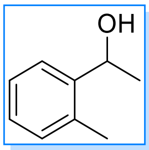

^{1}H NMR (400 MHz, $CDCl_3$, δ, ppm): 7.45 (d, $J_{H\text{-}H}$ = 7.3 Hz, 1H, Ph), 7.20 – 7.15 (m, 1H, Ph), 7.14 – 7.05 (m, 2H, Ph), 5.06 (q, $J_{H\text{-}H}$ = 6.4 Hz, 1H, CH(CH_3)), 2.28 (s, 3H, *o*-CH_3), 1.80 (brs, 1H, OH), 1.40 (d, $J_{H\text{-}H}$ = 6.4 Hz, 3H, CH(CH_3)). **MS (EI) [m/z (%)]**: 136(5), 121(74), 118,(64), 93(100), 91(91), 77 (47), 65(28), 63 (9), 51(14). Colorless liquid. Isolation yield 78% (53 mg). Analytical data are in agreement with the literature.[16]

*1–(3–Tolyl)ethan-1-ol (**5c**)*

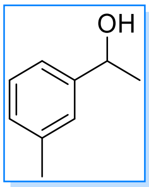

¹H NMR (300 MHz, CD_2Cl_2, δ, ppm): 7.30 – 7.12 (m, 4H, Ph), 4.82 (q, J_{H-H} = 6.5 Hz, 1H, C<u>H</u>(CH_3)), 2.82 (s, 1H, O<u>H</u>), 2.41 (s, 3H, *m*-C<u>H</u>3), 1.47 (d, J_{H-H} = 6.5 Hz, 3H, CH(C<u>H</u>3)). **MS (EI) [m/z (%)]**: 136(27), 121(58), 93(100), 92(25), 91(75), 78(7), 65(22), 60(11), 51(11). Colorless liquid. Isolation yield 62% (42.2 mg). Analytical data are in agreement with the literature.[16,18]

*1–(4–Tolyl)ethan-1-ol (**5d**)*

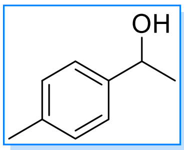

¹H NMR (300 MHz, $CDCl_3$, δ, ppm): 7,19 (d, J_{H-H} = 8.1 Hz 2H, Ph), 7.09 (d, J_{H-H} = 7.9 Hz, 2H, Ph), 4.78 (q, J_{H-H} = 6.4 Hz, 1H, C<u>H</u>(CH_3)), 2.28 (s, 3H, *p*-C<u>H</u>3), 1.91 (s, 1H, O<u>H</u>), 1.41 (d, J_{H-H} = 6.5 Hz, 3H, CH(C<u>H</u>3)). **MS (EI) [m/z (%)]**: 136(30), 121(100), 118(8), 115(9), 93(82), 91(84), 78(14), 77(33), 65(22), 63(13), 51(20). Colorless liquid. Isolation yield 75% (51 mg). Analytical data are in agreement with the literature.[16]

*1–(4–Methoxyphenyl)propan–1–ol (**5e**)*

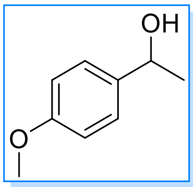

¹H NMR (300 MHz, $CDCl_3$, δ, ppm): 7.32 – 7.27 (m, 2H, Ph), 6.90 – 6.86 (m, 2H, Ph), 4.84 (q, J_{H-H} = 6.4 Hz, 1H, C<u>H</u>(CH_3)), 3.80 (s, 3H, *p*-OC<u>H</u>3), 1.89 (brs, 1H, O<u>H</u>), 1.47 (d, J_{H-H} = 6.4 Hz, 3H, CH(C<u>H</u>3)). **MS (EI) [m/z (%)]**: 152(24), 137(100), 134(13), 119(9), 109(66), 94(39), 91(17), 77(38), 65(17), 51(9). Colorless liquid. Isolation yield 82% (62.4 mg). Analytical data are data in agreement with the literature.[16]

*2,2-Dimethyl-1-phenylpropan-1-ol (**5f**)*

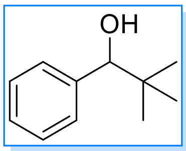

¹H NMR (400 MHz, $CDCl_3$, δ, ppm): 7.36 – 7.27 (m, 5H, Ph), 4.42 (s, 1H, C<u>H</u>(CH_3)), 1.85 (brs, 1H, O<u>H</u>), 0.95 (s, 9H, (C(C<u>H</u>3)3). **MS (EI) [m/z (%)]**: 164(3), 107(100), 79(57), 77(26), 57(16), 51(6). Pale yellow solid. Isolation yield 70% (57.5 mg). Analytical data are in agreement with the literature.[19]

*1–(2–Fluorophenyl)ethan-1-ol (**5g**)*

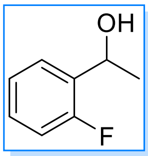

^{1}H NMR (300 MHz, CDCl3, δ, ppm): 7.52 – 7.46 (m, 1H, Ph), 7.29 – 7.20 (m, 1H, Ph), 7.18 – 7.12 (m, 1H, Ph), 7.05 – 6.99 (m, 1H, Ph), 5.20 (q, $J_{H\text{-}H}$ = 6.5 Hz, 1H, CH(CH_3)), 1.83 (s, 1H, OH), 1.52 (d, $J_{H\text{-}H}$ = 6.9 Hz, 3H, CH(CH_3)). **MS (EI) [m/z (%)]**: 140(18), 126(7), 124(100), 97(66), 96(11), 95(8), 77(34), 75(10), 62(4), 51(12). Colorless liquid. Isolation yield 60% (42.5 mg). Analytical data are in agreement with the literature.[20,21]

*1–(4–Fluorophenyl)ethan-1-ol (**5h**)*

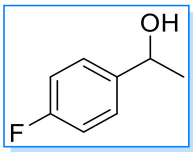

^{1}H NMR (300 MHz, $CDCl_3$, δ, ppm): 7.25 – 7.14 (m, 4H, Ph), 4.78 (q, $J_{H\text{-}H}$ = 6.4 Hz, 1H, CH(CH_3)), 1.75 (s, 1H, OH), 1.39 (d, $J_{H\text{-}H}$ = 6.5 Hz, 3H, CH(CH_3)). **MS (EI) [m/z (%)]**: 140(14), 125(100), 98(91), 96(44), 83(7), 77(44), 71(9), 57(13), 51 (18). Colorless liquid. Isolation yield 60% (42 mg). Analytical data are in agreement with the literature.[16,22]

*1–(2–Chlorophenyl)ethan-1-ol (**5i**)*

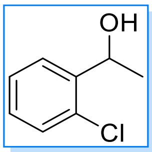

^{1}H NMR (300 MHz, $CDCl_3$, δ, ppm): 7.60 (dd, $J_{H\text{-}H}$ = 7.6 Hz, 1.7 Hz, 1H, Ph), 7.35 – 7.27 (m, 2H, Ph), 7.23 – 7.17 (m, 1H, Ph), 5.30 (q, $J_{H\text{-}H}$ = 6.4 Hz, 1H, CH(CH_3)), 1.85 (s, 1H, OH), 1.50 (d, $J_{H\text{-}H}$ = 6.4 Hz, 3H, CH(CH_3)). **MS (EI) [m/z (%)]**: 155(15), 141(86), 113(33), 77(100), 75 (12), 70(6), 51(25). Colorless liquid. Isolation yield 85% (66.6 mg). Analytical data are in agreement with the literature.[3,23]

*1–(4–Bromophenyl)ethan-1-ol (**5j**)*

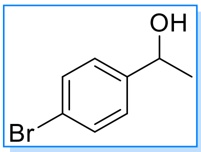

^{1}H NMR (400 MHz, $CDCl_3$, δ, ppm): 7.46 (d, $J_{H\text{-}H}$ = 8.4 Hz, 2H, Ph), 7.23 (d, $J_{H\text{-}H}$ = 8.4 Hz, 2H, Ph), 4.84 (q, $J_{H\text{-}H}$ = 6.4 Hz, 1H, CH(CH_3)), 2.04 (s, 1H, OH), 1.46 (d, $J_{H\text{-}H}$ = 6.5 Hz, 3H, CH(CH_3)). **MS (EI) [m/z (%)]**: 202(25), 201(4), 200(25), 187(76), 185(85), 159(21), 157(25), 121(18), 104(6), 78(47), 77(100), 63(6), 51(34). Colorless liquid. Isolation yield 74% (74.4 mg). Analytical data are in agreement with the literature.[19,24]

*1–(4–Iodophenyl)ethan-1-ol (**5k**)*

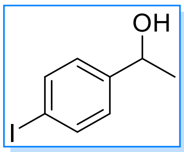

^{1}**H NMR** (300 MHz, $CDCl_3$, δ, ppm): 7.72-7.62 (m, 2H, Ph), 7.17-7.07 (m, 2H, Ph), 4.85 (q, $J_{H\text{-}H}$ = 6.5 Hz, 1H, C<u>H</u>(CH_3)), 1.80 (s, 1H, O<u>H</u>), 1.46 (d, $J_{H\text{-}H}$ = 6.5 Hz, 3H, CH(C<u>H</u>$_3$)). **MS (EI) [m/z (%)]**: 248(47), 233(90), 205(10), 127(9), 121(10), 116(8), 105(14), 91(7), 78(100), 77(37), 51(30), 50(21). Yellow solid. Isolation yield 68% (84.3 mg). Analytical data are in agreement with the literature.[25]

*4-(1-Hydroxyethyl)benzonitrile (**5l**)*

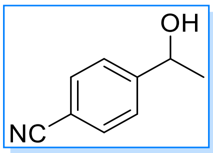

^{1}**H NMR** (300 MHz, $CDCl_3$, δ, ppm): 7.63 (d, $J_{H\text{-}H}$ = 8.3 Hz, 2H, Ph), 7.48 (d, $J_{H\text{-}H}$ = 8.2 Hz, 2H, Ph), 4.96 (q, $J_{H\text{-}H}$ = 6.5 Hz, 1H, C<u>H</u>(CH_3)), 1.95 (s, 1H, O<u>H</u>), 1.49 (d, $J_{H\text{-}H}$ = 6.5 Hz, 3H, CH(C<u>H</u>$_3$)). **MS (EI) [m/z (%)]**: 147(11), 132(100), 104(69), 77(21), 76(12), 51(14). Colorless liquid. Isolation yield 80% (58.9 mg). Analytical data are in agreement with the literature.[26]

*Methyl 4-(1-hydroxyethyl)benzoate (**5m**)*

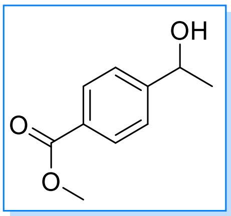

^{1}**H NMR** (400 MHz, $CDCl_3$, δ, ppm): δ 8.01 – 7.95 (m, 2H, Ph), 7.43 – 7.39 (m, 2H, Ph), 4.93 (q, $J_{H\text{-}H}$ = 6.5 Hz, 1H, C<u>H</u>(CH_3)), 3.89 (s, 3H, OC<u>H$_3$</u>), 2.25 (brs, 1H, O<u>H</u>), 1.48 (d, $J_{H\text{-}H}$ = 6.5 Hz, 3H, CH(C<u>H$_3$</u>)). **MS (EI) [m/z (%)]**: 180(2), 165(55), 149(16), 137 (94), 132(8), 121(13), 104(45), 91(49), 77(100), 63(7), 63(7), 56(68), 51(30). Colorless liquid. Isolation yield 79% (71.2 mg). Analytical data are in agreement with the literature.[18]

*N-(4-(1-hydroxyethyl)phenyl)acetamide (**5n**)*

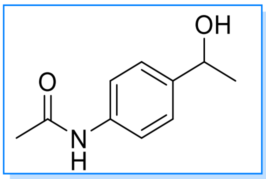

^{1}**H NMR** (300 MHz, $CDCl_3$, δ, ppm): 7.50 – 7.41 (m, 2H, Ph), 7.38 – 7.27 (m, 3H, Ph and NH(overlap)), 4.87 (q, $J_{H\text{-}H}$ 6.4 Hz, 1H, C<u>H</u>(CH_3), 2.16 (s, 3H, CO(C<u>H$_3$</u>)), 1.79 (s, 1H, O<u>H</u>), 1.47 (d, $J_{H\text{-}H}$ = 6.4 Hz, 3H, CH(C<u>H$_3$</u>)). **MS (EI) [m/z (%)]**: 179(2), 161(37), 119(100), 104(4), 91(40), 77(6), 65(19), 51(13). Colorless liquid. Isolation yield 75% (67.0 mg). Analytical data are in agreement with the literature.[27]

*1-Phenylpropane-1,2-diol (**5o**)*

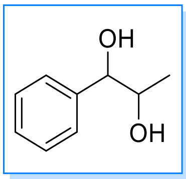

^{1}H NMR (300 MHz, $CDCl_3$, δ, ppm): 7.37 – 7.29 (m, 5H, Ph), 4.68 (d, $J_{H\text{-}H}$ = 4.4 Hz, 0.72H, C<u>H</u>CH-*syn*), 4.38 (d, $J_{H\text{-}H}$ = 7.3 Hz, 0.28H, C<u>H</u>CH-*anti*), 4.02 (m, 0.72H, CHC<u>H</u>-*syn*), 3.93 – 3.78 (m, 0.28H, CHC<u>H</u>-*anti*), 2.18 (s, 2H, O<u>H</u>), 1.08 (m, 2H, CC<u>H</u>$_3$).**MS (EI) [m/z (%)]**: 152(2), 149(3), 133(3), 119(3), 115(5), 108(54), 170(45), 90(8), 79(100), 77(55), 63(7), 51(24). Pale yellow solid. Isolation yield 70% (53.1 mg). Analytical data are in agreement with the literature.[28-30]

*1-(Naphthalen-2-yl)ethan-1-ol (**5p**)*

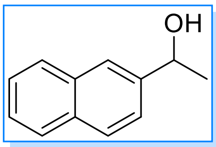

^{1}H NMR (400 MHz, $CDCl_3$, δ, ppm): 7.86 – 7.80 (m, 4H, Ph), 7.52 – 7.47 (m, 3H, Ph), 5.06 (q, $J_{H\text{-}H}$ = 6.4 Hz, 1H, C<u>H</u>(CH_3)), 2.00 (s, 1H, O<u>H</u>), 1.59 (d, $J_{H\text{-}H}$ = 6.5 Hz, 3H, CH(C<u>H</u>$_3$)). **MS (EI) [m/z (%)]**: 170(43), 155(84), 127(100), 126(19), 101(8), 77(19), 63(15), 51(10). White solid. Isolation yield 84% (72.3 mg). Analytical data are in agreement with the literature.[24,31]

*1-([1,1'-Biphenyl]-4-yl)ethan-1-ol (**5q**)*

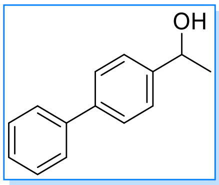

^{1}H NMR (400 MHz, $CDCl_3$, δ, ppm): 7.65 – 7.57 (m, 4H, Ph), 7.50 – 7.42 (m, 4H, Ph), 7.37 (t, $J_{H\text{-}H}$ = 7.3 Hz, 1H, Ph), 4.96 (q, $J_{H\text{-}H}$ = 6.5 Hz, 1H, C<u>H</u>(CH_3)), 2.05 (s, 1H, O<u>H</u>), 1.56 (d, $J_{H\text{-}H}$ = 6.5 Hz, 3H, CH(C<u>H</u>$_3$)). **MS (EI) [m/z (%)]**: 198(69), 183(100), 180(12), 155(99), 152(32), 115(9), 92(12), 77(31), 51(12). White solid. Isolation yield 93% (92.1 mg). Analytical data are in agreement with the literature.[32,33]

*Diphenylmethanol (**5r**)*

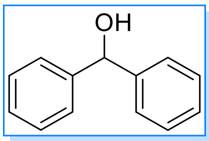

^{1}H NMR (400 MHz, $CDCl_3$, δ, ppm): 7.42 – 7.33 (m, 8H, Ph), 7.32 – 7.27 (m, 2H, Ph), 5.83 (s, 1H, C<u>H</u>(Ph_2)), 2.34 (s, 1H, O<u>H</u>). **MS (EI) [m/z (%)]**: 184(50), 183(16), 165(11), 152(4), 107(12), 105(100), 79(28), 78(41), 77(48), 51(18). White solid. Isolation yield 77% (70.9 mg). Analytical data are in agreement with the literature.[9,17]

*(2-Fluorophenyl)(phenyl)methanol (**5s**)*

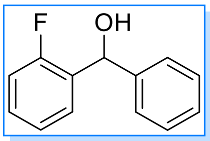

^{1}H NMR (400 MHz, $CDCl_3$, δ, ppm): 7.55 – 7.24 (m, 1H, Ph), 7.45 – 7.39 (m, 2H, Ph), 7.39 – 7.33 (m, 2H, Ph), 7.33 – 7.23 (m, 2H, Ph), 7.19 – 7.15 (m, 1H, Ph), 7.07 – 7.02 (m, 1H, Ph), 6.15 (s, 1H, C<u>H</u>$(Ph)_2$), 2.43 (s, 1H, O<u>H</u>). **MS (EI) [m/z (%)]**: 200(47), 180(7), 123(62), 105(100), 95(30), 77(58), 75(22), 51(23). Yellow liquid. Isolation yield 66% (66.7 mg). Analytical data are in agreement with the literature.[13,34]

*Bis-(4-bromophenyl)methanol (**5t**)*

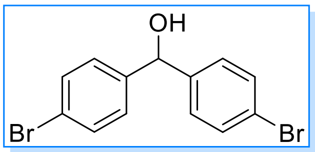

^{1}H NMR (300 MHz, $CDCl_3$, δ, ppm): 7.46 (d, $J_{H\text{-}H}$ = 8.4 Hz, 4H, Ph), 7.22 (d, $J_{H\text{-}H}$ = 8.4 Hz, 4H, Ph), 5.75 (s, 1H, C<u>H</u>$(Ph)_2$), 2.12 (brs, 1H, O<u>H</u>). **MS (EI) [m/z (%)]**: 342(11), 185(100), 183(99), 165(8), 157(17), 105(12), 78(20), 77(44), 76(20), 51(18). White solid. Isolation yield 78% (133.4 mg). Analytical data are in agreement with the literature.[22,32]

*1-(Pyridin-2-yl)ethan-1-ol (**5u**)*

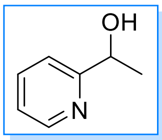

^{1}H NMR (300 MHz, $CDCl_3$, δ, ppm): 8.47 – 8.44 (m, 1H, Ar), 7.64 – 7.59 (m, 1H, Ar), 7.23 – 7.20 (m, 1H, Ar), 7.14 – 7.10 (m, 1H, Ar), 4.82 (q, $J_{H\text{-}H}$ = 6.5 Hz, 1H, C<u>H</u>(CH_3)), 4.07 (s, 1H, O<u>H</u>), 1.43 (d, $J_{H\text{-}H}$ = 6.6 Hz, 3H, CH(C<u>H</u>$_3$)). **MS (EI) [m/z (%)]**: 124(14), 122(10), 108(100), 1006(42), 104(7), 81(7), 79(30), 78(33), 52(27), 51(24). Colorless liquid. Isolation yield 84% (50.7 mg). Analytical data are in agreement with the literature.[35]

*1-(Thiophen-2-yl)ethan-1-ol (**5v**)*

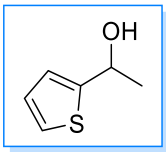

^{1}H NMR (300 MHz, $CDCl_3$, δ, ppm): 7.24 (dd, $J_{H\text{-}H}$ = 4.7, 1.6 Hz, 1H, C<u>H</u>S), 7.02 – 6.91 (m, 2H, CHC<u>H</u>), 5.14 (q, $J_{H\text{-}H}$ = 6.4 Hz, 1H, C<u>H</u>(CH_3)), 1.98 (bs, 1H, O<u>H</u>), 1.61 (d, $J_{H\text{-}H}$ = 6.4 Hz, 3H, CH(C<u>H</u>$_3$)). **MS (EI) [m/z (%)]**: 128(11), 113(45), 110(16), 95(13), 85(100), 84(10), 65(11), 59(8), 58(13), 51(10). Colorless oil. Isolation yield 77% (49.4 mg). Analytical data are in agreement with the literature.[35]

*1-(Furan-2-yl)ethan-1-ol (**5w**)*

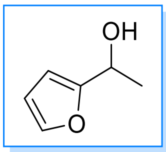

^{1}H NMR (300 MHz, $CDCl_3$, δ, ppm): 7.33 (dd, $J_{H\text{-}H}$ = 1.8, 0.8 Hz, 1H, C$\underline{H}$O), 6.30 – 6.28 (m, 1H, CHC$\underline{H}$), 6.19 – 6.18 (m, 1H, CHC$\underline{H}$), 4.82 (q, $J_{H\text{-}H}$ = 6.5 Hz, 1H, C$\underline{H}$(CH_3)), 2.77 (bs, 1H, O$\underline{H}$), 1.49 (d, $J_{H\text{-}H}$ = 6.6 Hz, 3H, CH(C$\underline{H_3}$)).**MS (EI) [m/z (%)]**: 112(23), 111(12), 97(66), 96(11), 95(100), 94(15), 84(10), 69(20), 43(22), 42(28). Colorless liquid. Isolation yield 81% (50.8 mg). Analytical data are in agreement with the literature.[36]

5. NMR spectra of products

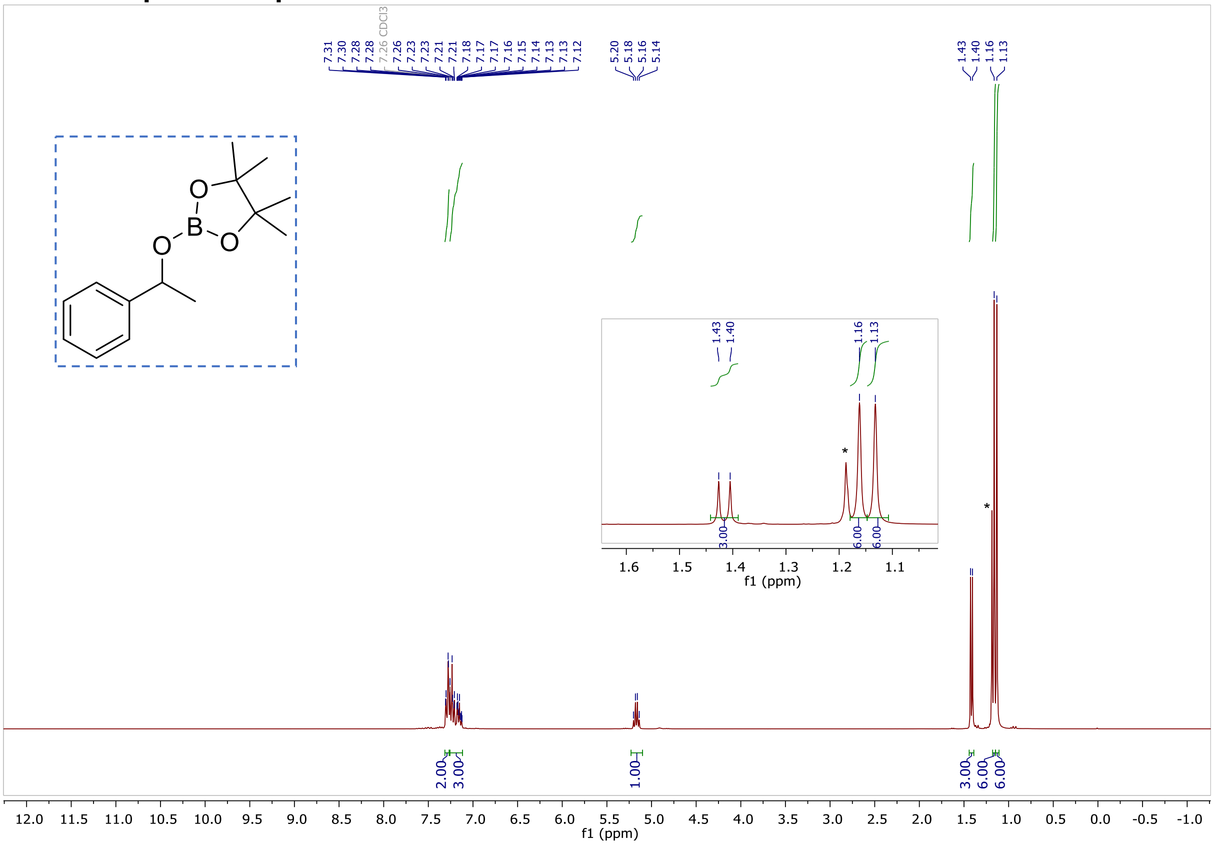

Figure S1 – ¹H NMR spectrum of **3a** from crude reaction mixture. *Excess of the HBpin.

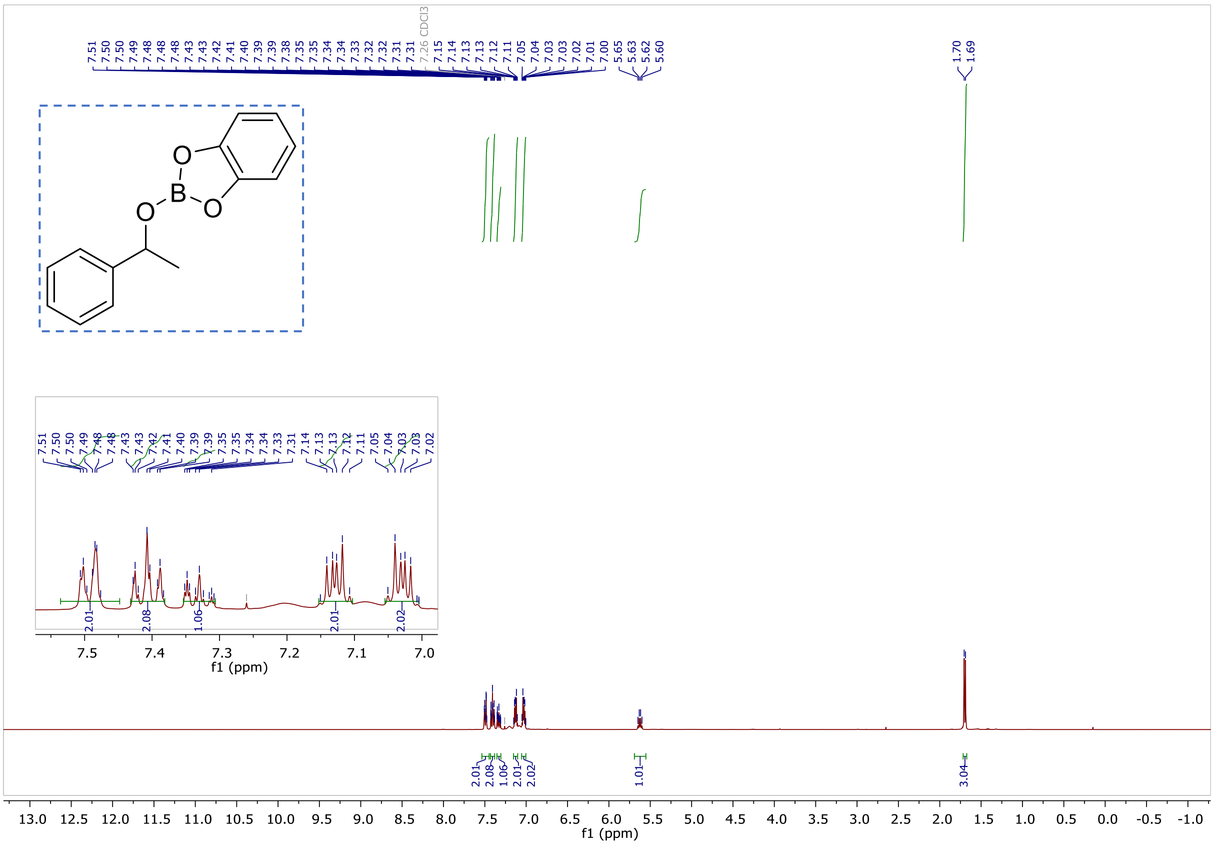

Figure S2 – ¹H NMR spectrum of **3a'**.

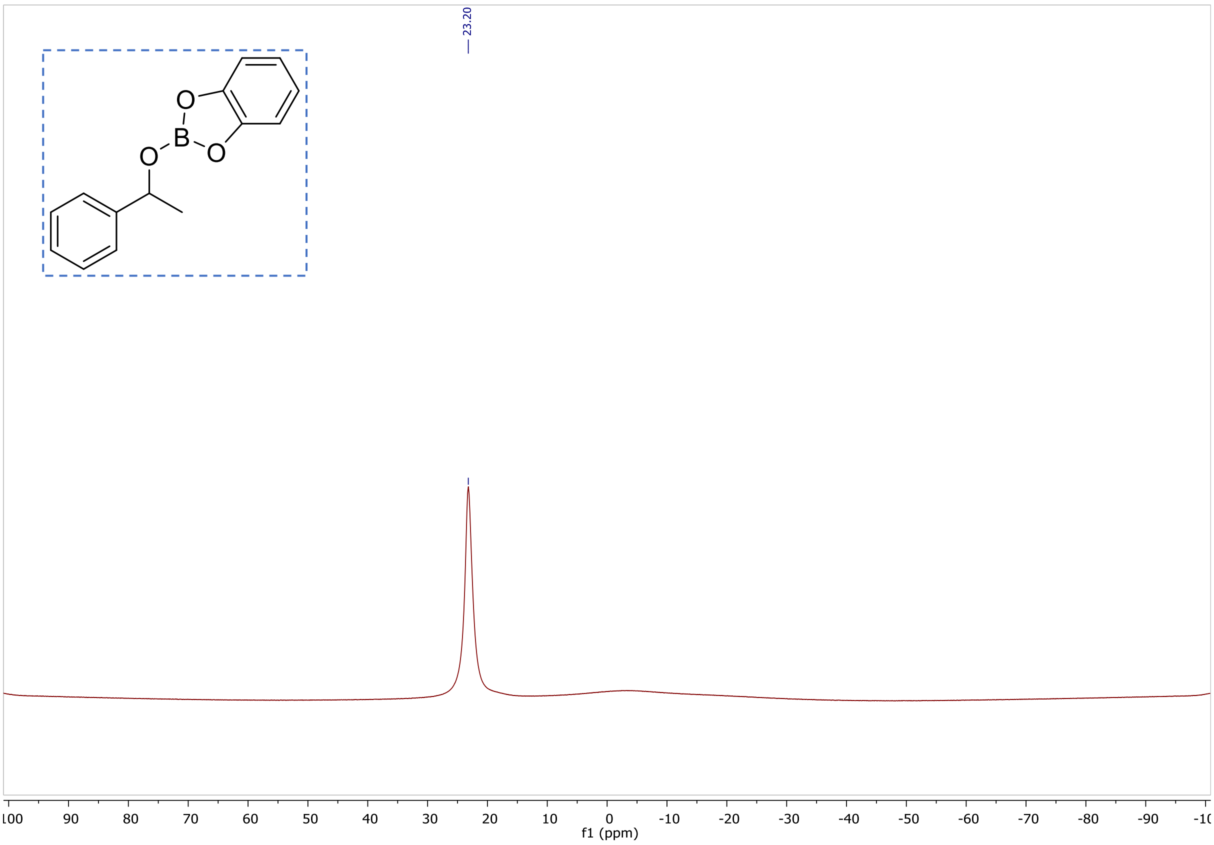

Figure S3 – ^{11}B NMR spectrum of **3a'**.

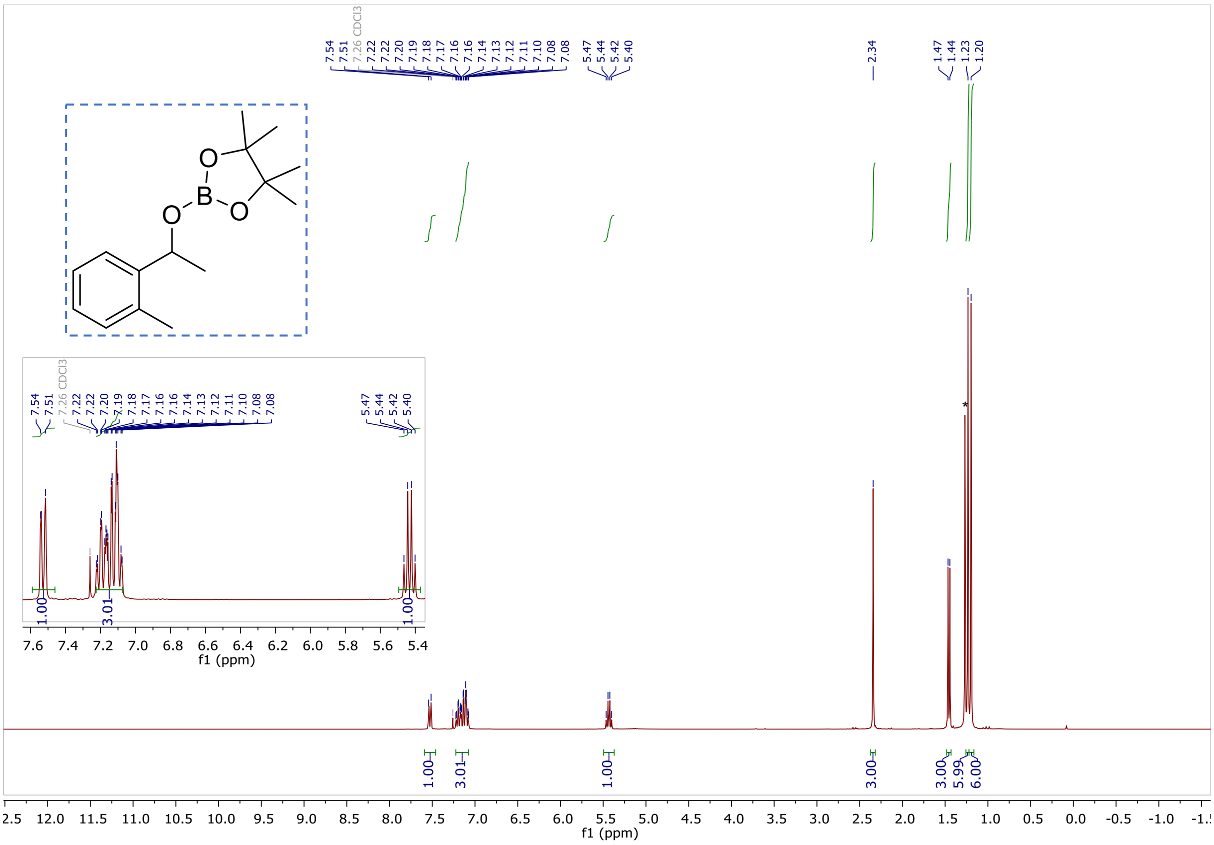

Figure S4 – ^{1}H NMR spectrum of **3b** from crude reaction mixture. *Excess of the HBpin.

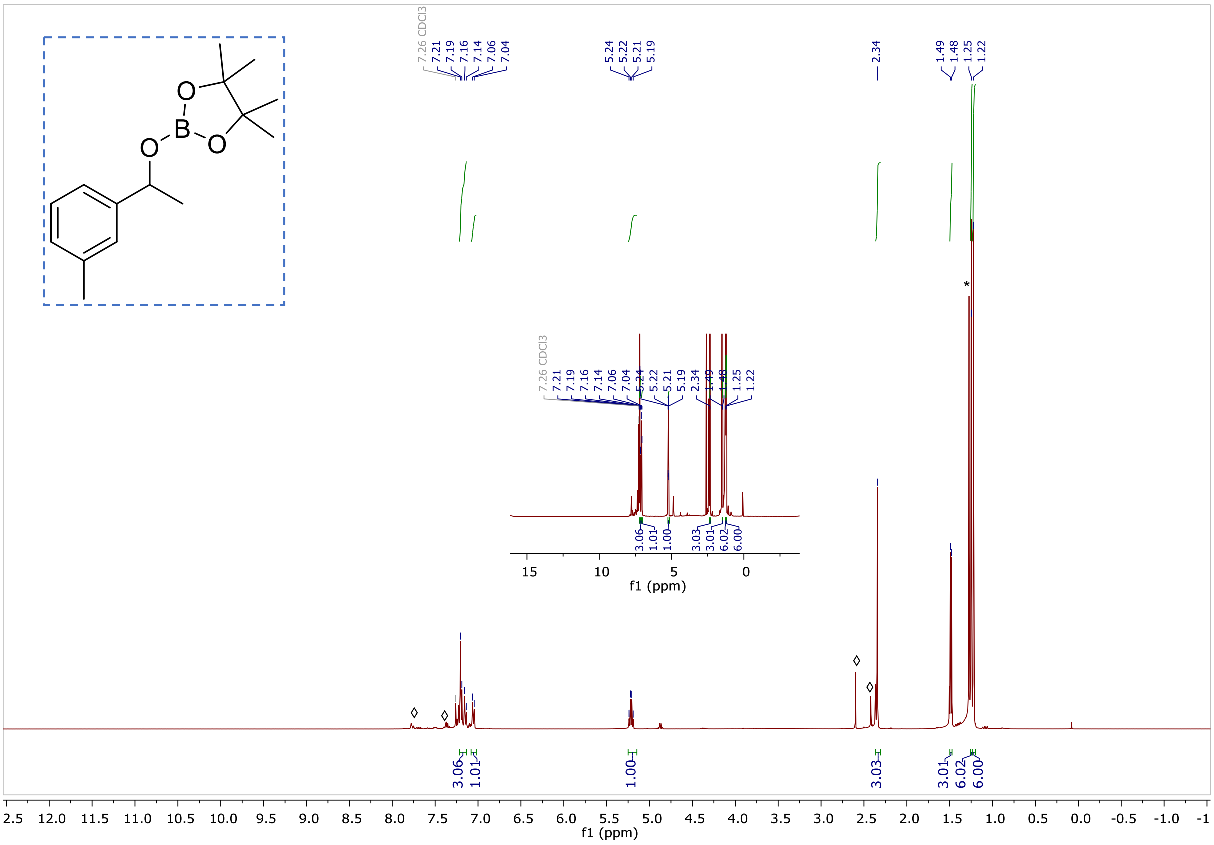

Figure S5 – ^{1}H NMR spectrum of **3c** from crude reaction mixture. *Excess of the HBpin. $^\lozenge$ Unreacted substrate. Conversion of **1c** = 86%.

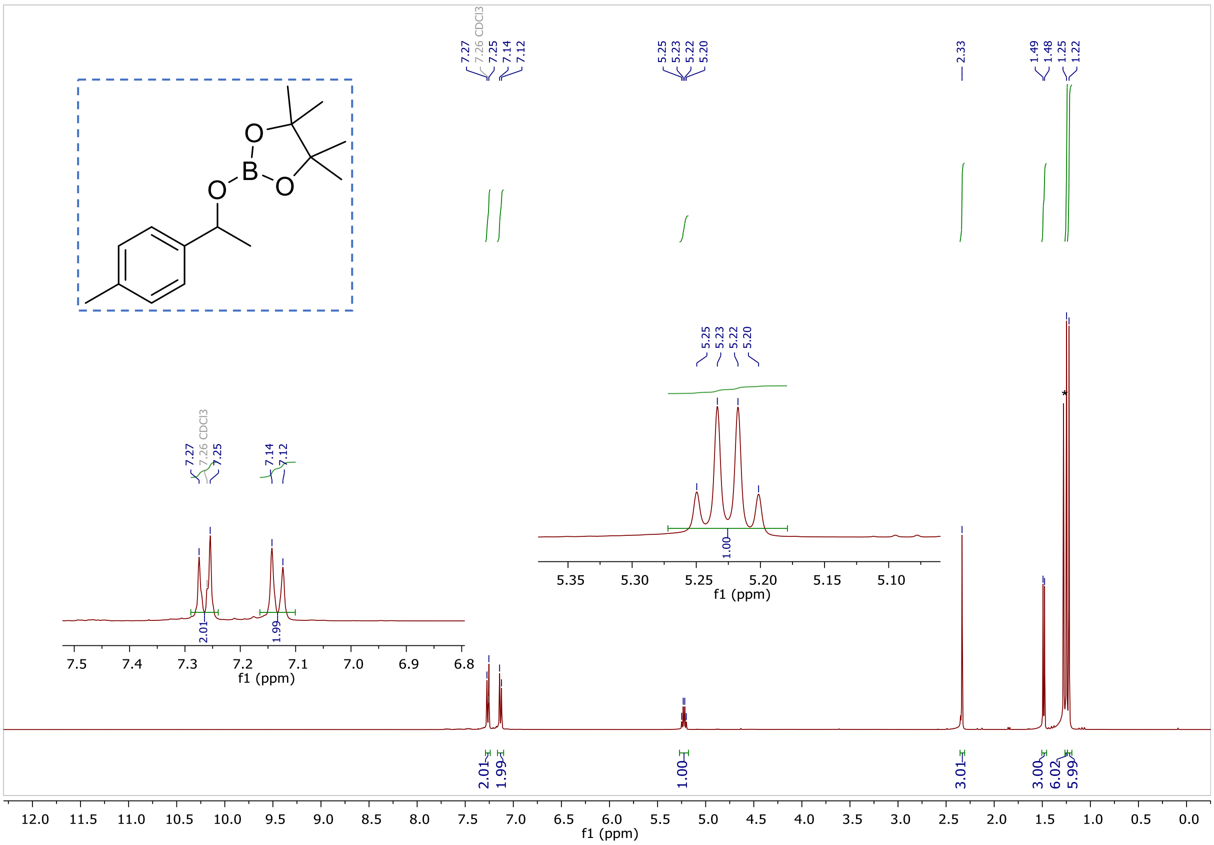

Figure S6 – ^{1}H NMR spectrum of **3d** from crude reaction mixture. *Excess of the HBpin.

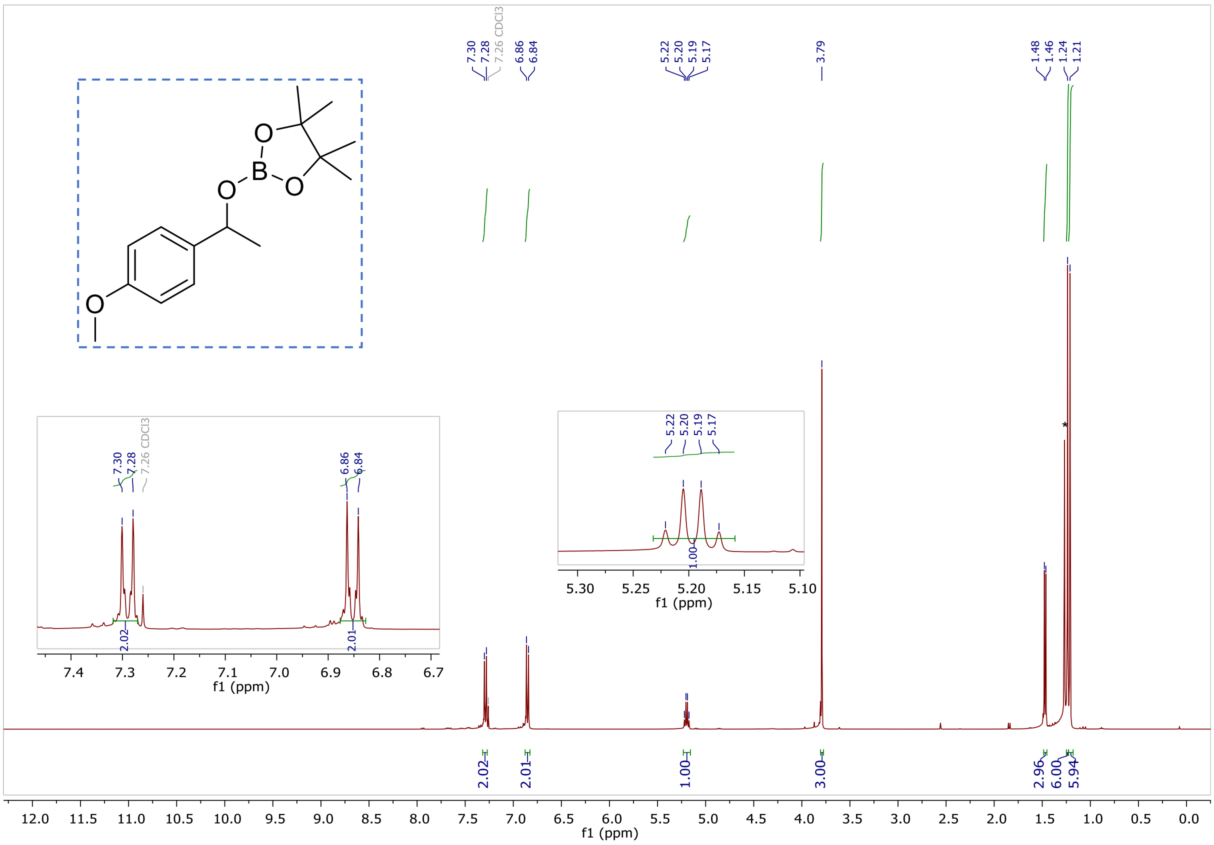

Figure S7 – ^{1}H NMR spectrum of **3e** from crude reaction mixture. *Excess of the HBpin.

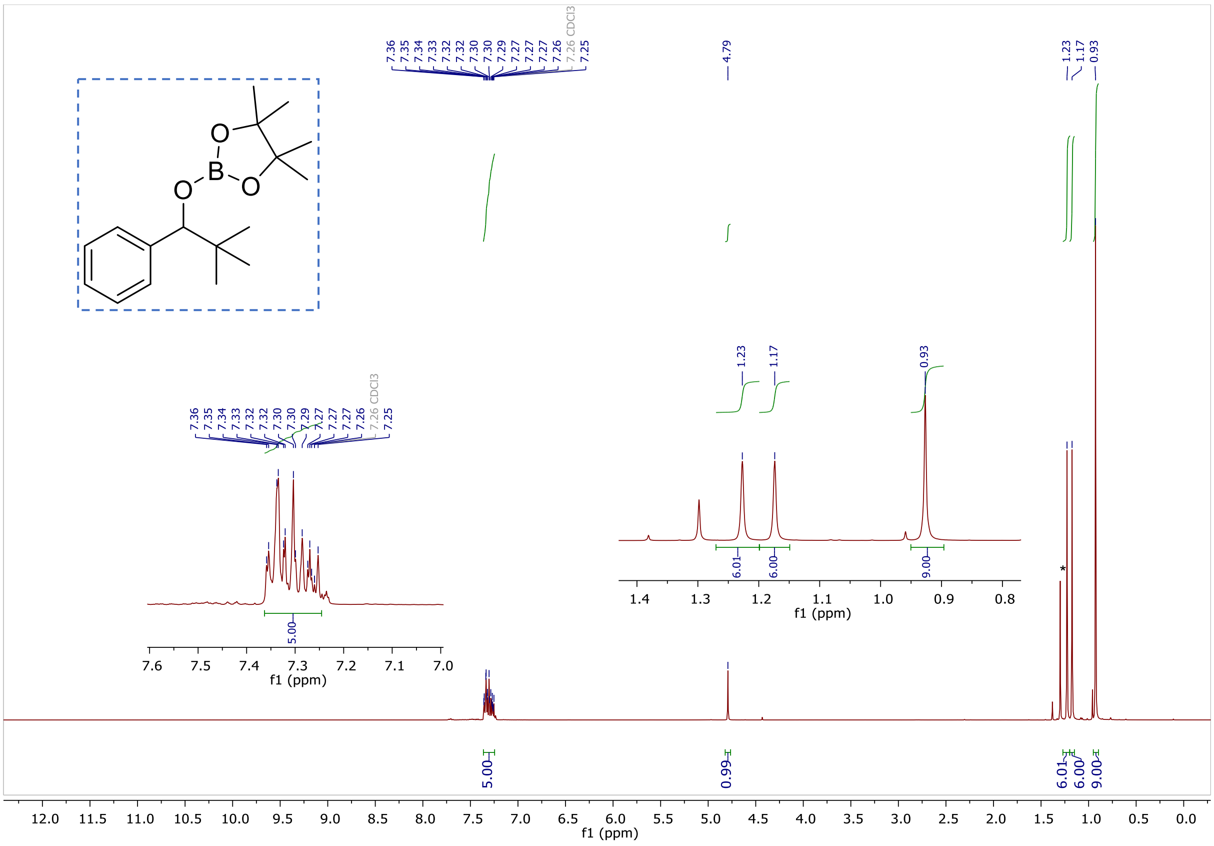

Figure S8 – ^{1}H NMR spectrum of **3f** from crude reaction mixture. *Excess of the HBpin.

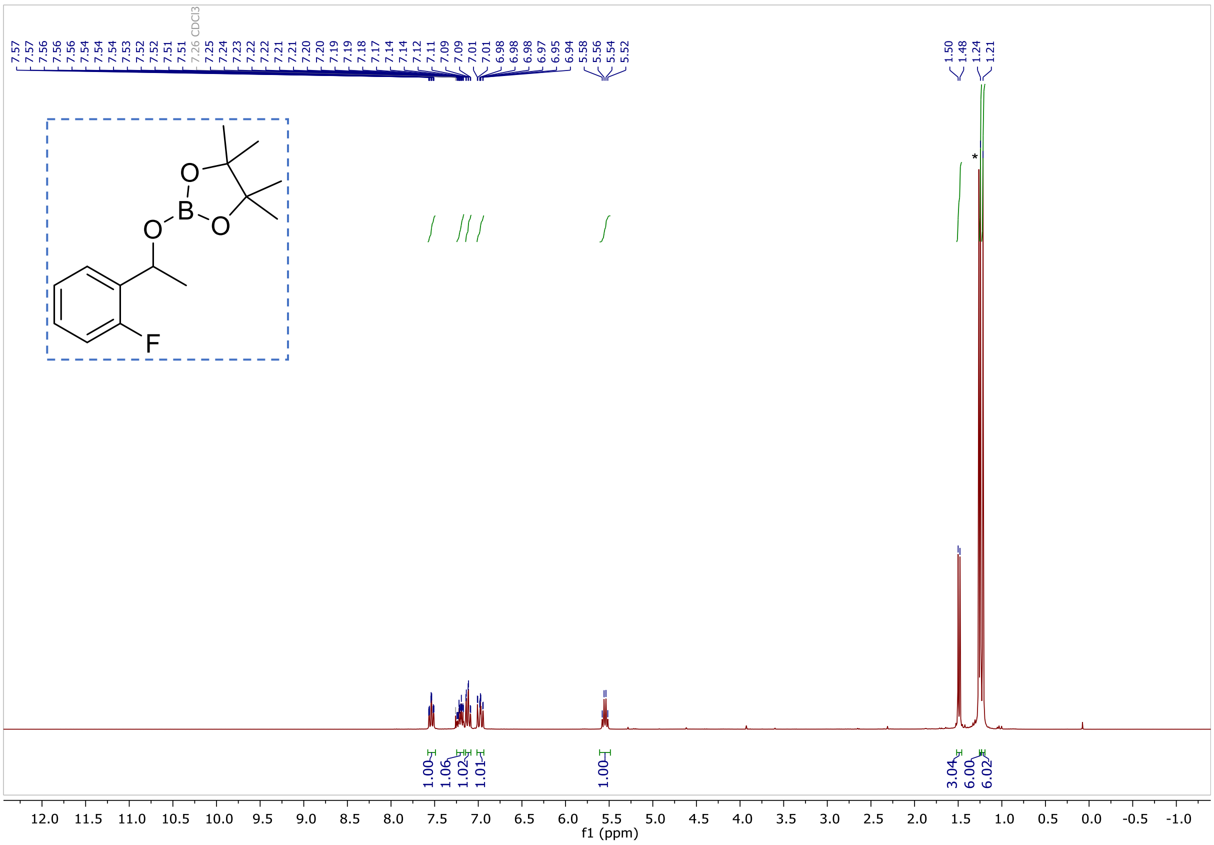

Figure S9 – ^{1}H NMR spectrum of **3g** from crude reaction mixture. *Excess of the HBpin.

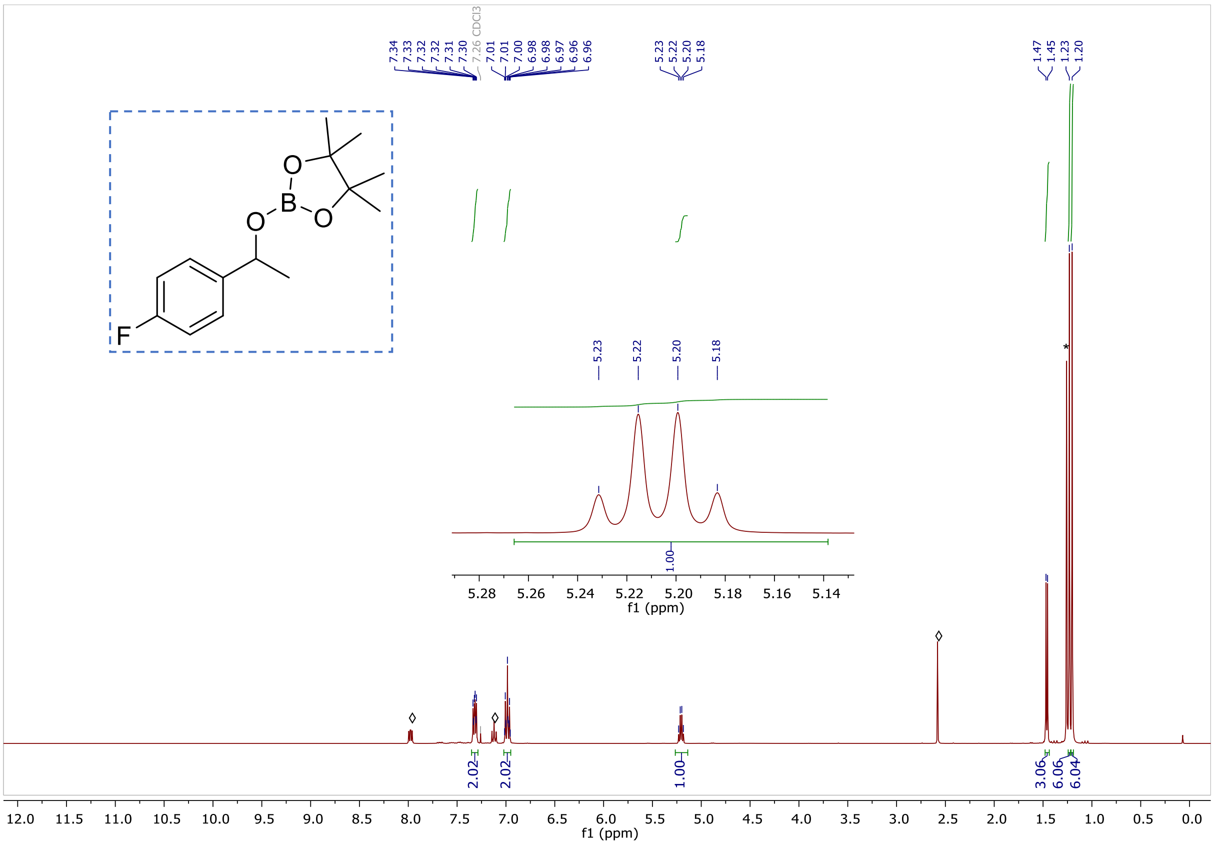

Figure S10 – ^{1}H NMR spectrum of **3h** from crude reaction mixture. *Excess of the HBpin. $^{\Diamond}$ Unreacted substrate. Conversion of **1h** = 78%.

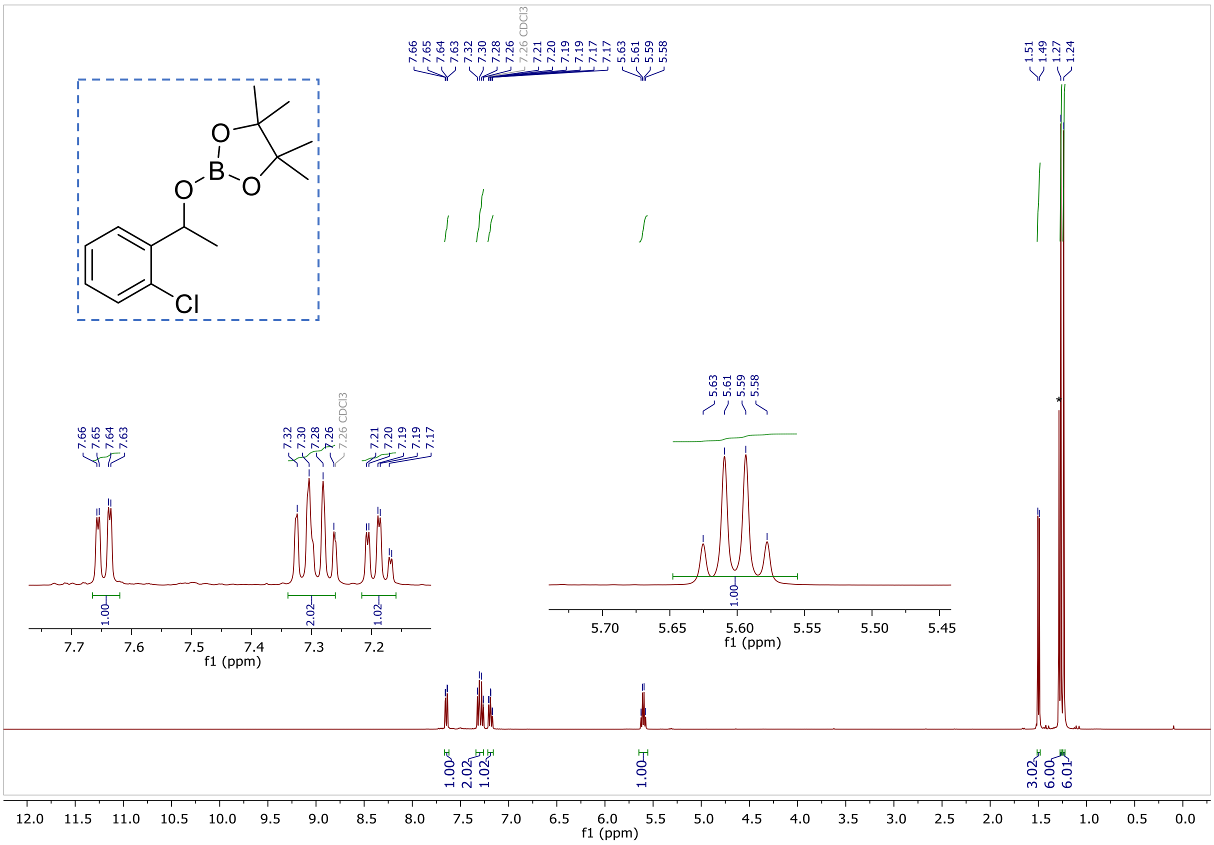

Figure S11 – ^{1}H NMR spectrum of **3i** from crude reaction mixture. *Excess of the HBpin.

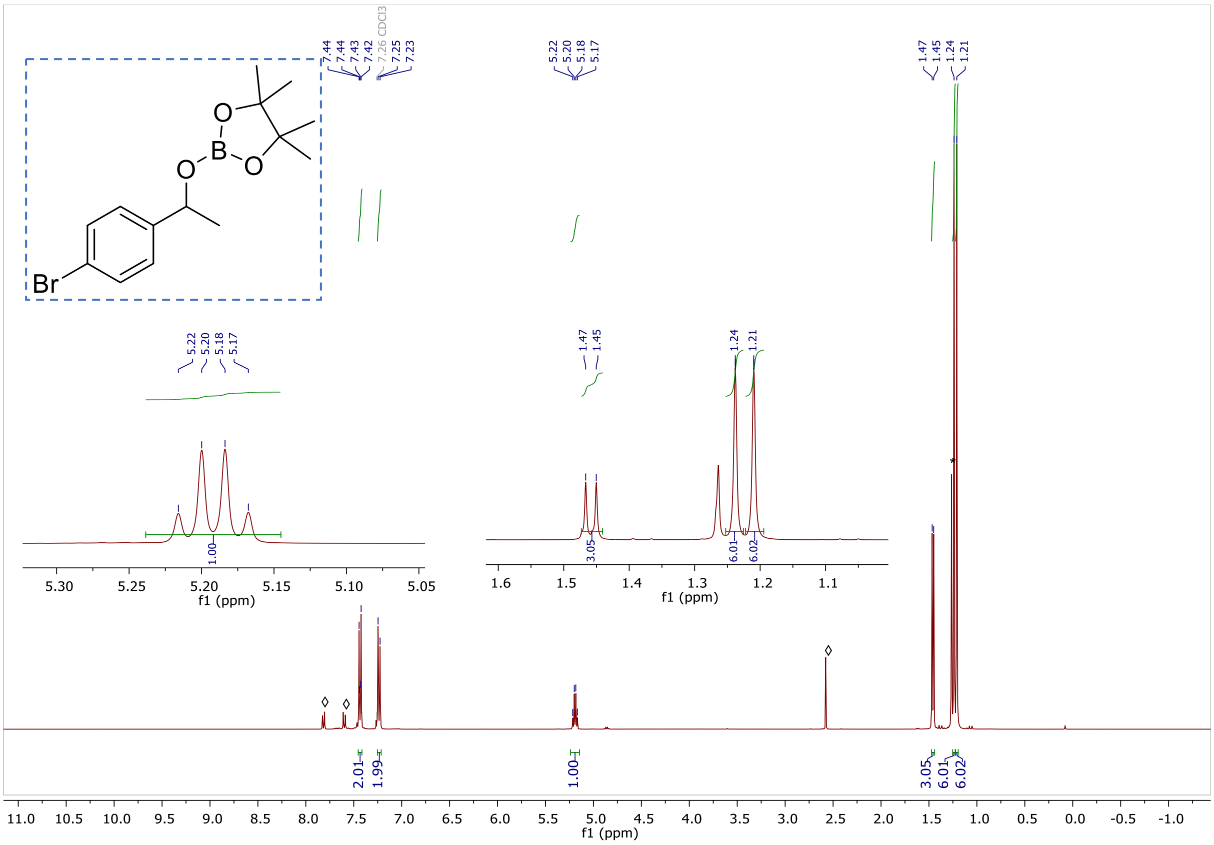

Figure S12 – ^{1}H NMR spectrum of **3j** from crude reaction mixture. *Excess of the HBpin. $^\lozenge$ Unreacted substrate. Conversion of **1j** = 89%.

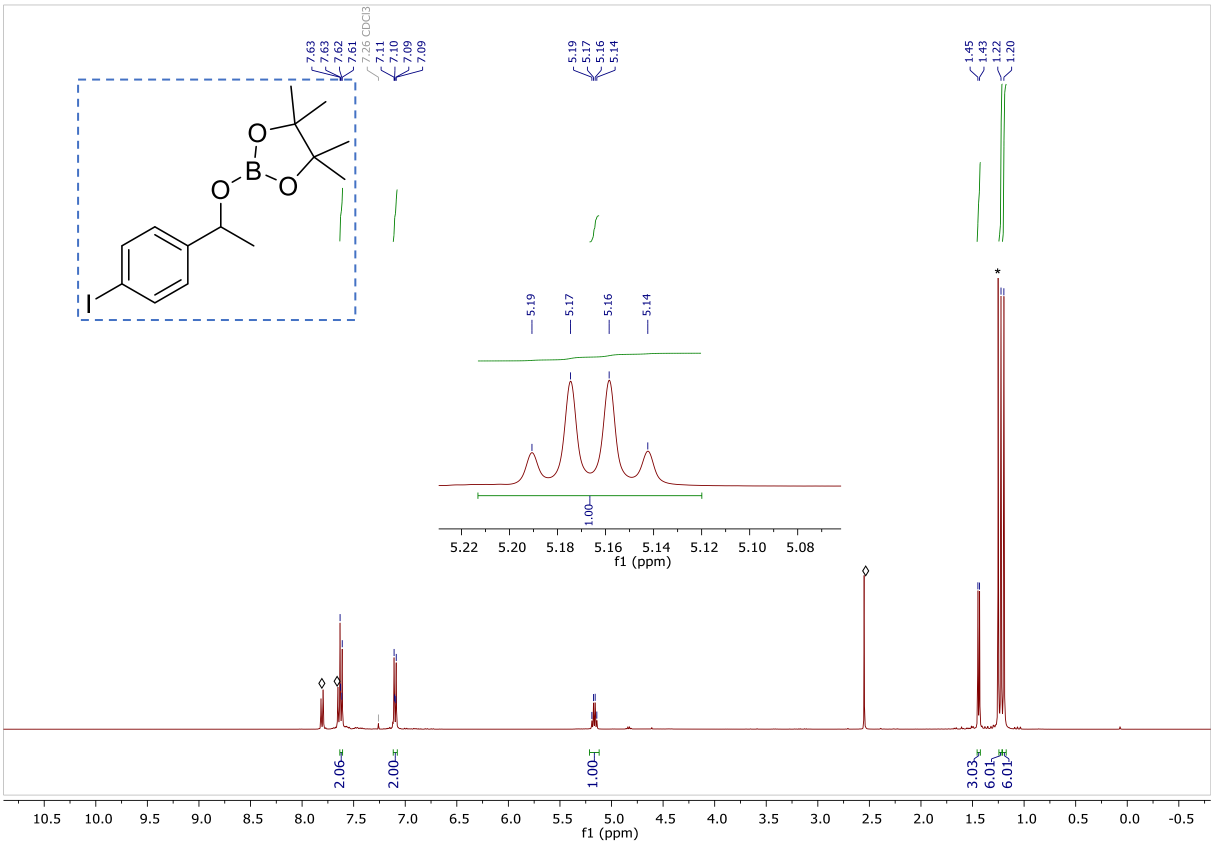

Figure S13 – ^{1}H NMR spectrum of **3k** from crude reaction mixture. *Excess of the HBpin. ◊ Unreacted substrate. Conversion of **1k** = 74%.

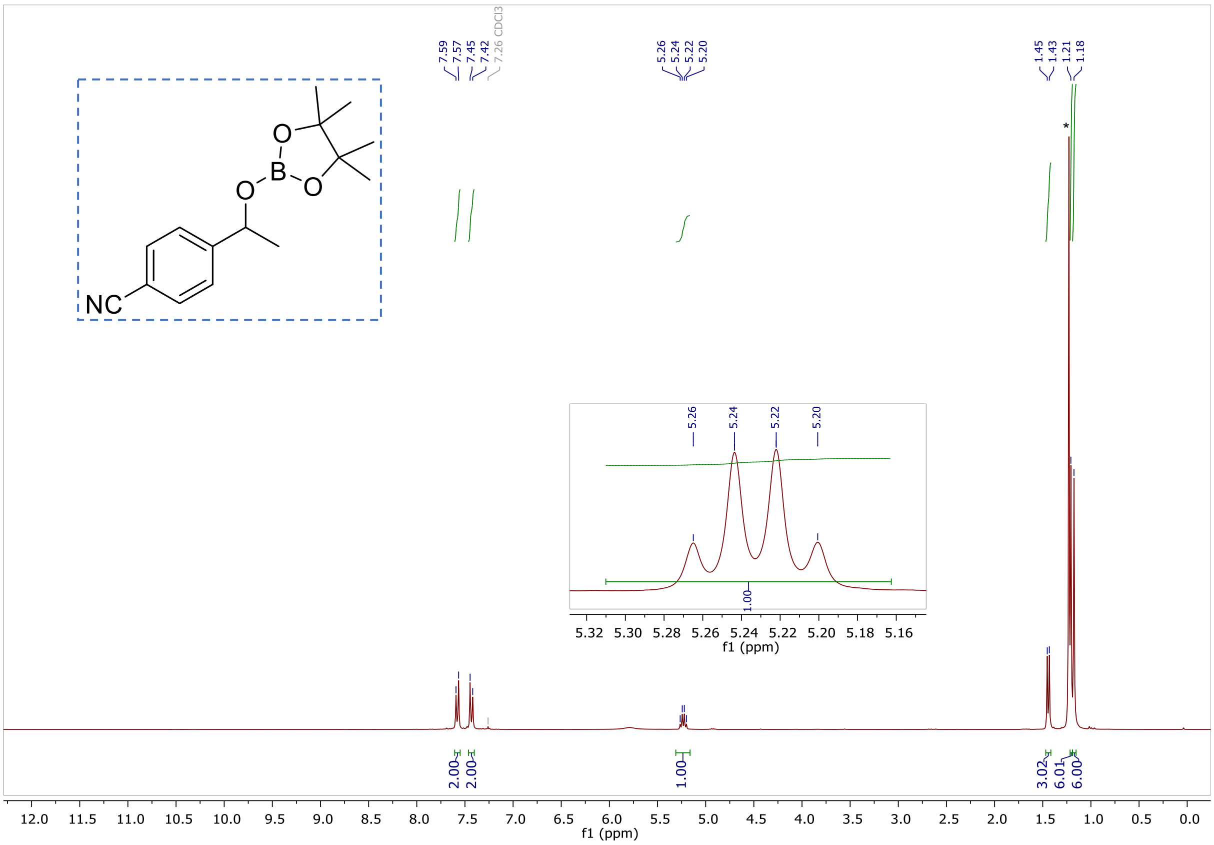

Figure S14 – ^{1}H NMR spectrum of **3l** from crude reaction mixture. *Excess of the HBpin.

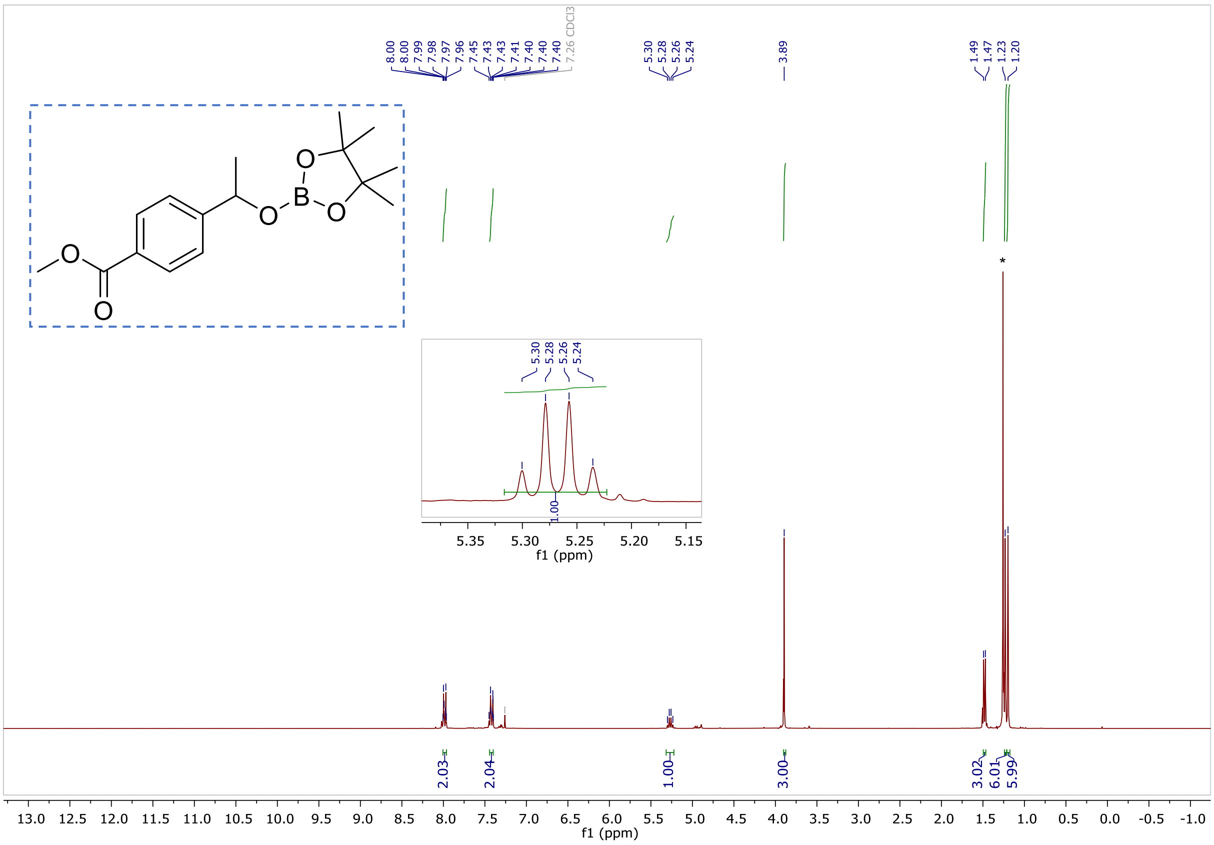

Figure S15 – ^{1}H NMR spectrum of **3m** from crude reaction mixture. *Excess of the HBpin.

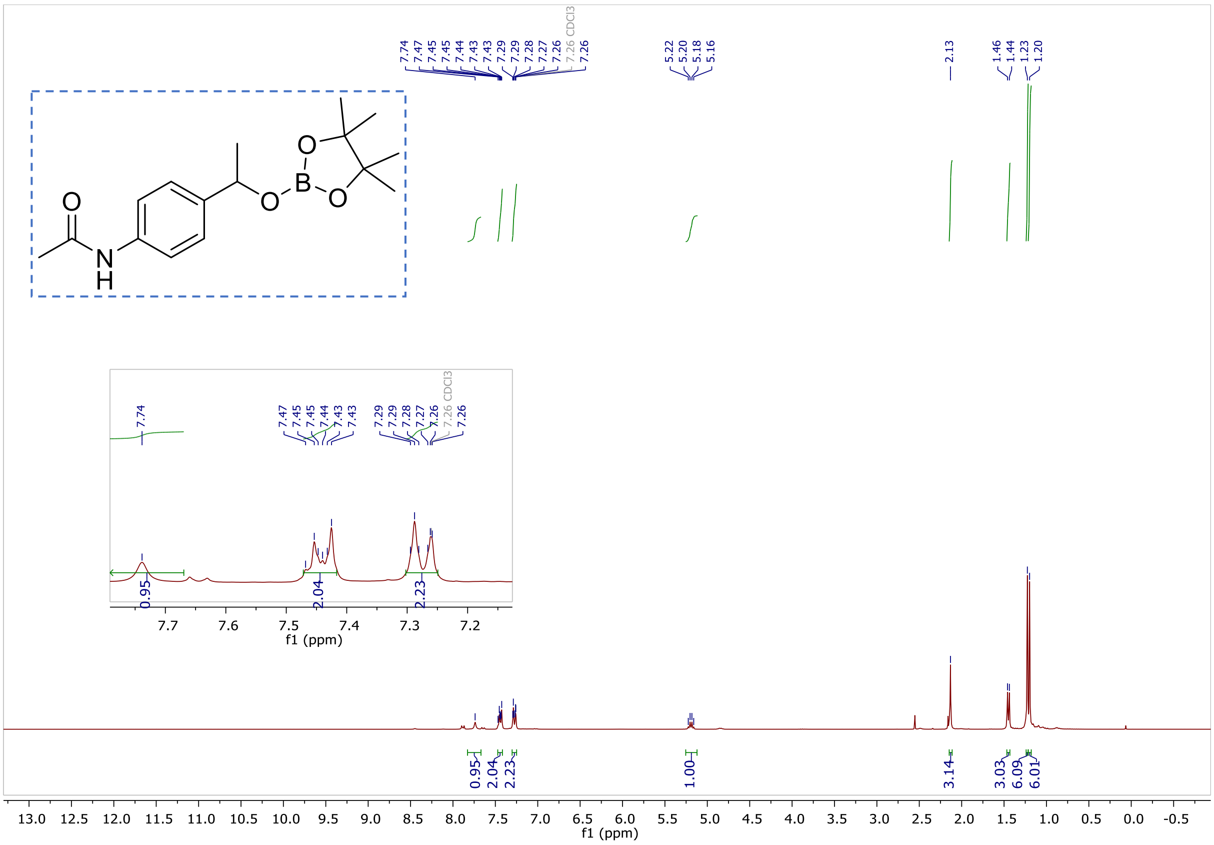

Figure S16 – ^{1}H NMR spectrum of **3n.**

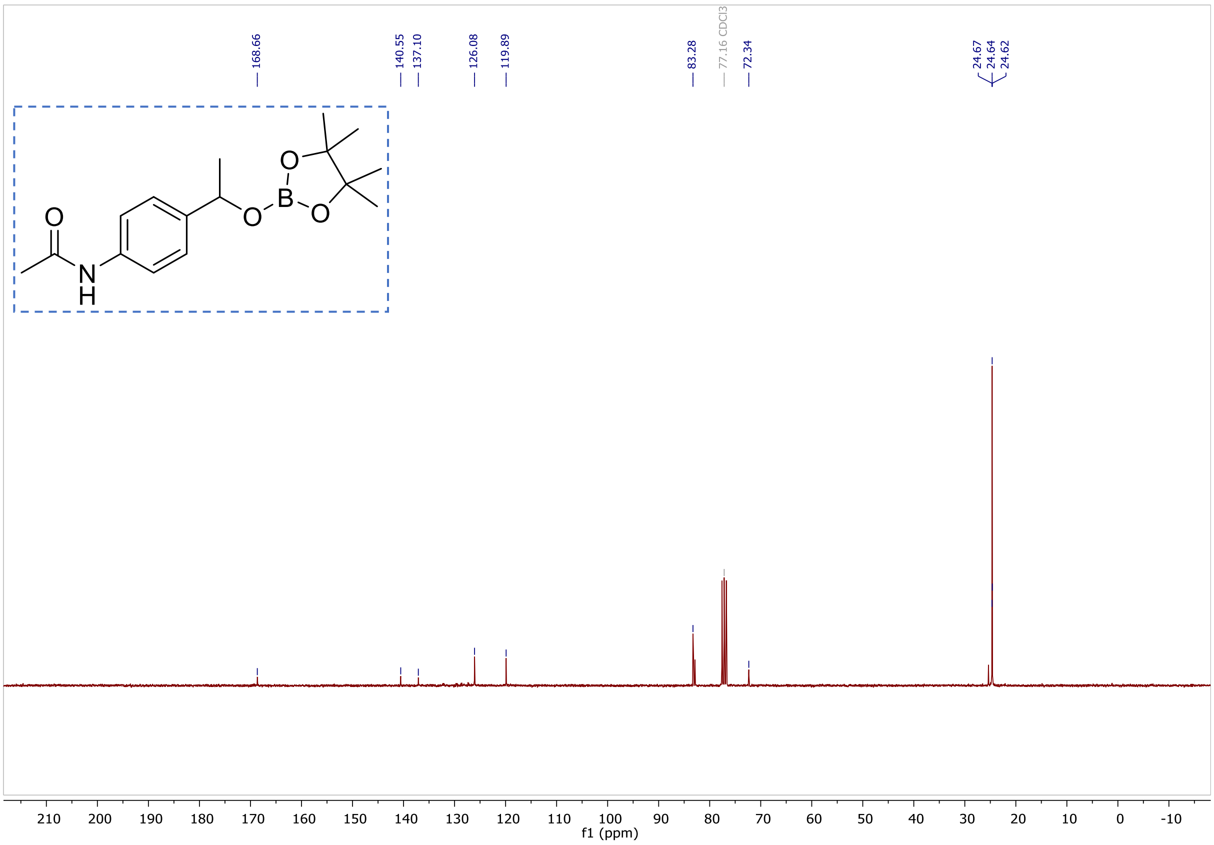

Figure S17 – ^{13}C NMR spectrum of **3n.**

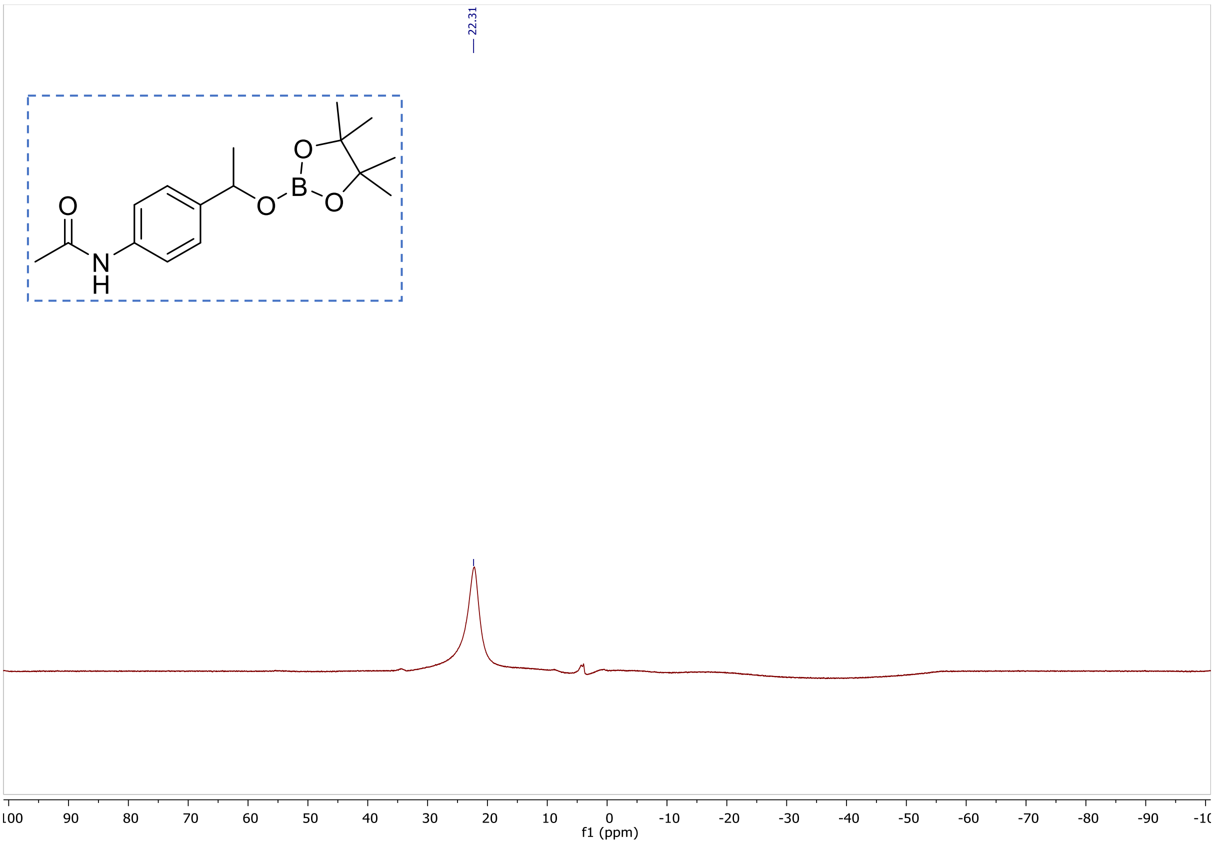

Figure S18 – ^{11}B NMR spectrum of **3n.**

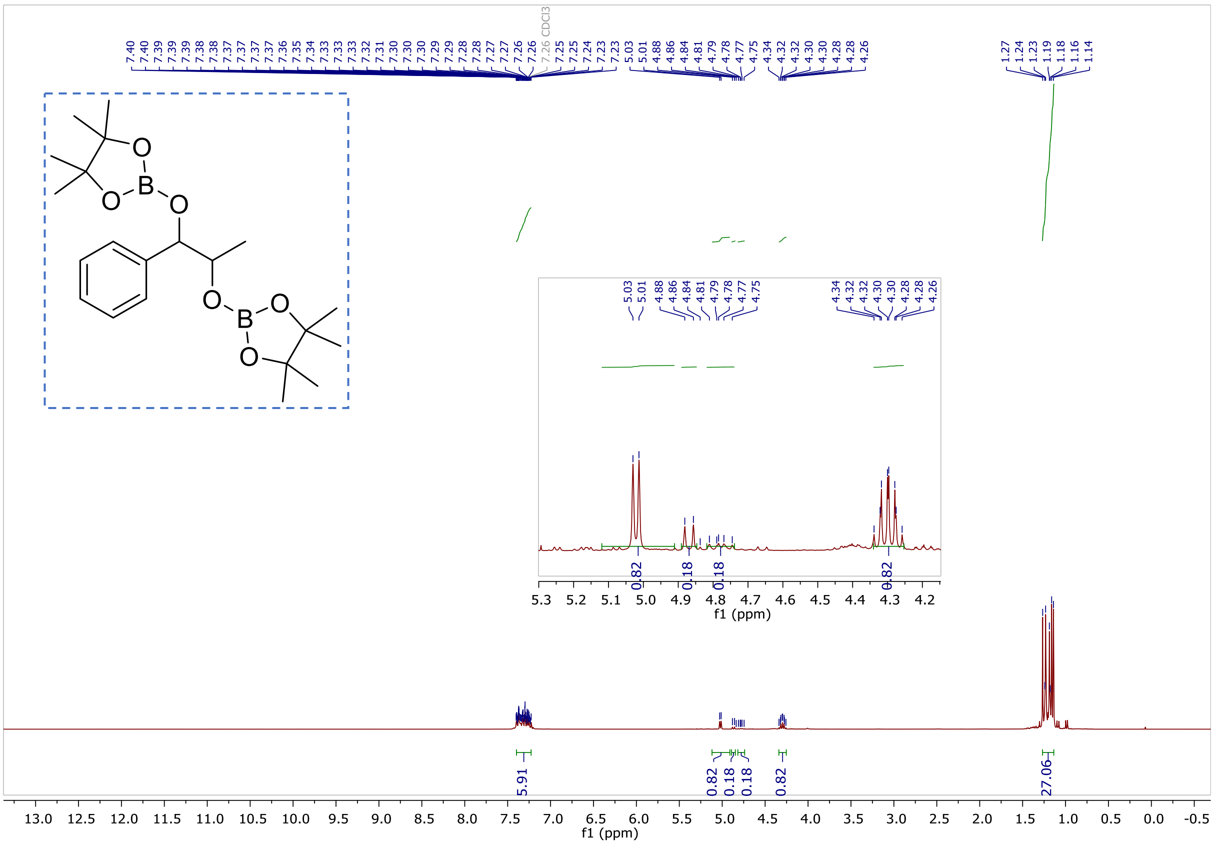

Figure S19 – ^{1}H NMR spectrum of **3o.**

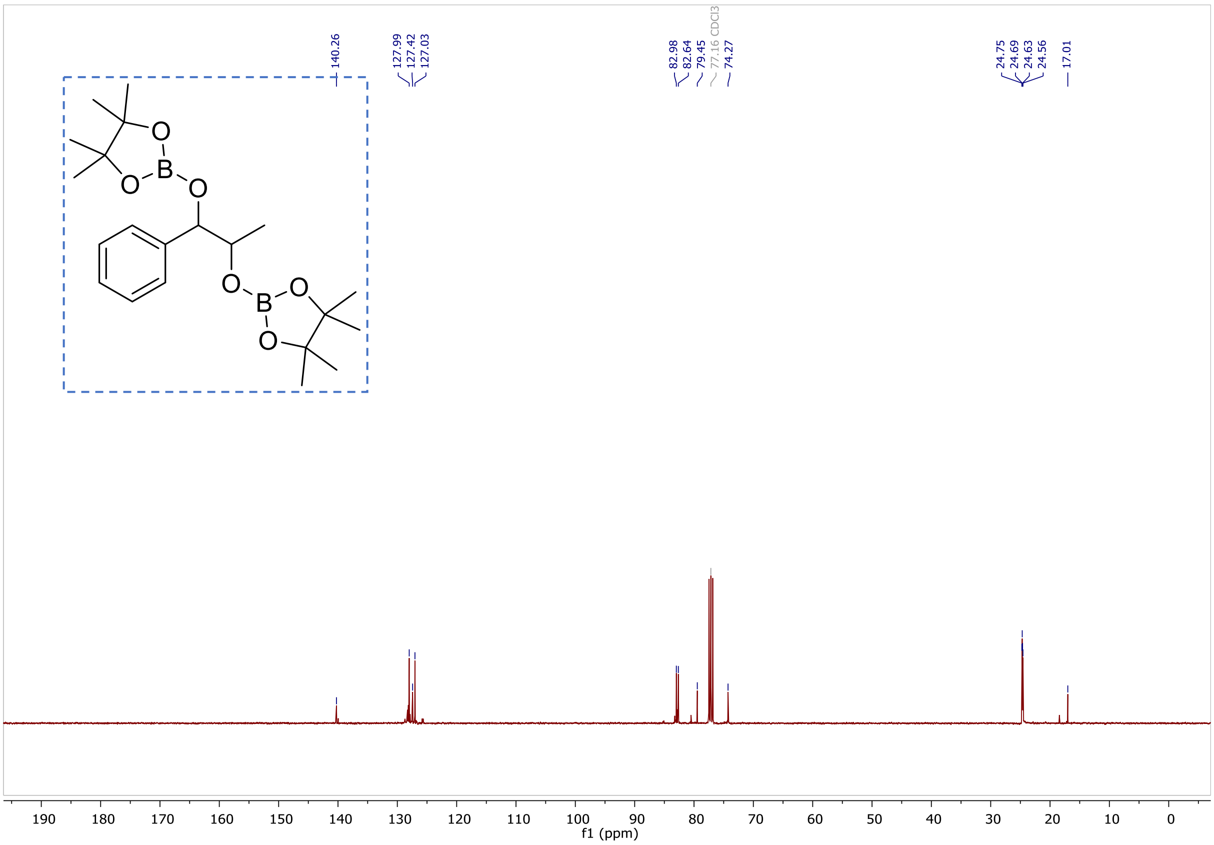

Figure S20 – ^{13}C NMR spectrum of **3o.**

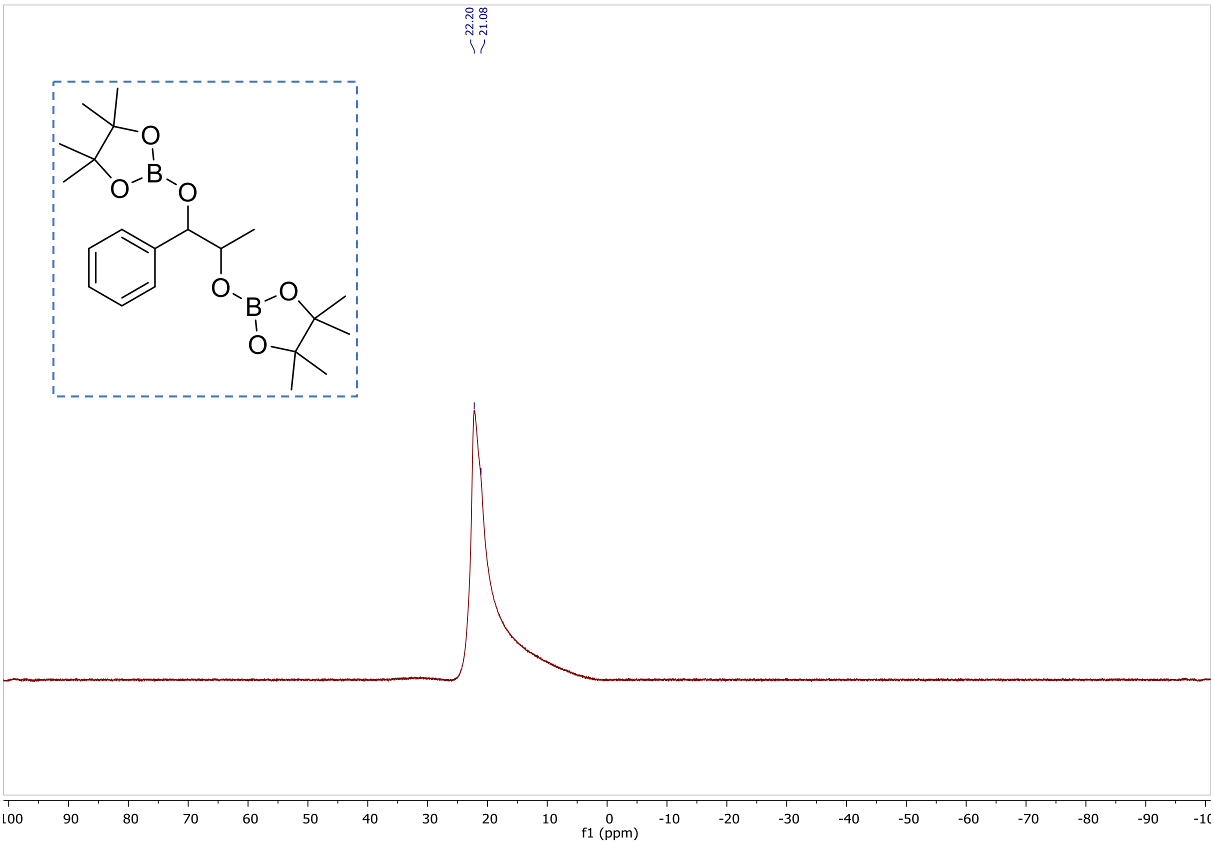

Figure S21 – ^{11}B NMR spectrum of **3o.**

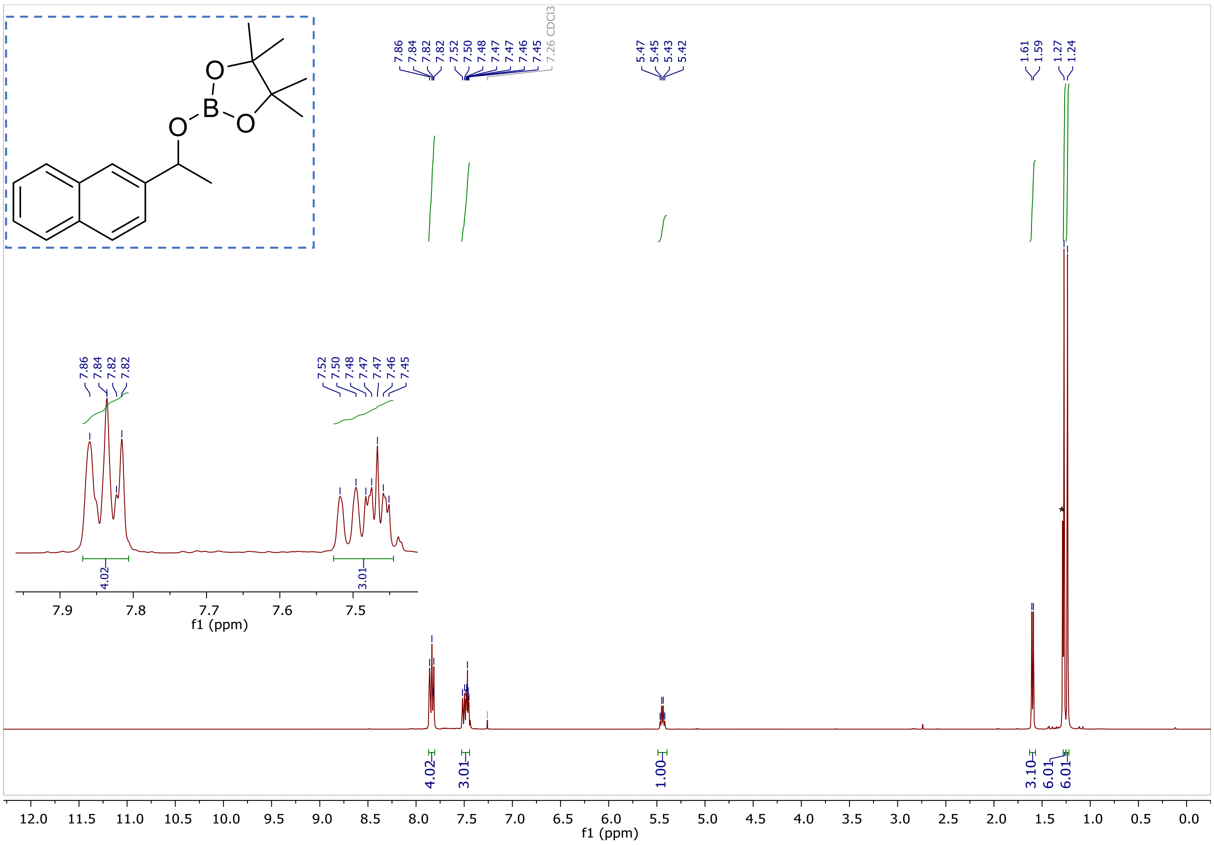

Figure S22 – ^{1}H NMR spectrum of **3p** from crude reaction mixture. *Excess of the HBpin.

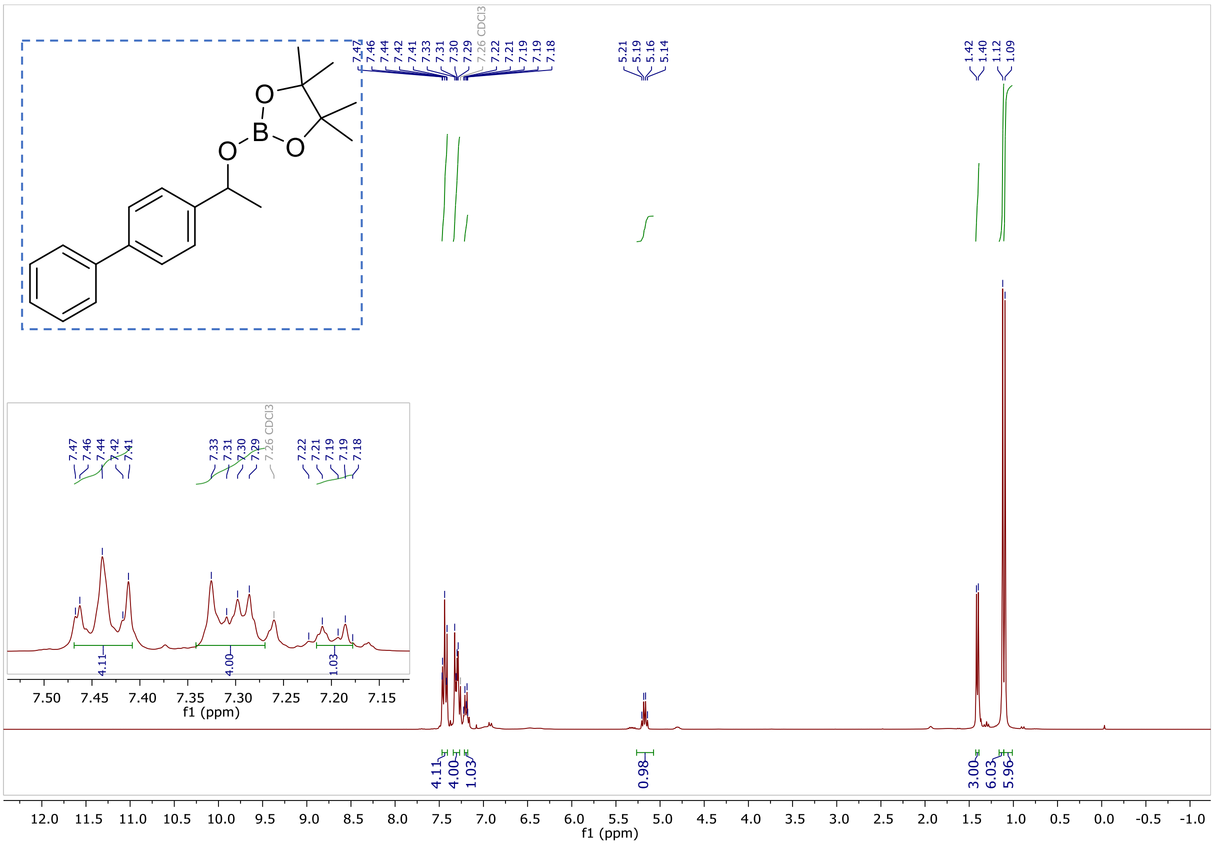

Figure S23 – ^{1}H NMR spectrum of **3q**.

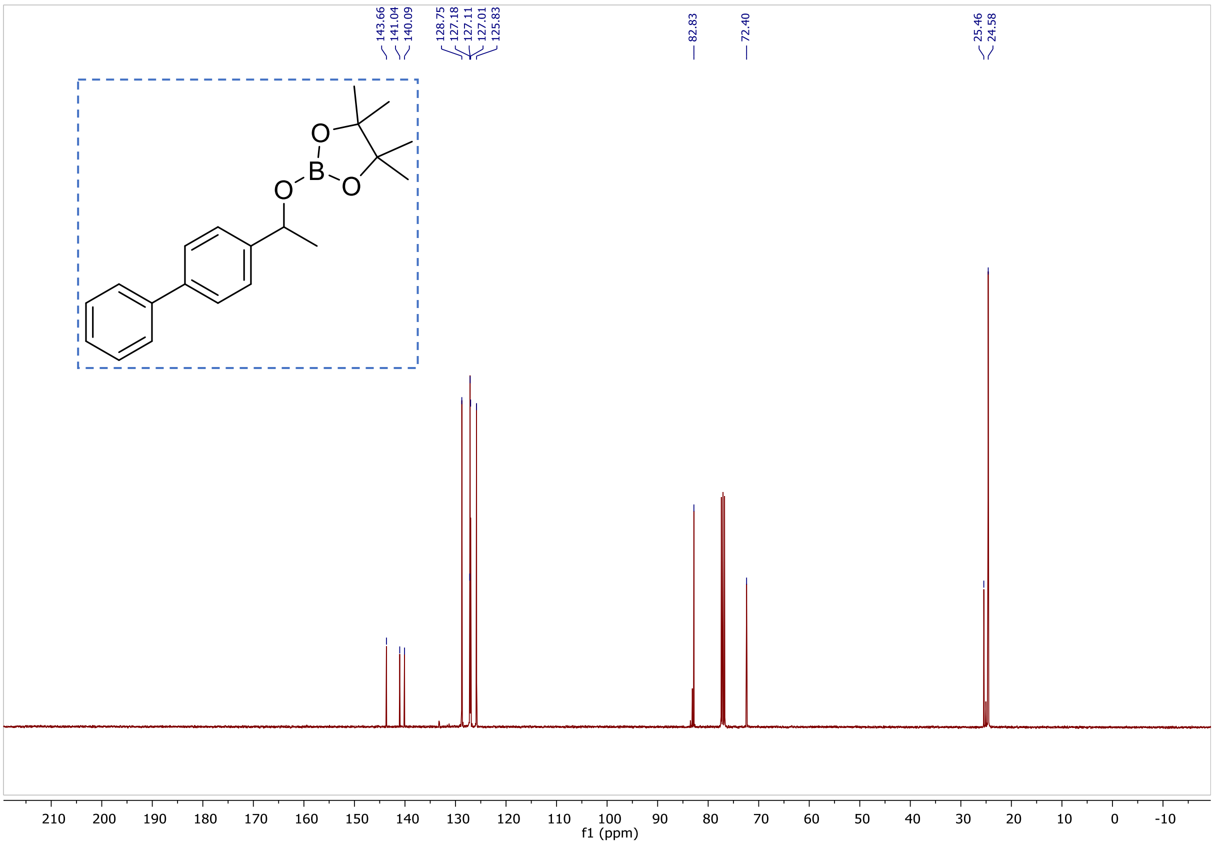

Figure S24 – ^{13}C NMR spectrum of **3q**.

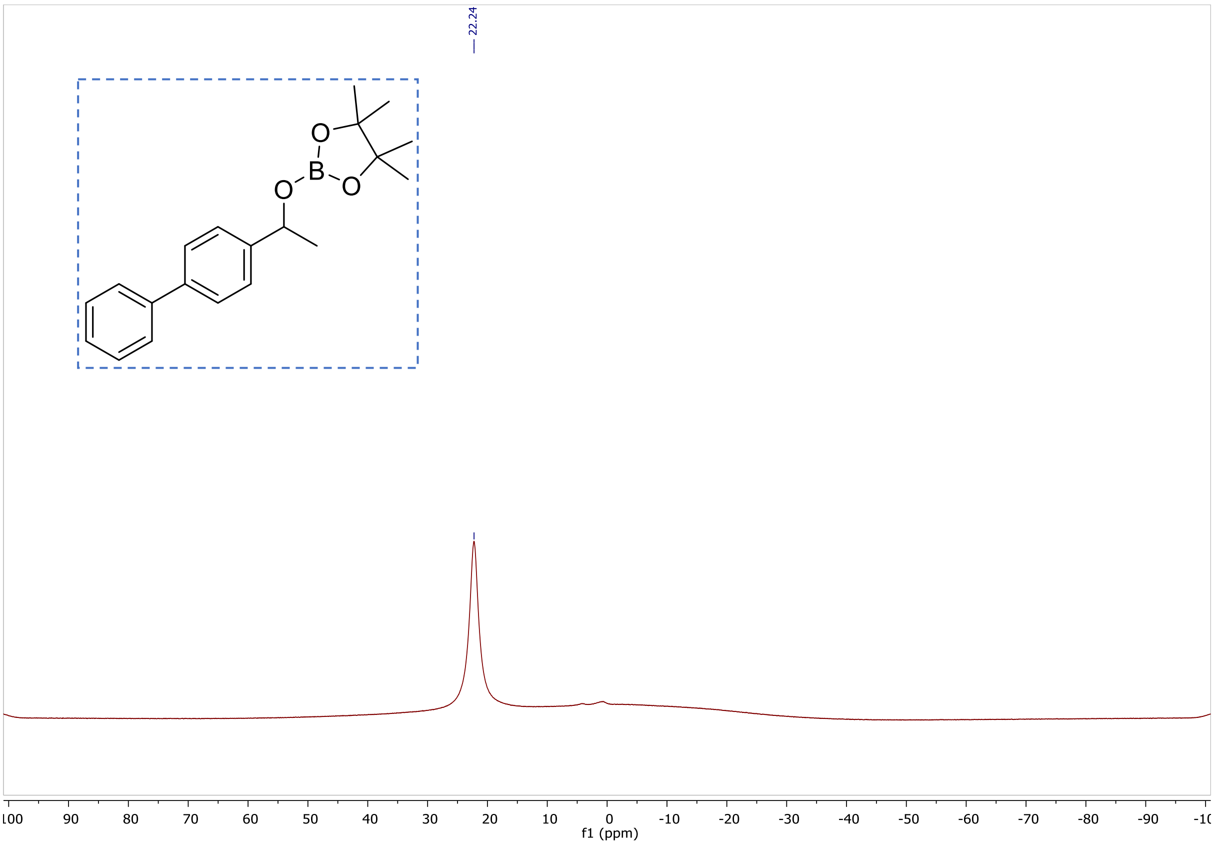

Figure S25 – ^{11}B NMR spectrum of **3q**.

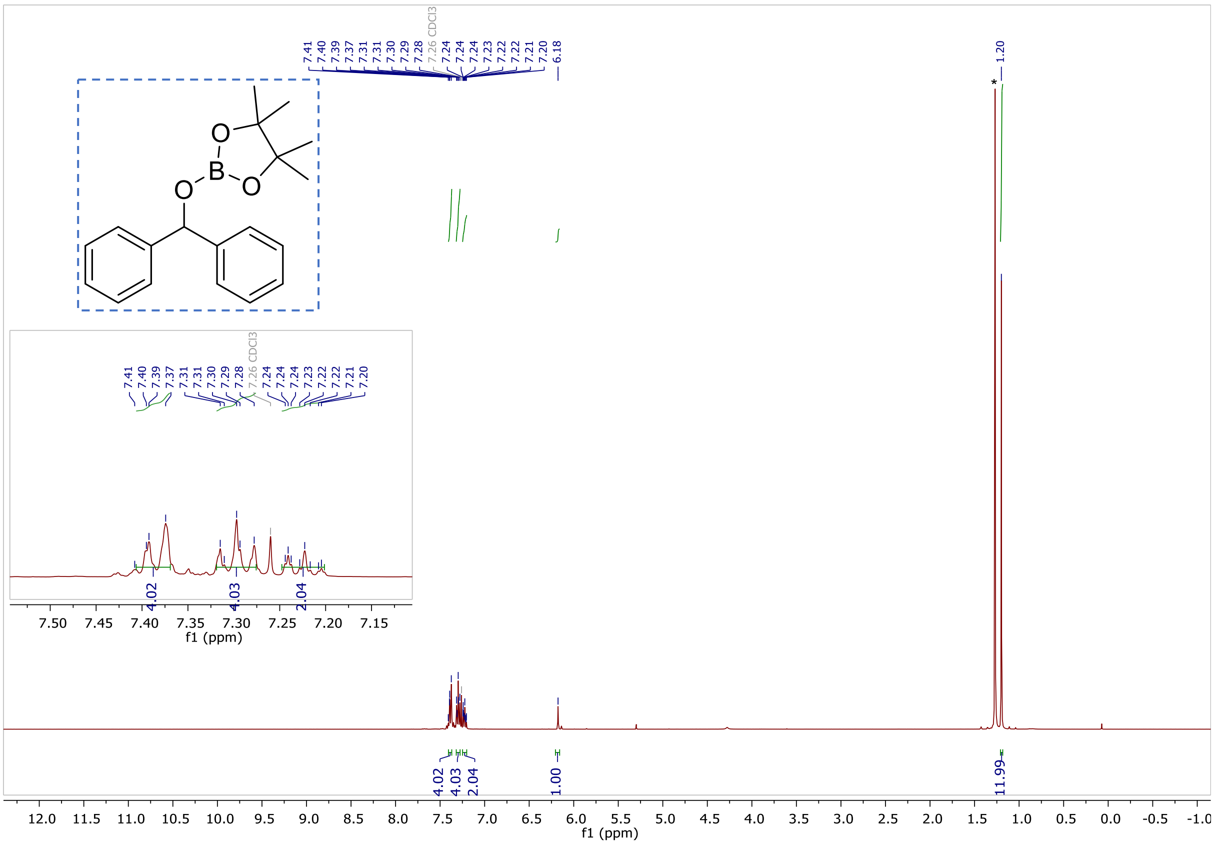

Figure S26 – ^{1}H NMR spectrum of **3r** from crude reaction mixture. *Excess of the HBpin.

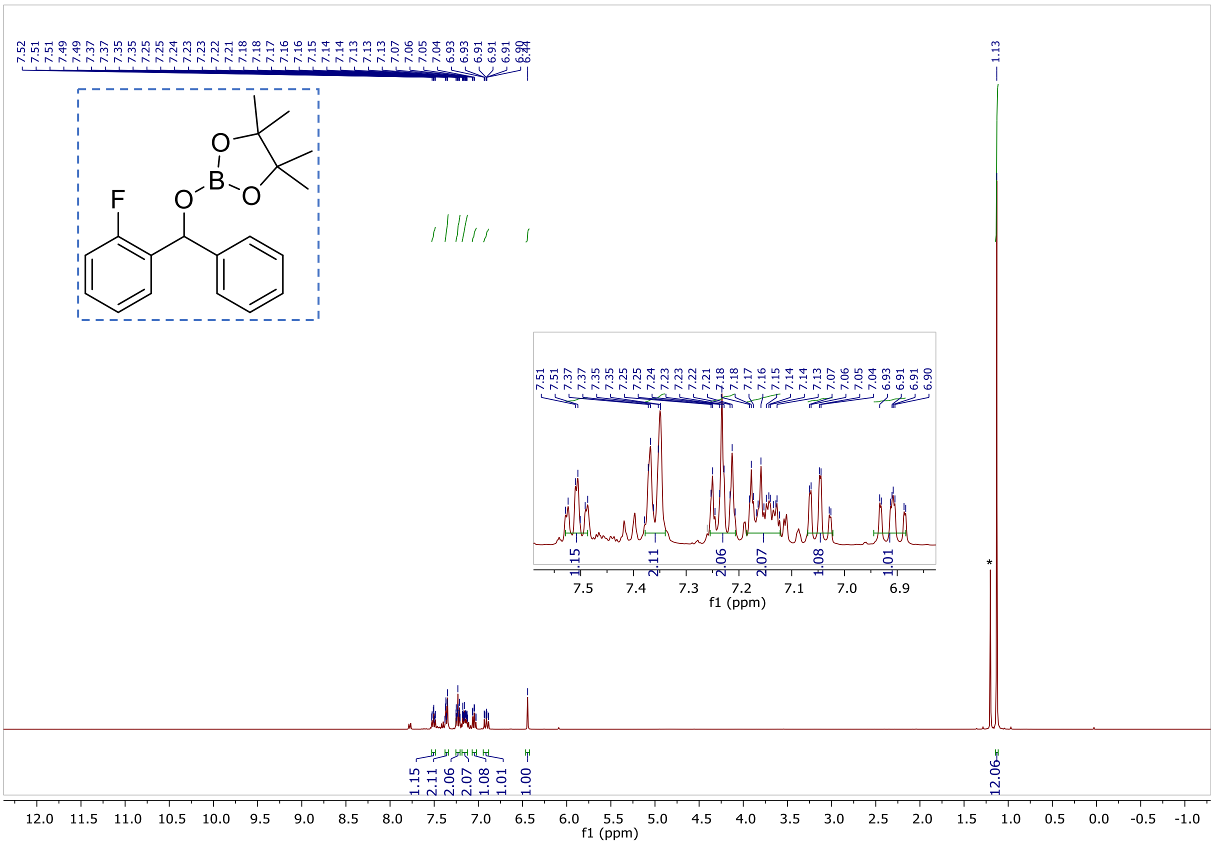

Figure S27 – ^{1}H NMR spectrum of **3s** from crude reaction mixture. *Excess of the HBpin.

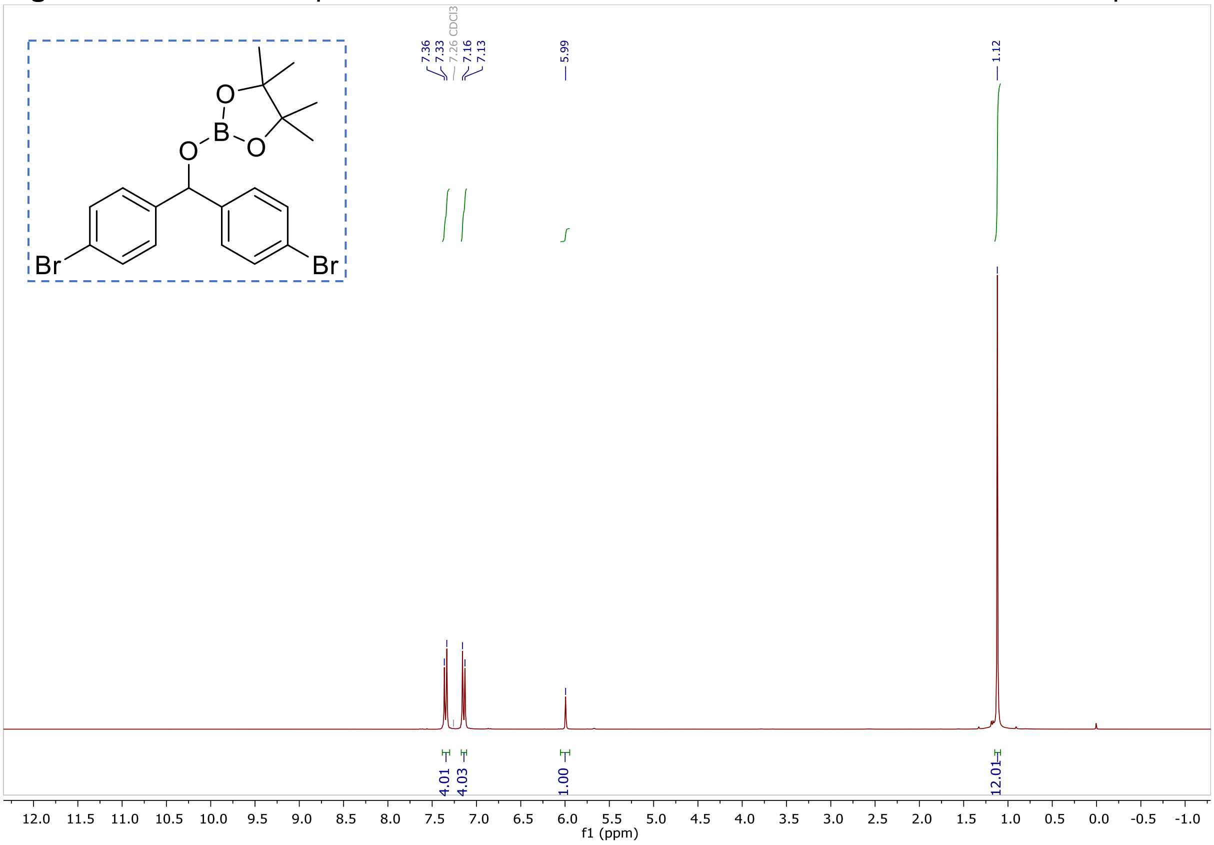

Figure S28 – ^{1}H NMR spectrum of **3t**.

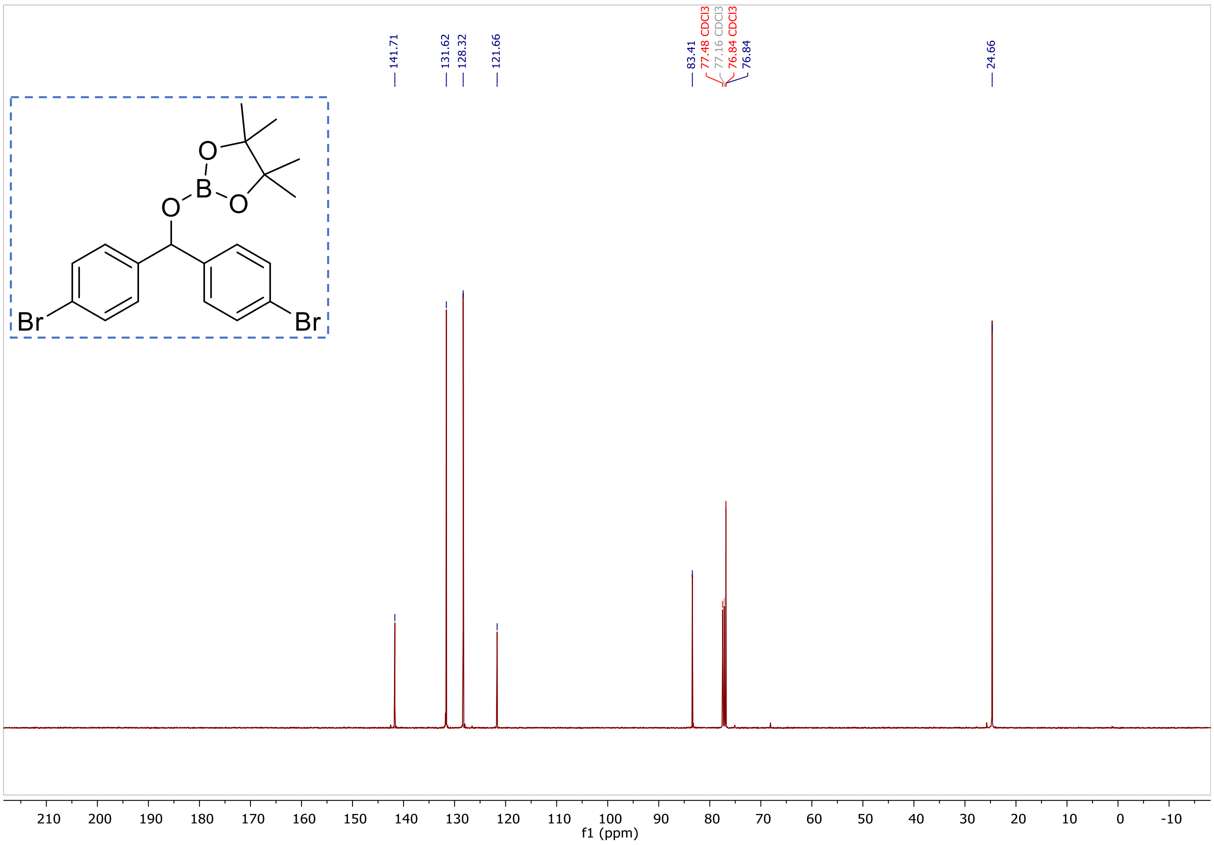

Figure S29 – ^{13}C NMR spectrum of **3t**.

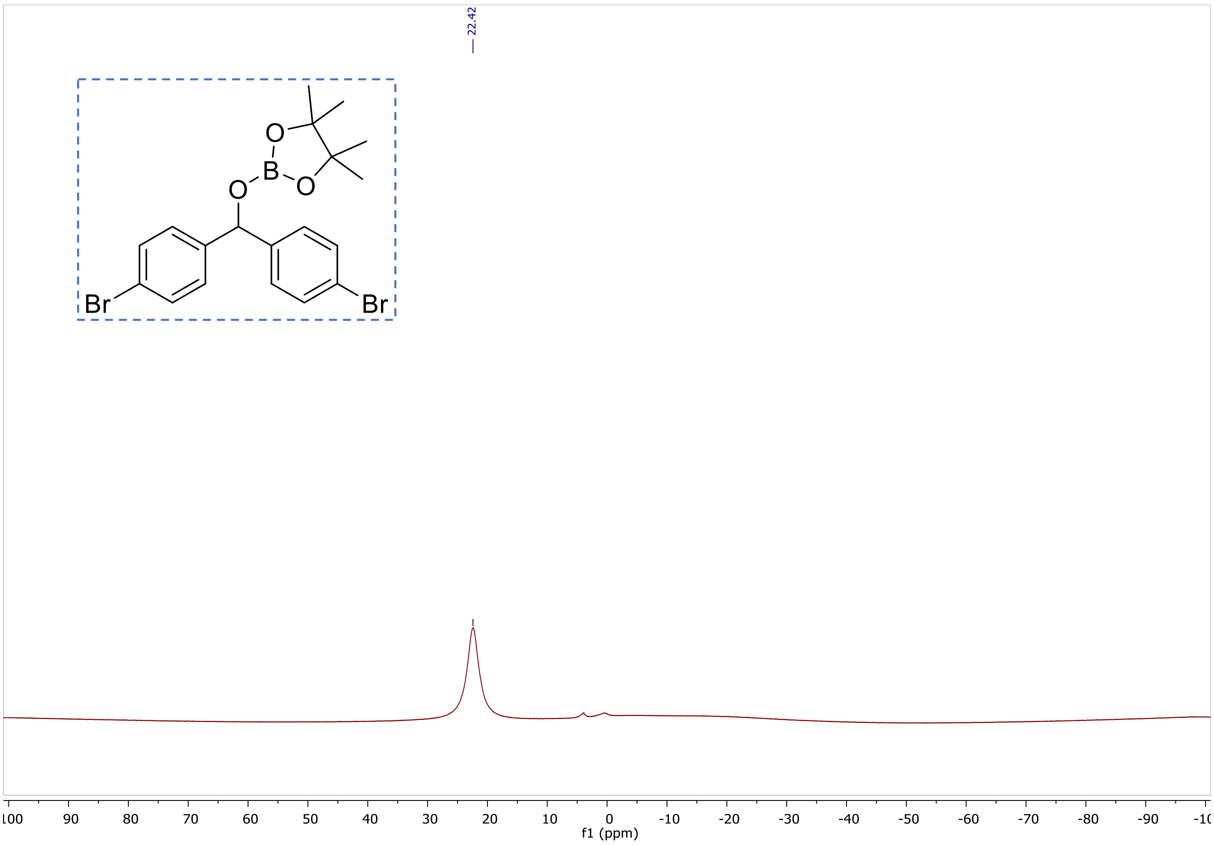

Figure S30 – ^{11}B NMR spectrum of **3t**.

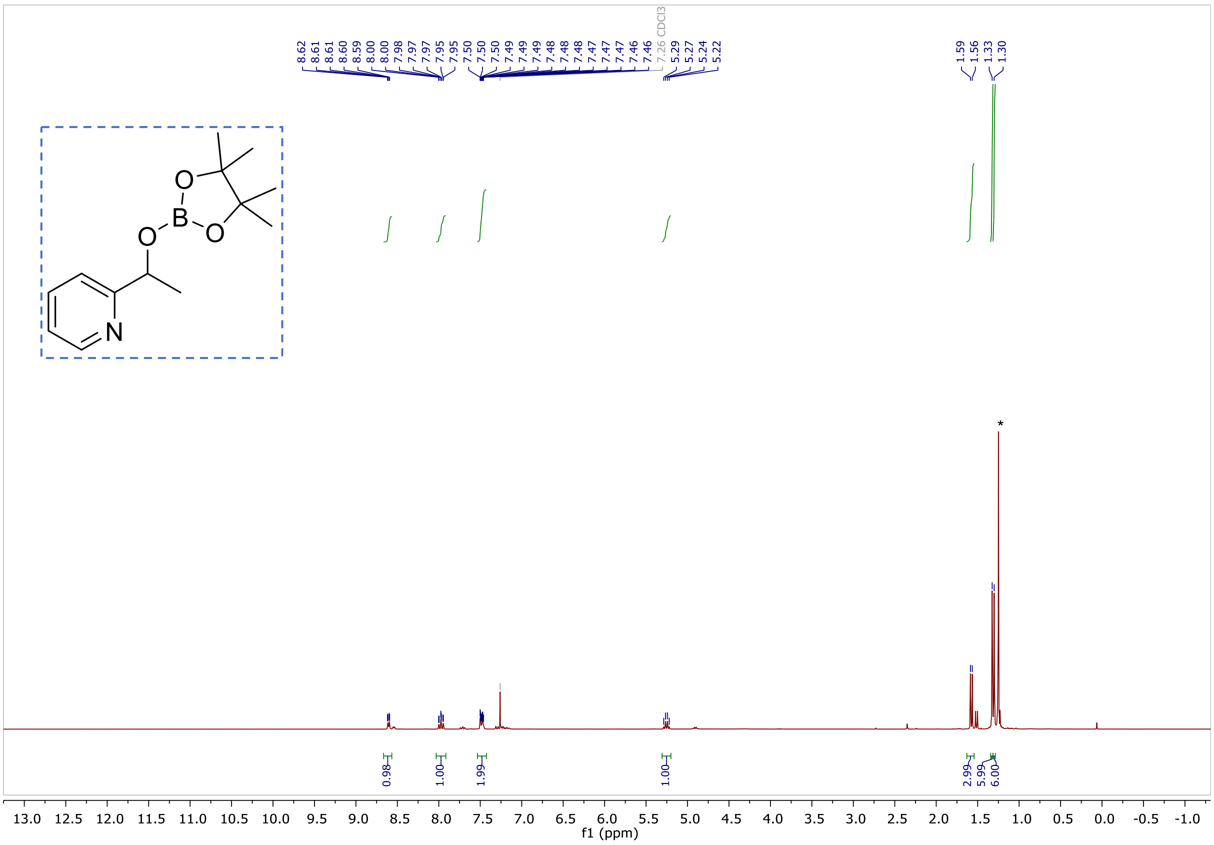

Figure S31 – ^{1}H NMR spectrum of **3u** from crude reaction mixture. *Excess of the HBpin.

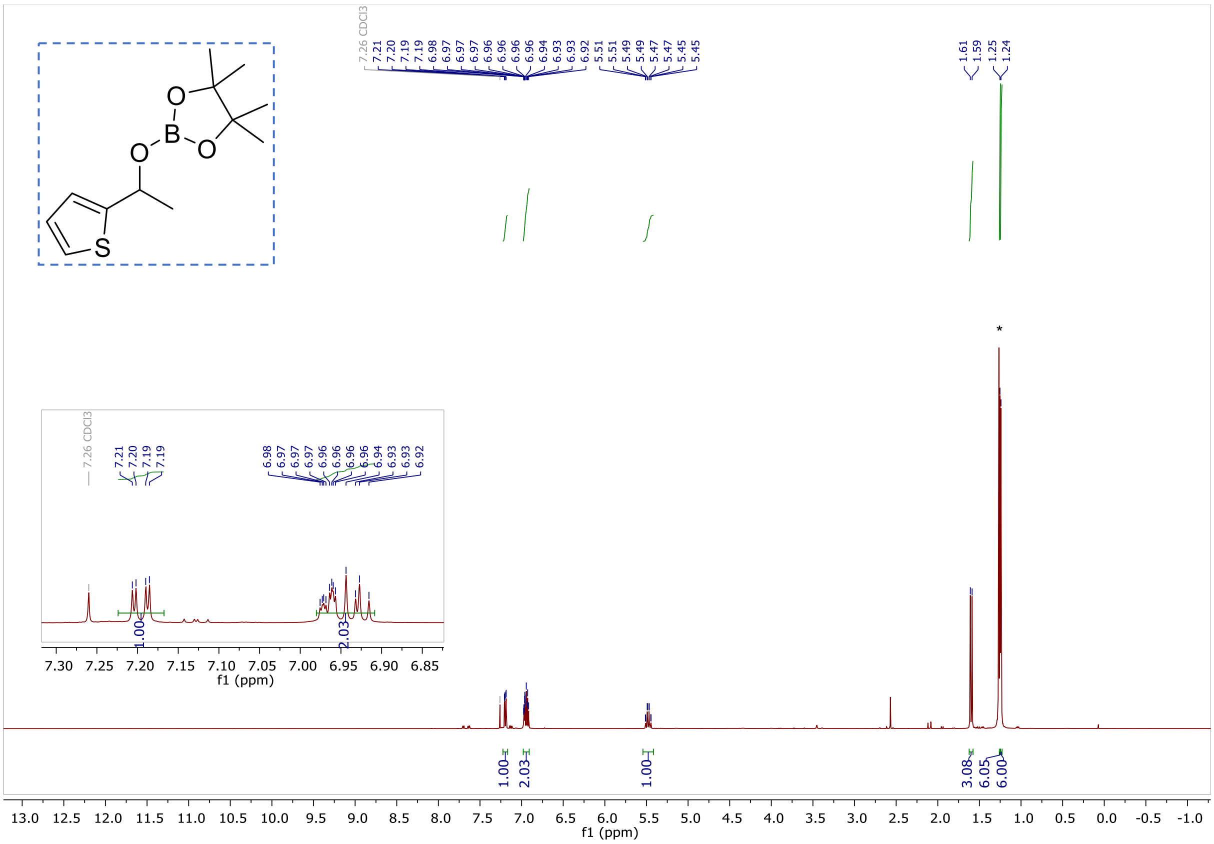

Figure S32 – ^{1}H NMR spectrum of **3v** from crude reaction mixture. *Excess of the HBpin.

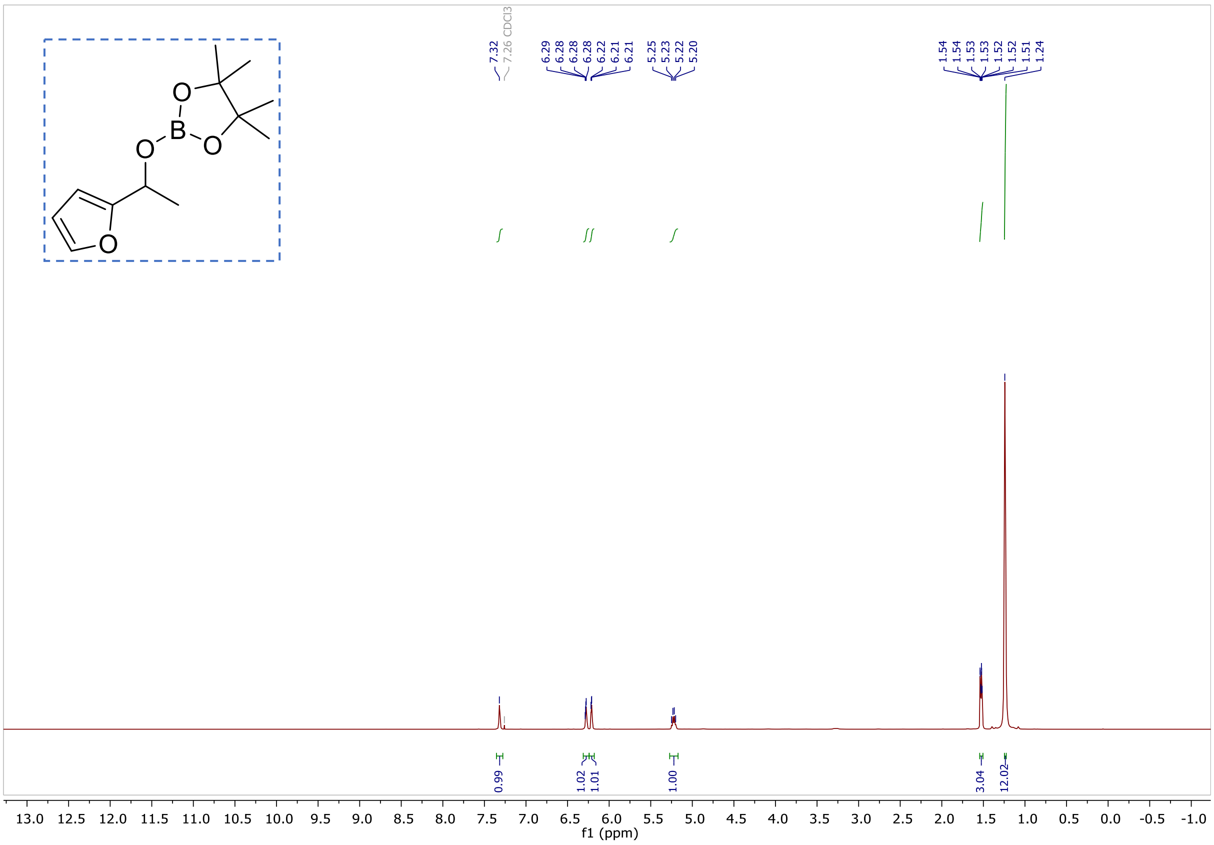

Figure S33 – ^{1}H NMR spectrum of **3w**.

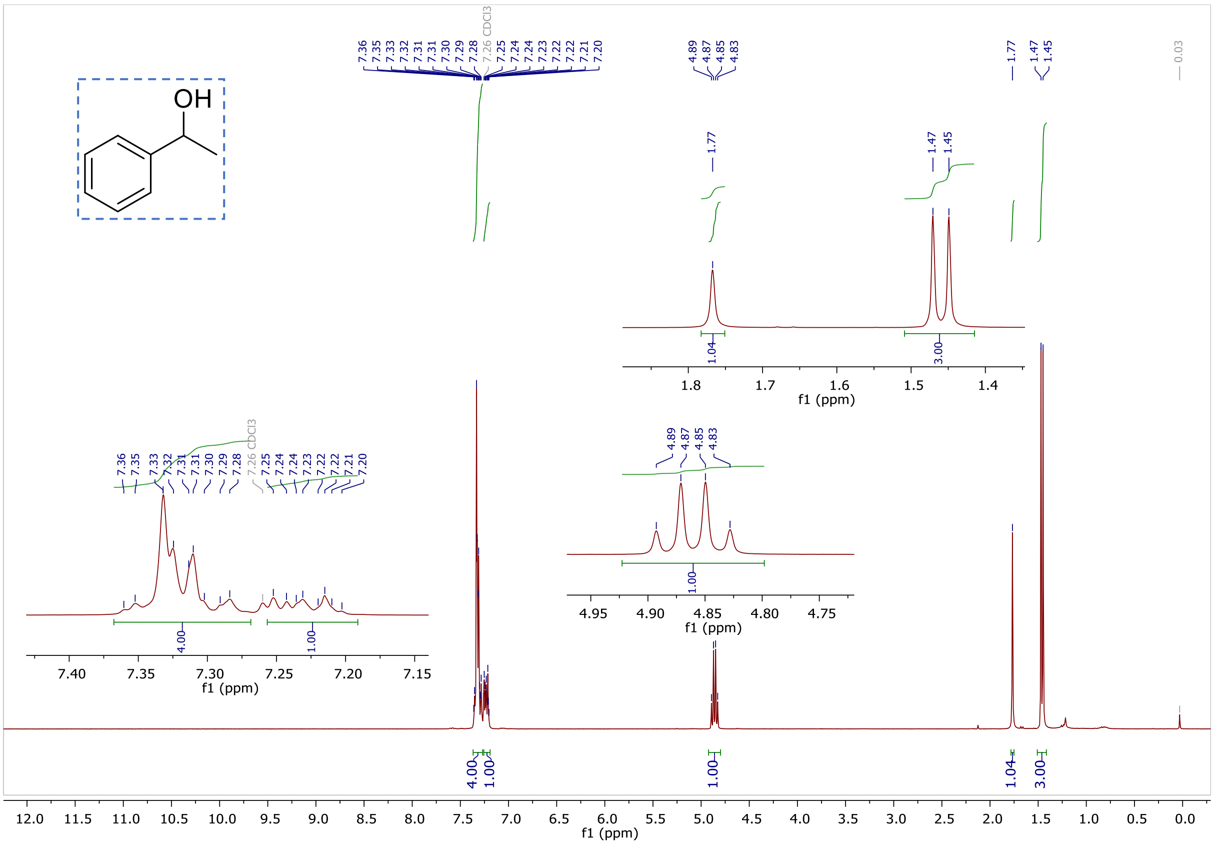

Figure S34 – ^{1}H NMR spectrum of **5a**.

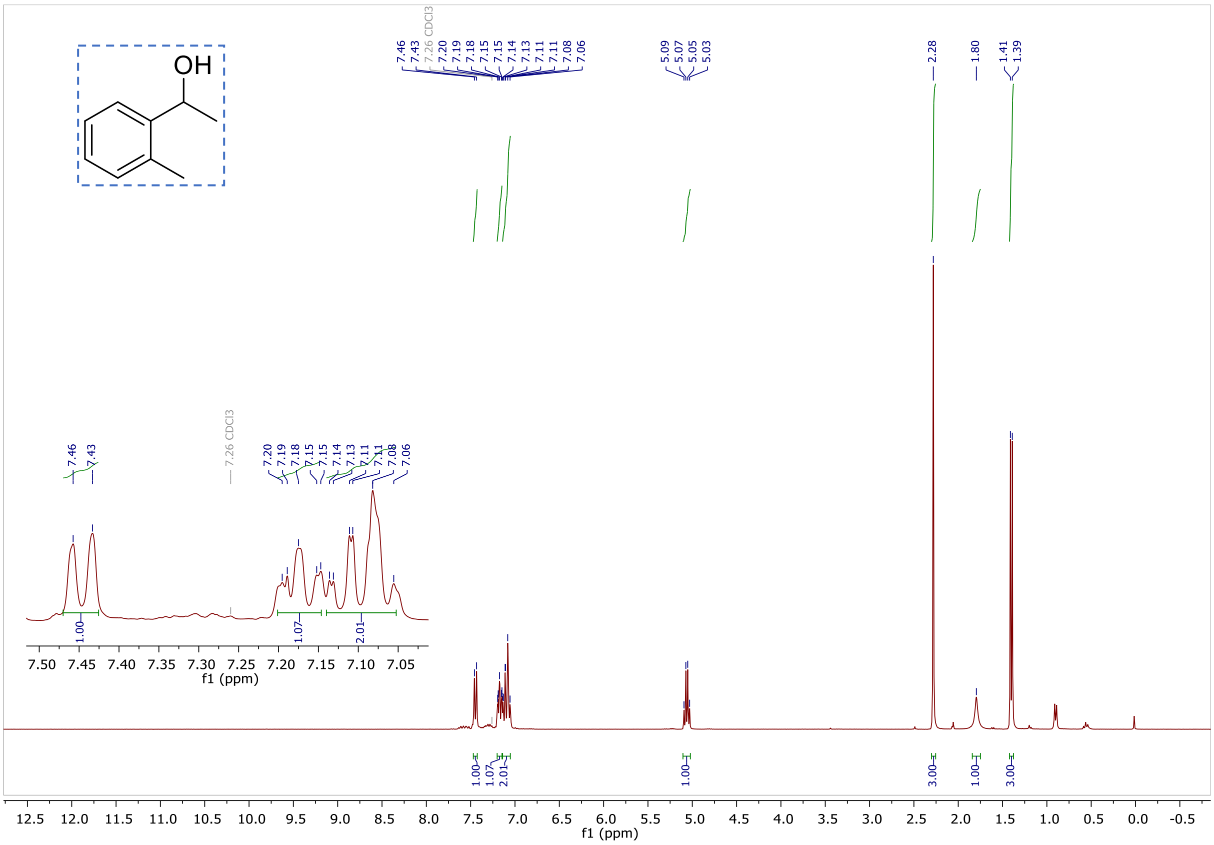

Figure S35 – ^{1}H NMR spectrum of **5b**.

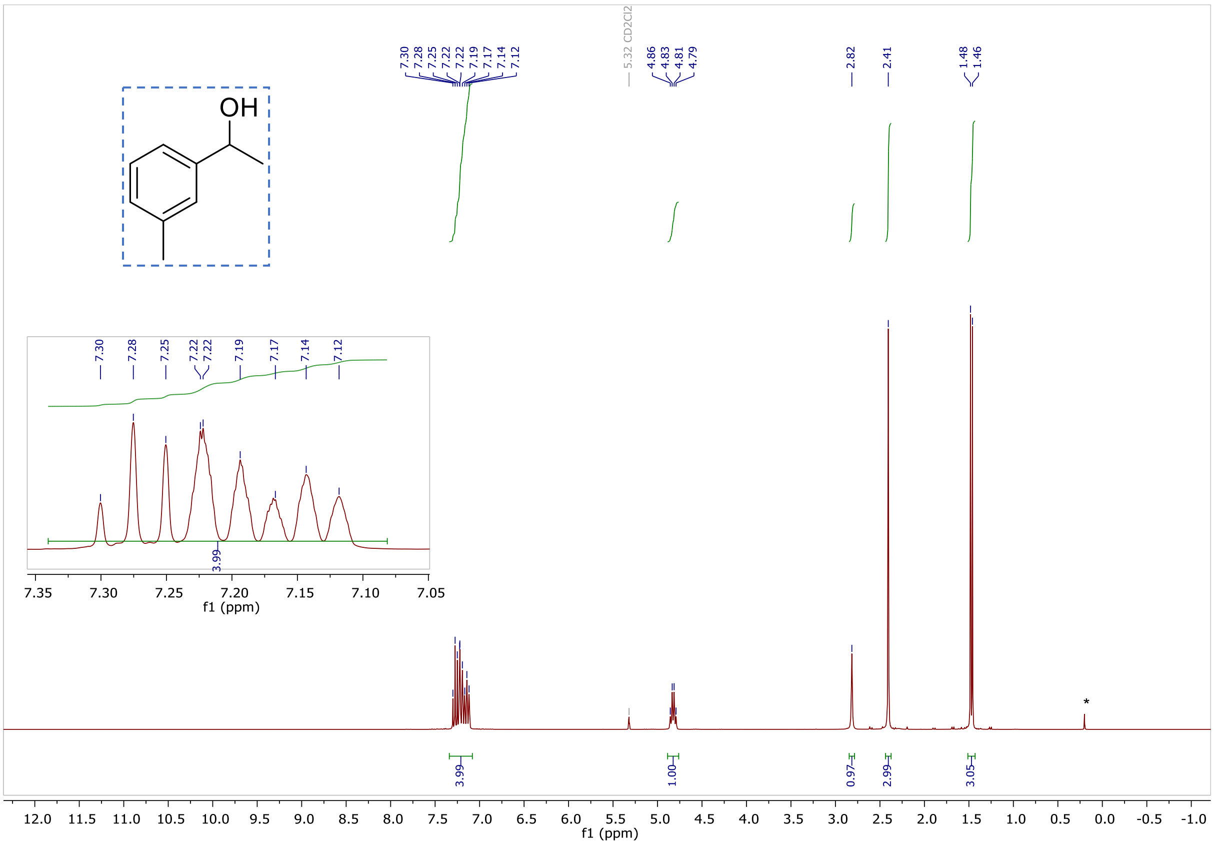

Figure S36 – ^{1}H NMR spectrum of **5c**.* - Grease.

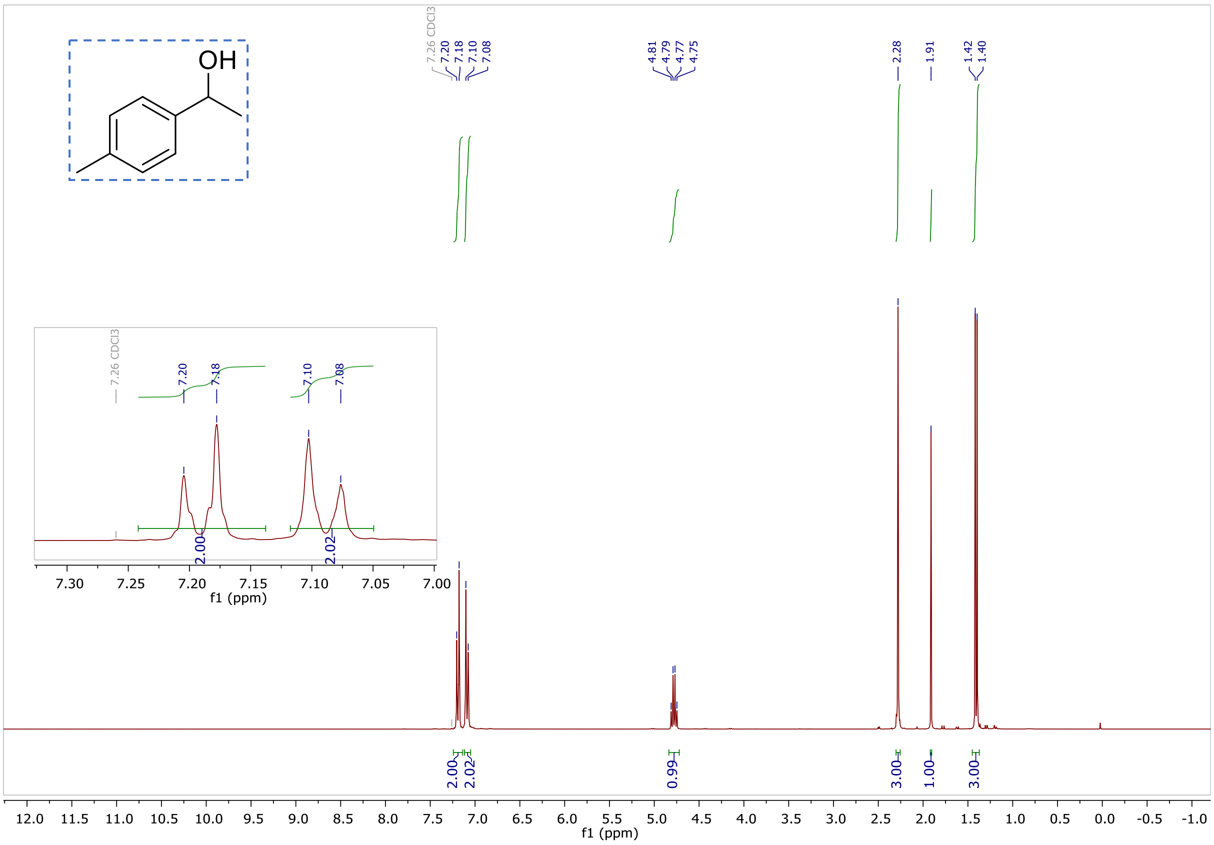

Figure S37 – ^{1}H NMR spectrum of **5d**.

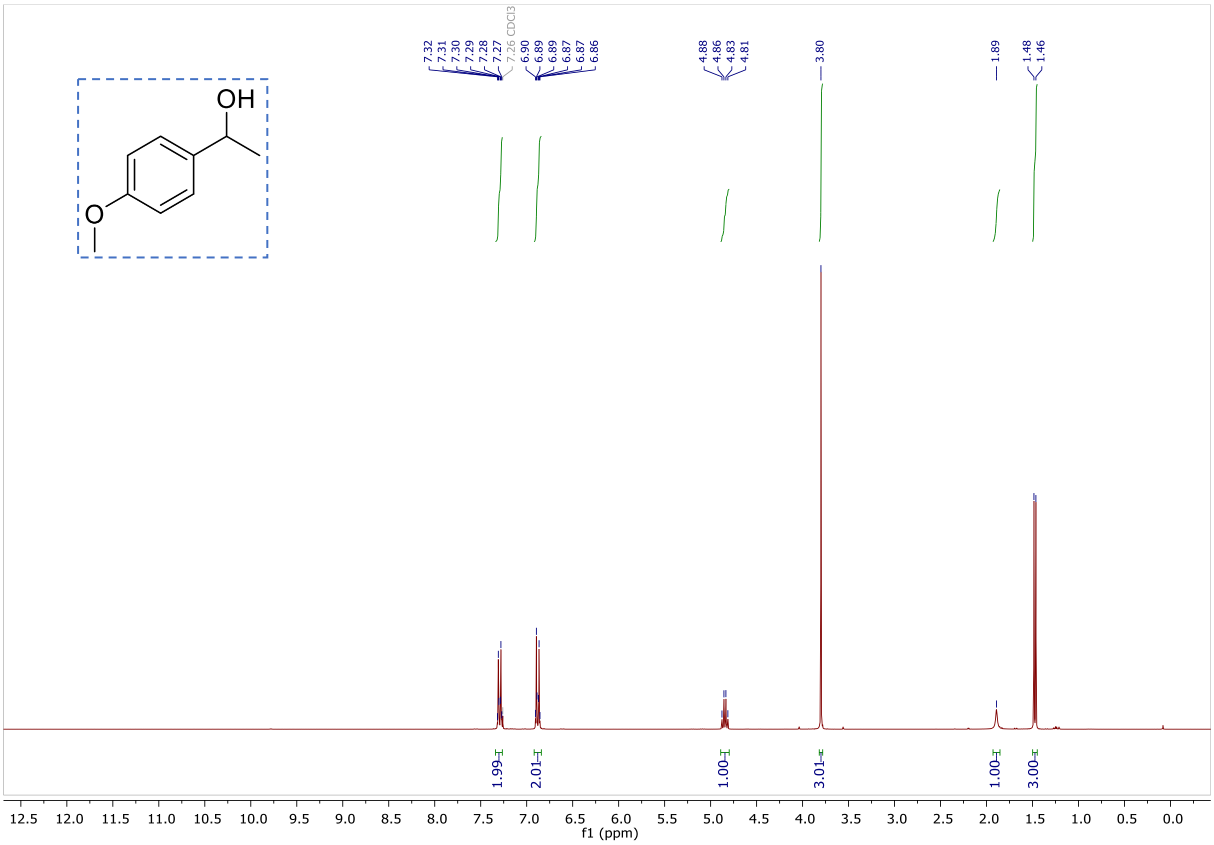

Figure S38 – ^{1}H NMR spectrum of **5e**.

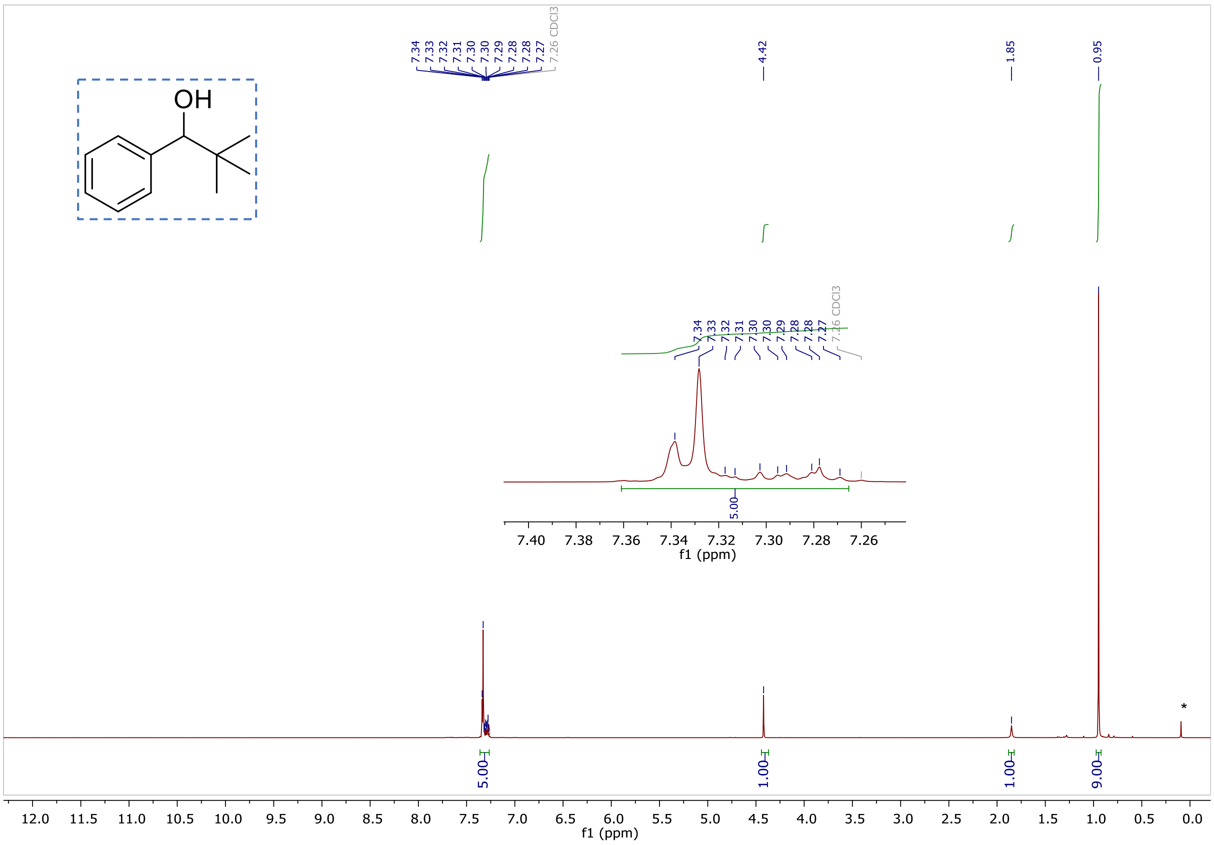

Figure S39 – ^{1}H NMR spectrum of **5f**. * - Grease.

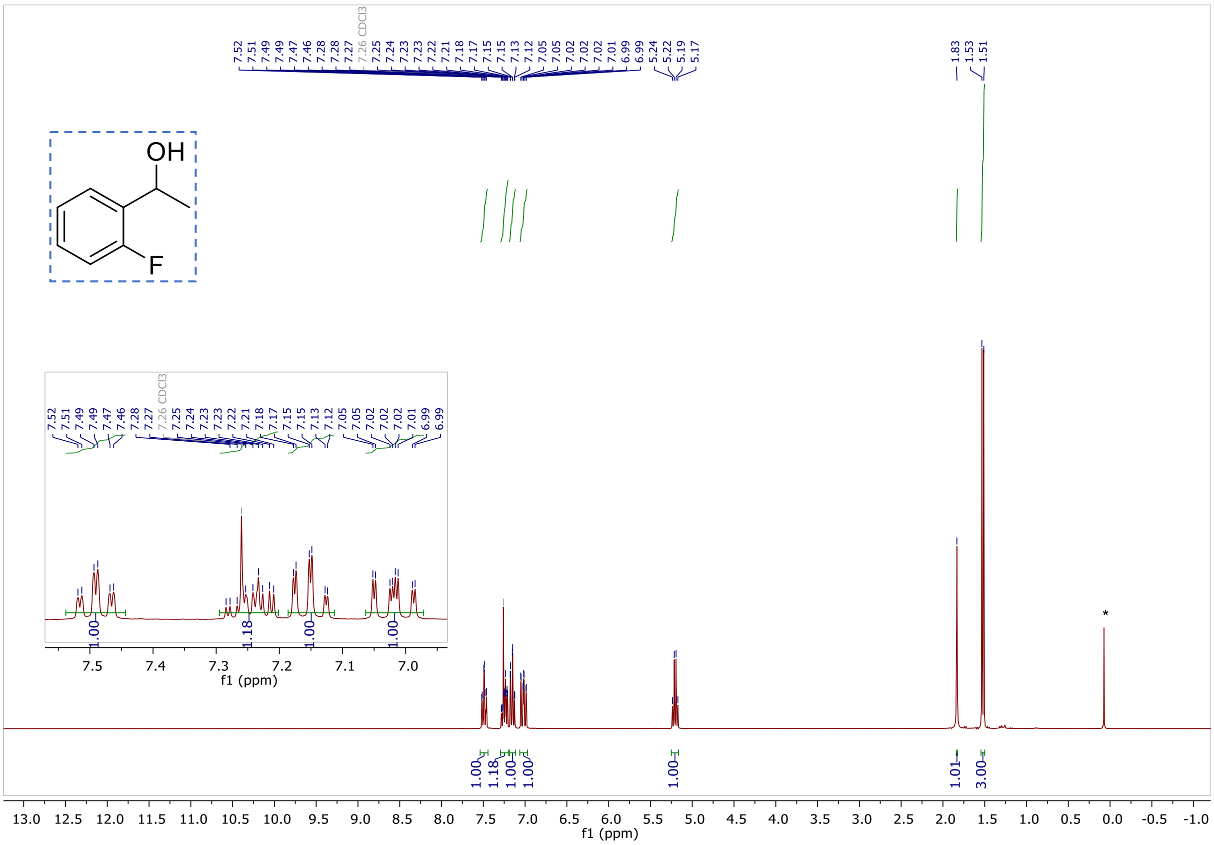

Figure S40 – ^{1}H NMR spectrum of **5g**. * - Grease.

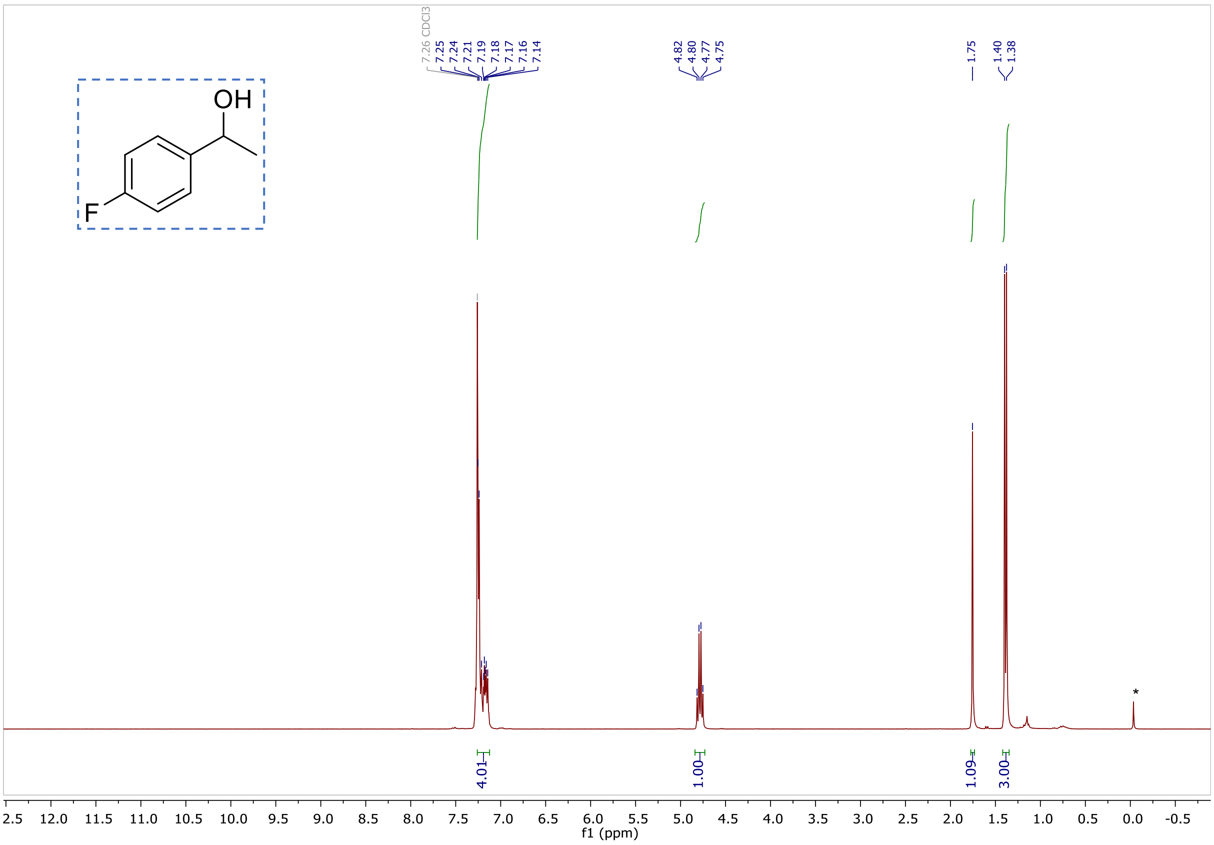

Figure S41 – ^{1}H NMR spectrum of **5h**. * - Grease.

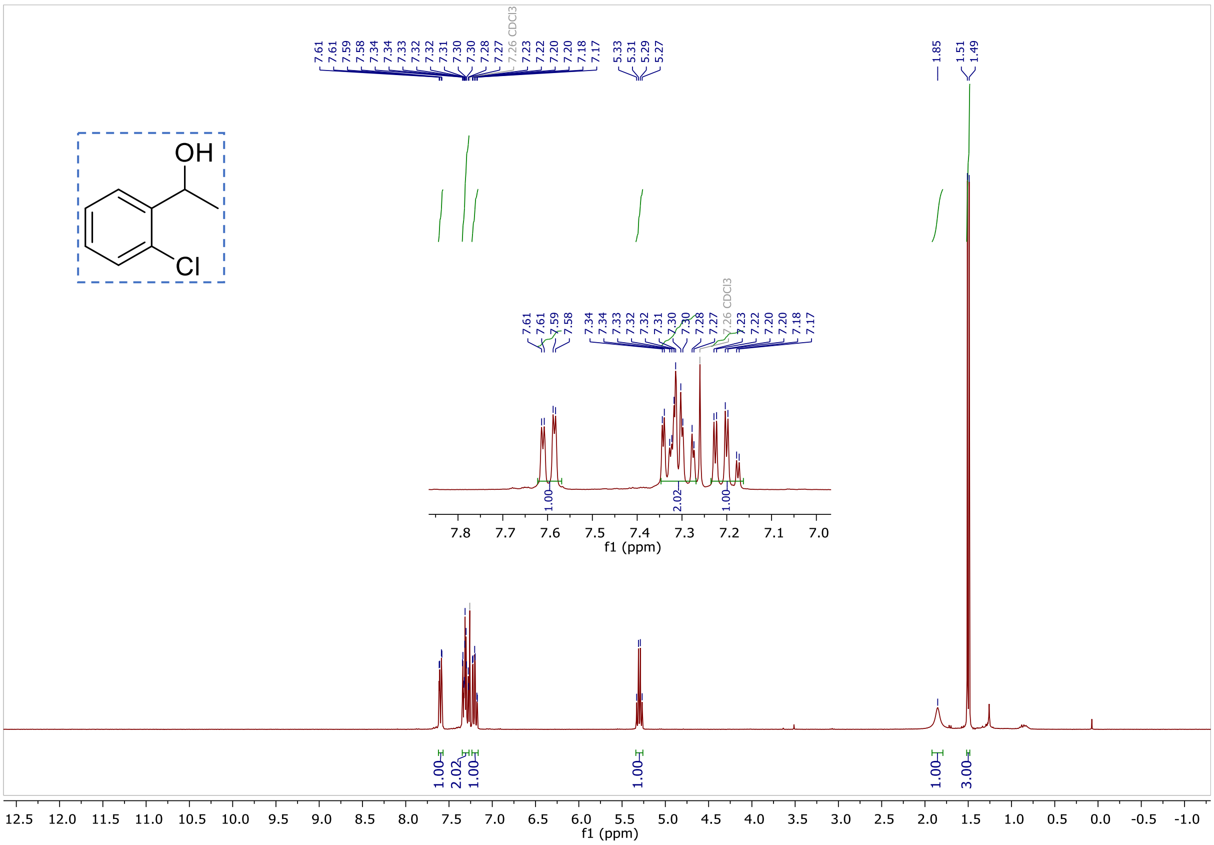

Figure S42 – ^{1}H NMR spectrum of **5i**.

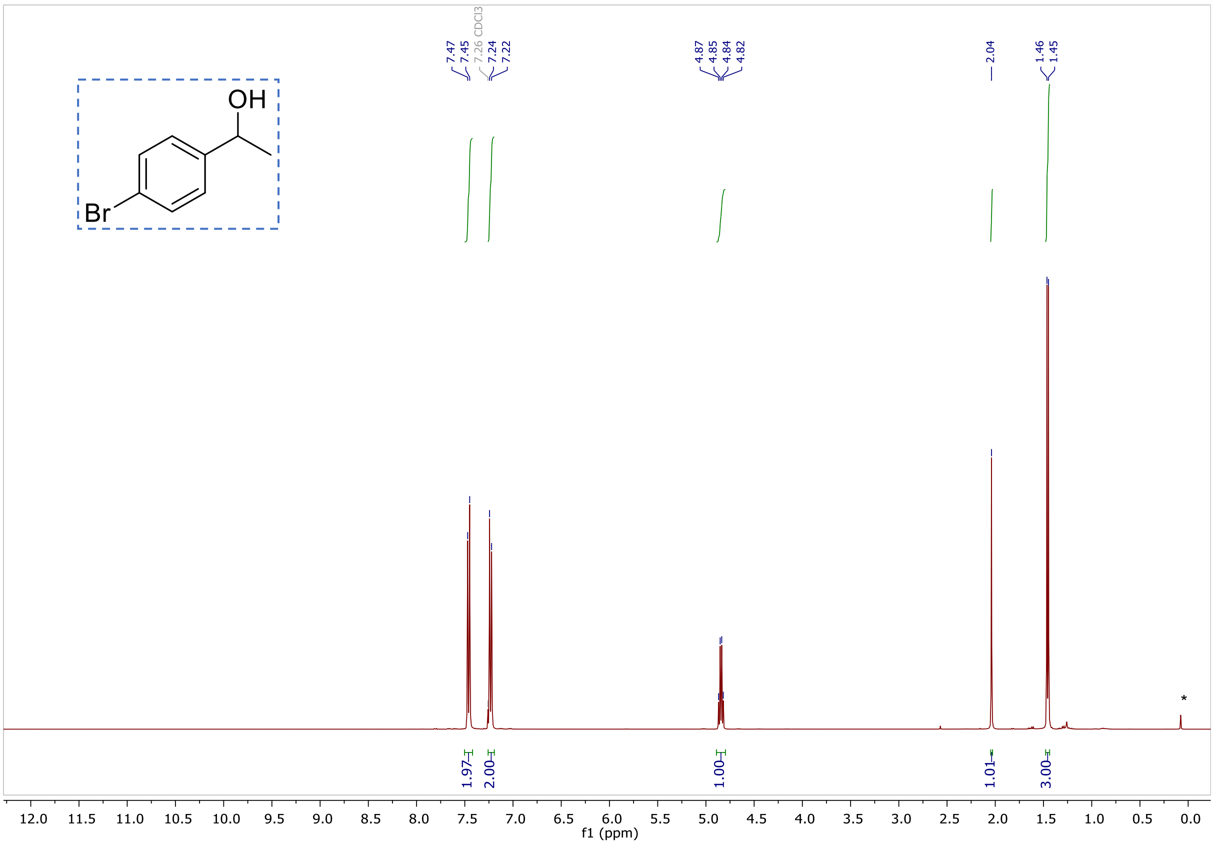

Figure S43 – ^{1}H NMR spectrum of **5j**. * - Grease.

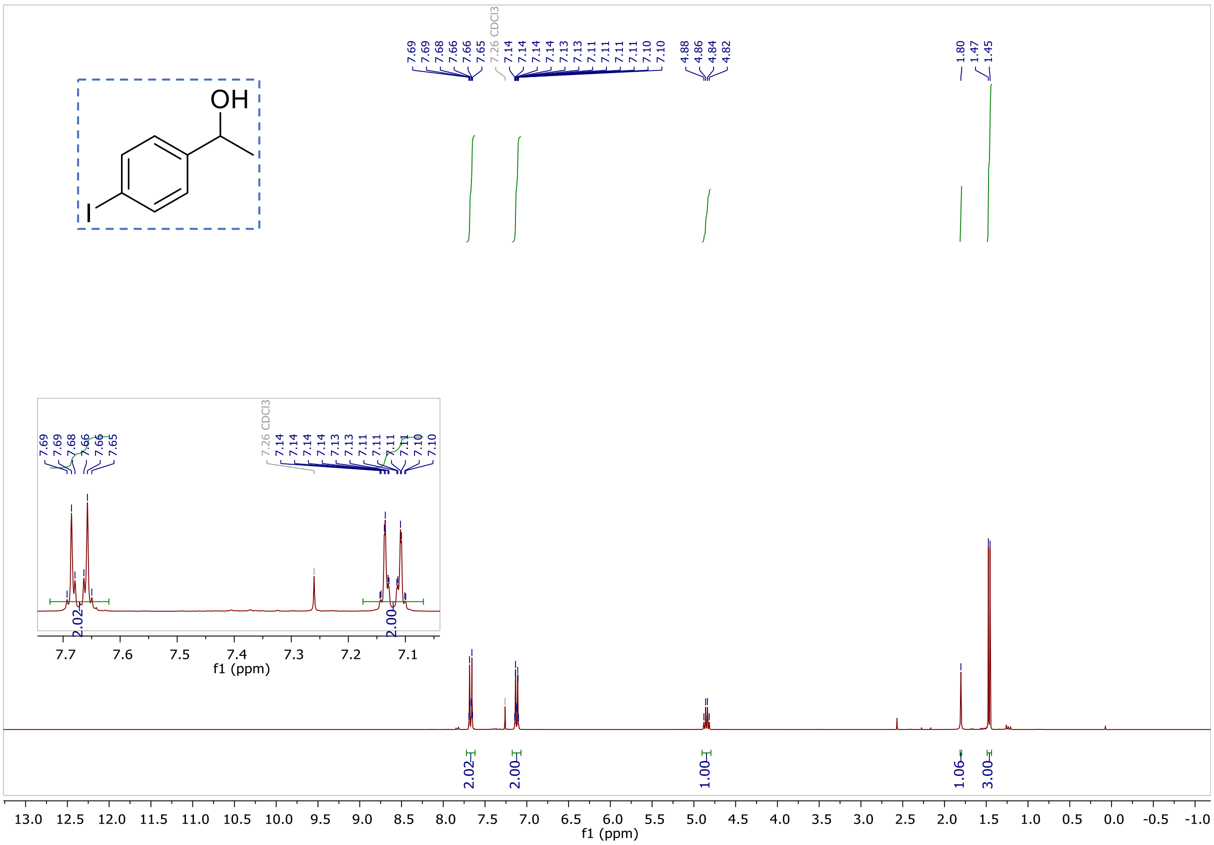

Figure S44 – ^{1}H NMR spectrum of **5k**.

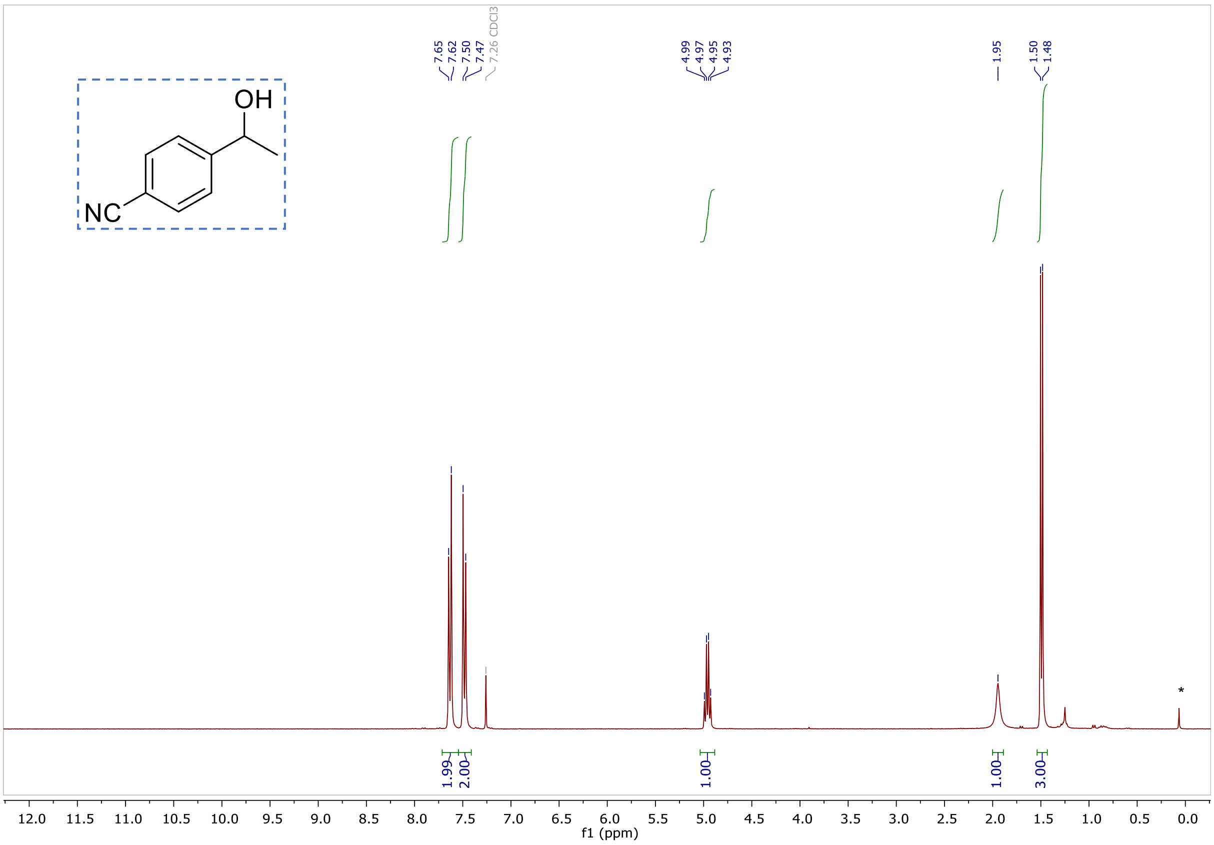

Figure S45 – ^{1}H NMR spectrum of **5l**. * - Grease.

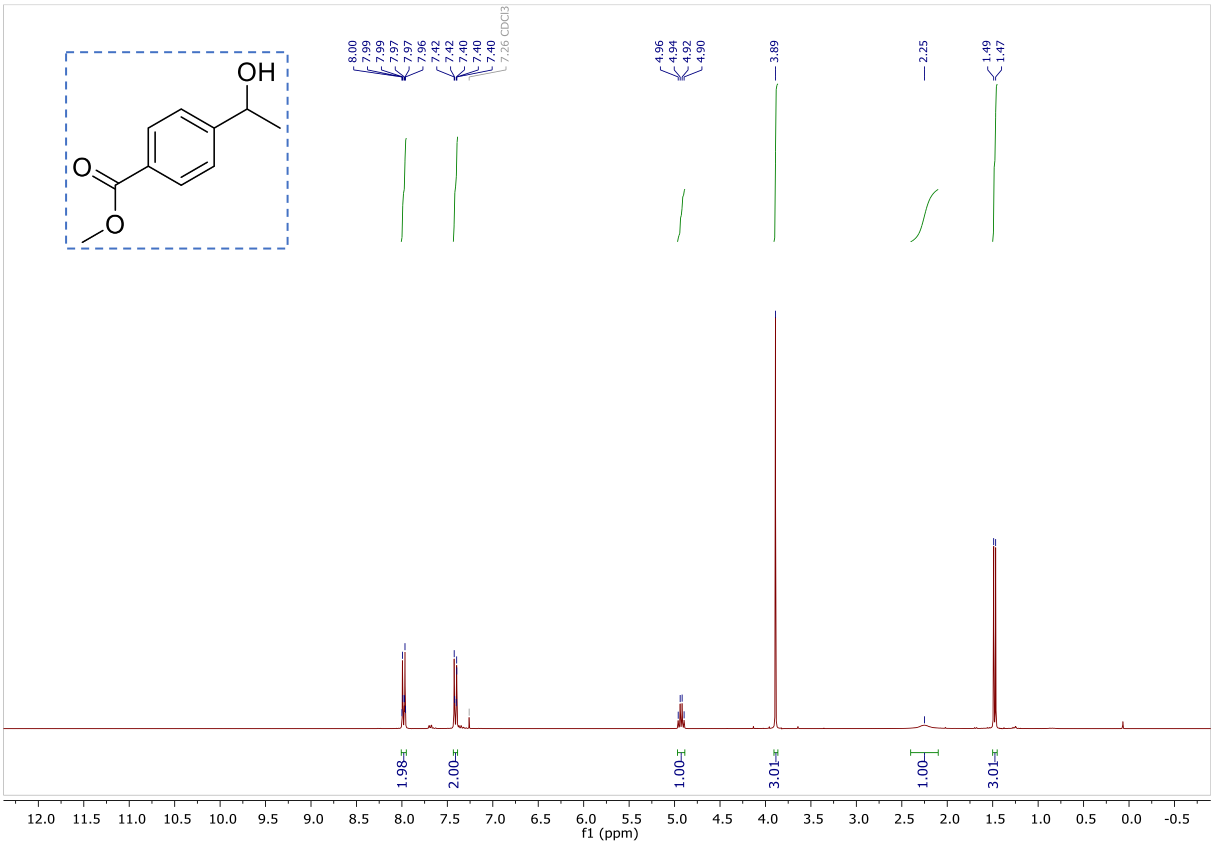

Figure S46 – ^{1}H NMR spectrum of **5m**.

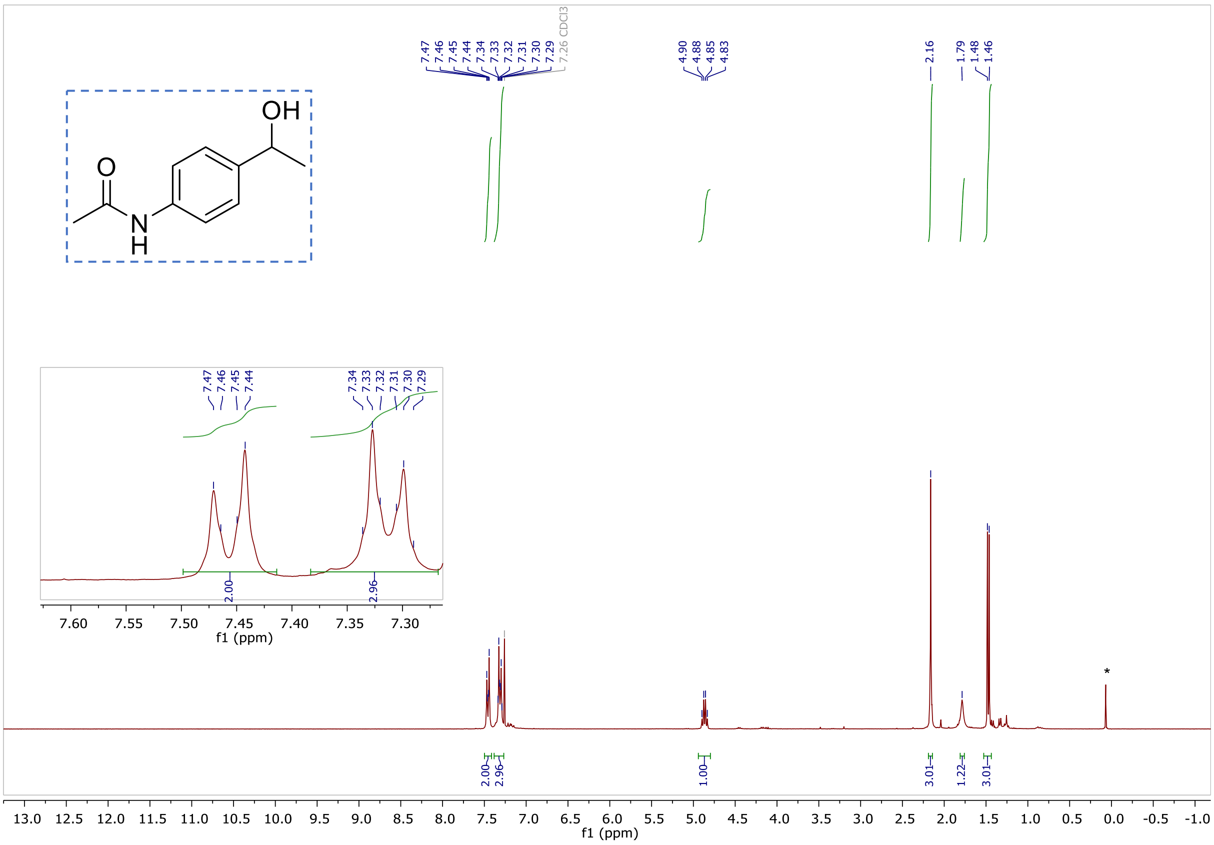

Figure S47 – ^{1}H NMR spectrum of **5n**.

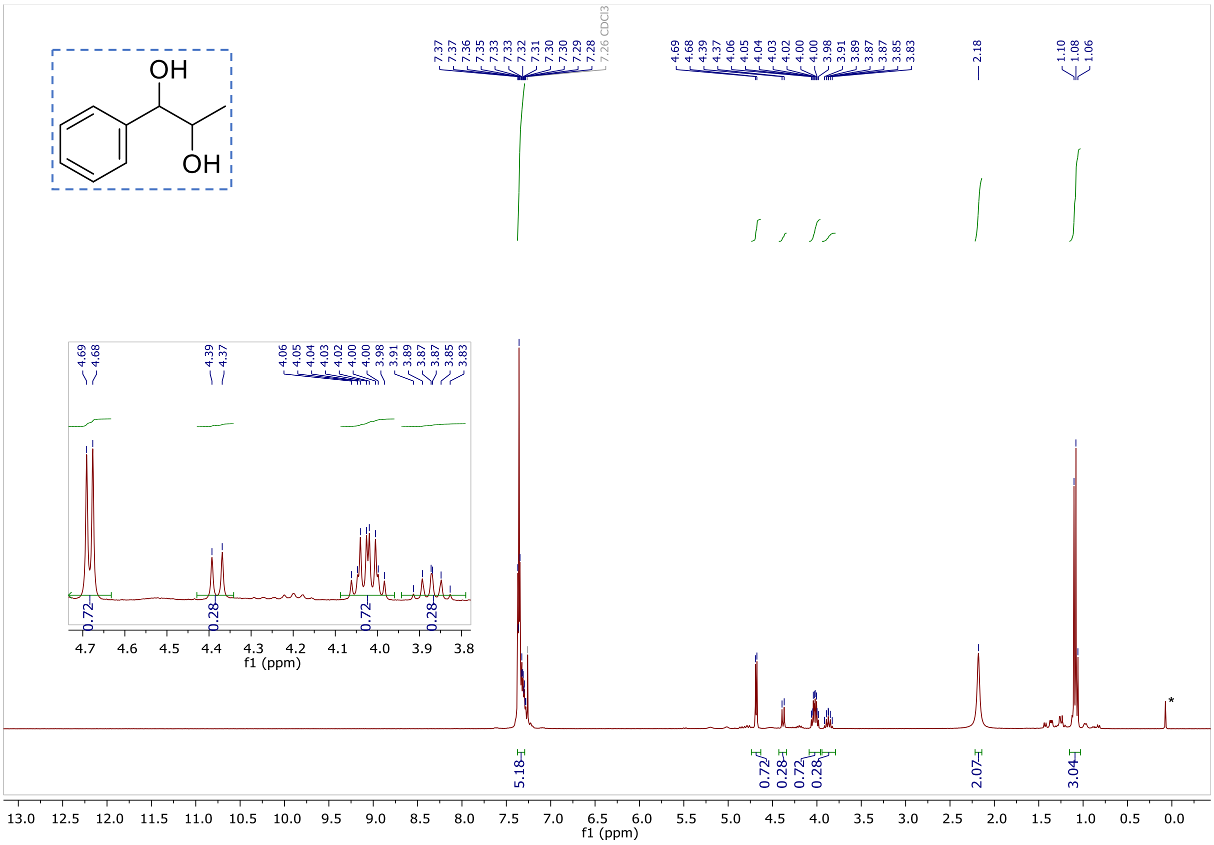

Figure S48 – ^{1}H NMR spectrum of **5o.**

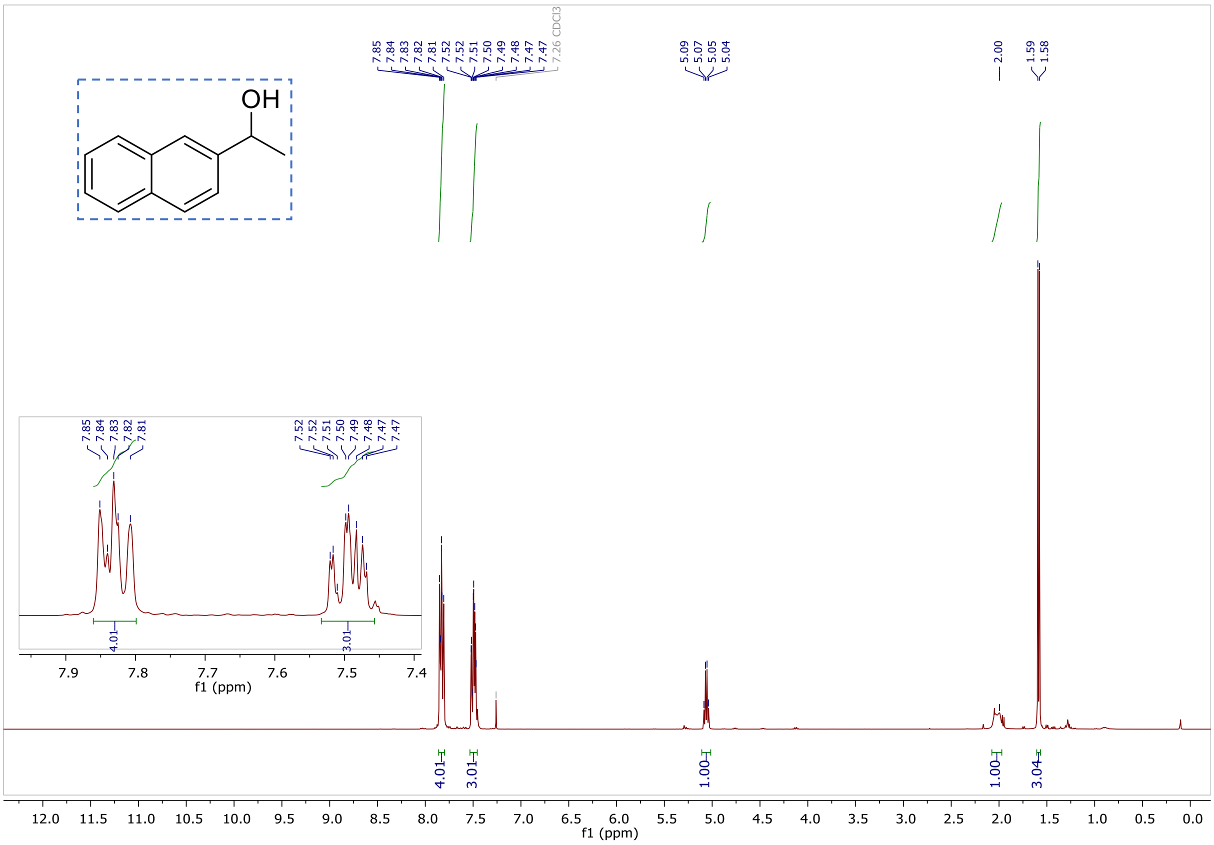

Figure S49 – ^{1}H NMR spectrum of **5p**.

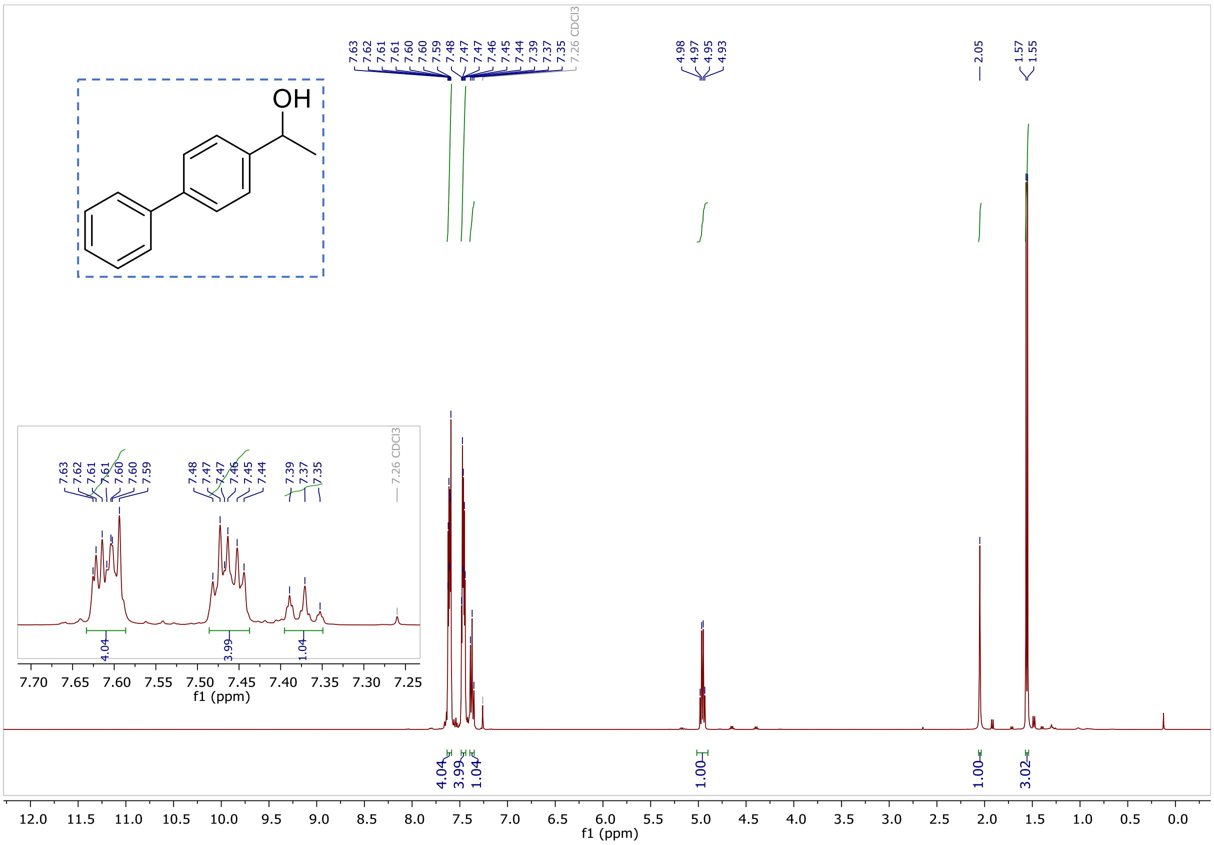

Figure S50 – ^{1}H NMR spectrum of **5q**.

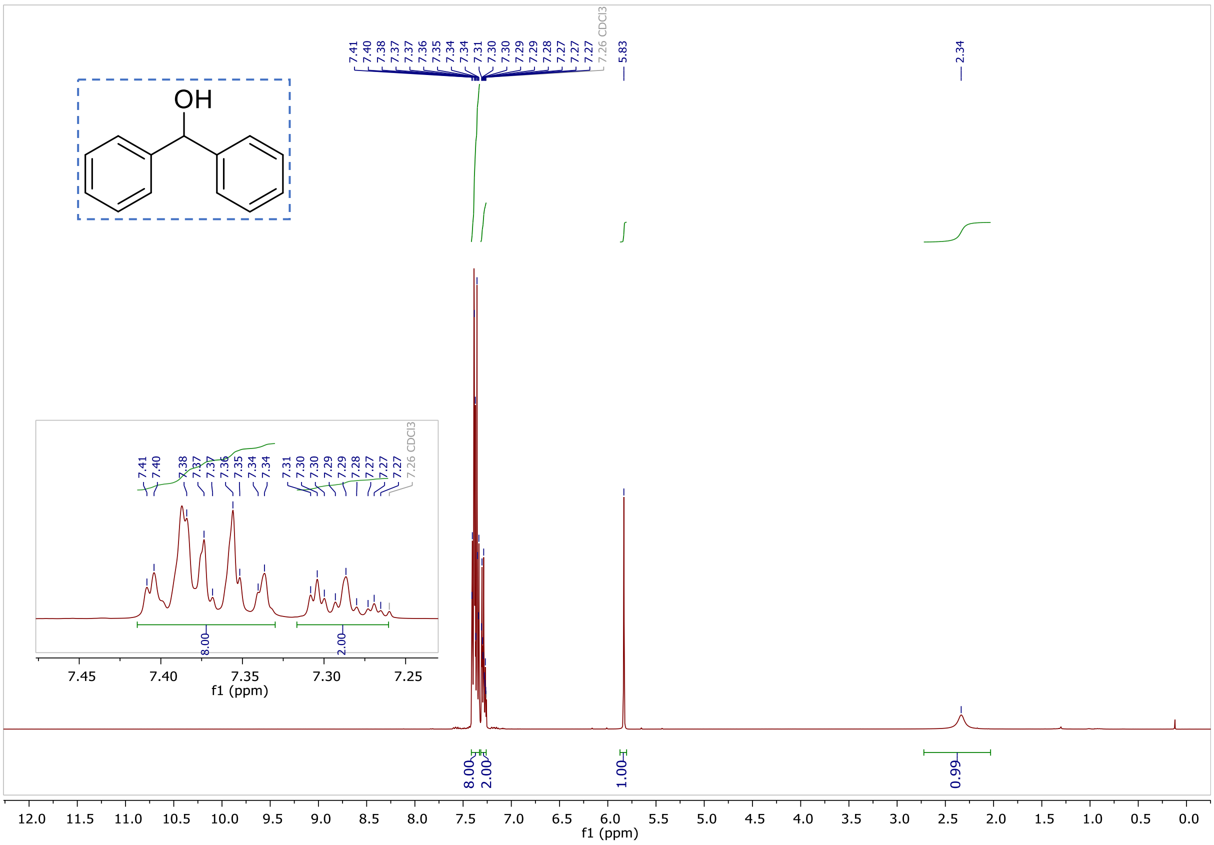

Figure S51 – ^{1}H NMR spectrum of **5r**.

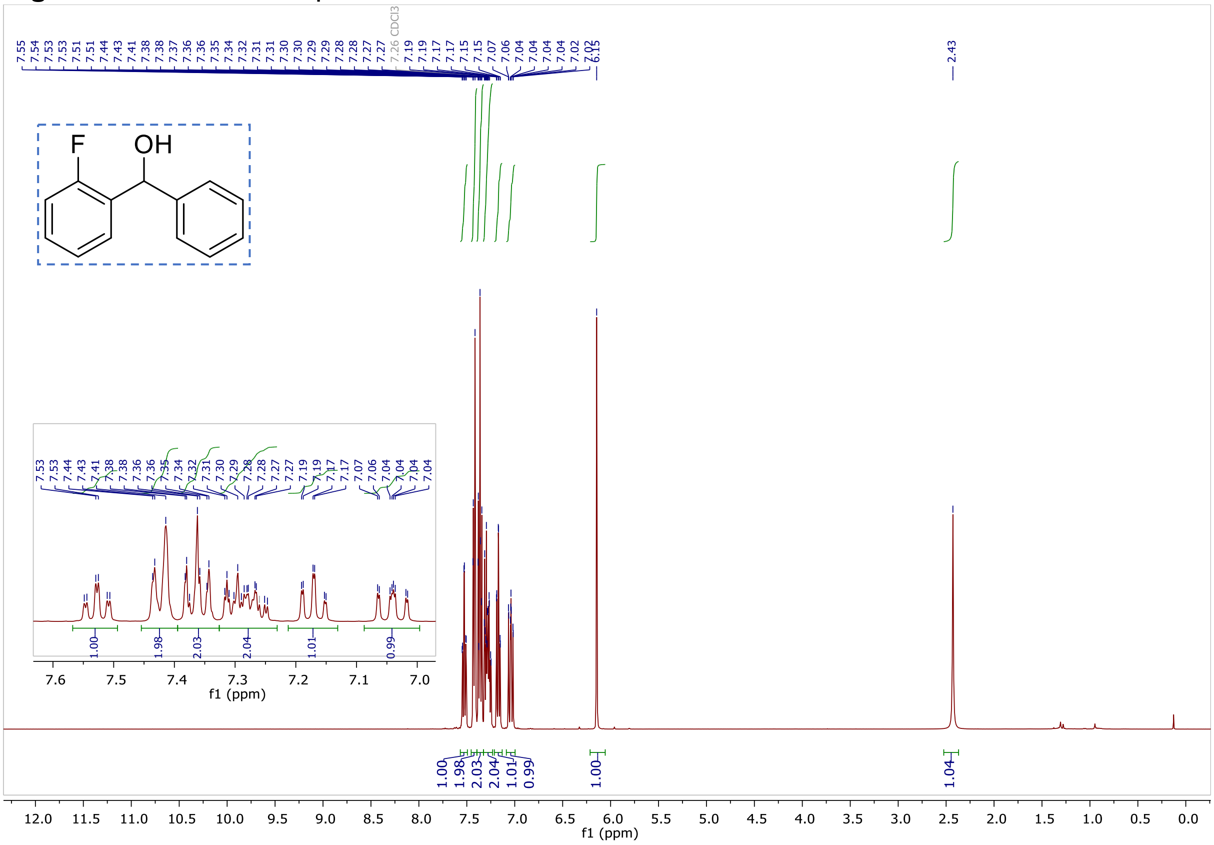

Figure S52 – ^{1}H NMR spectrum of **5s**.

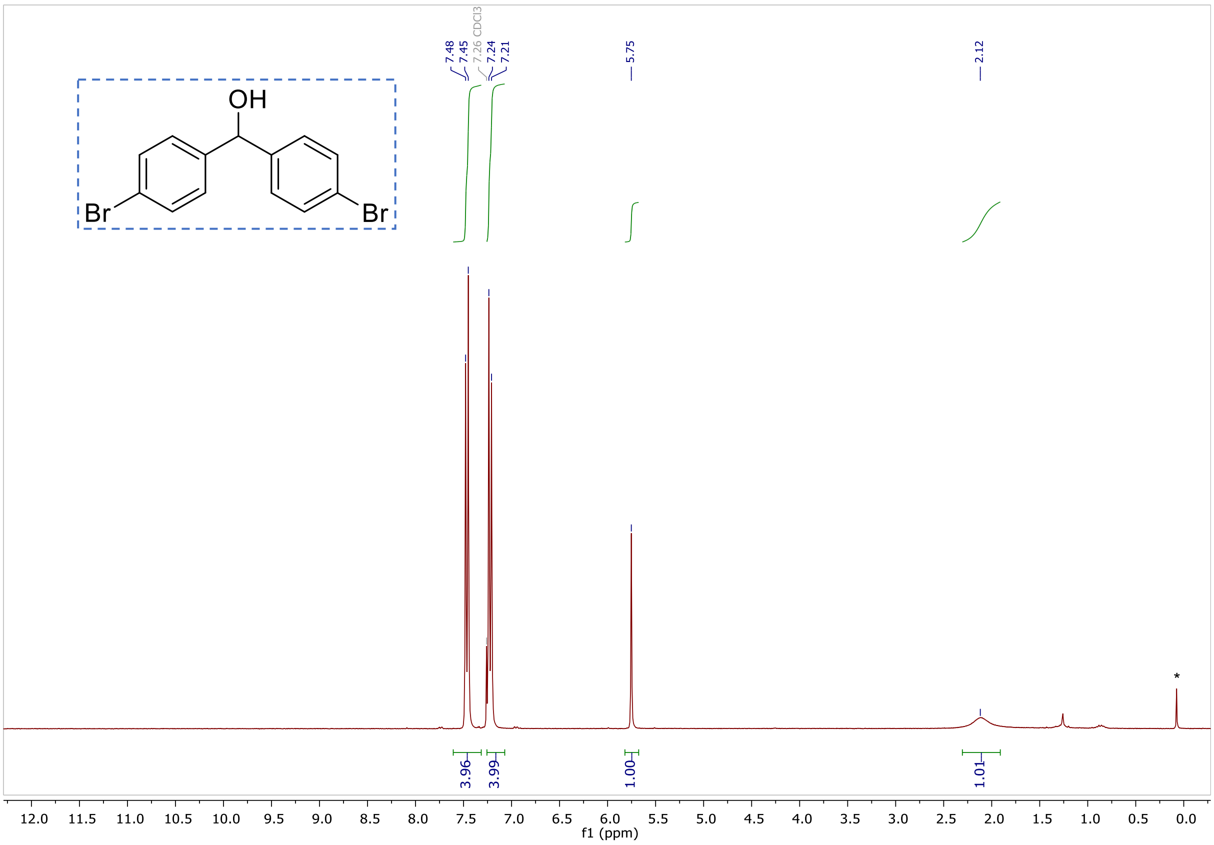

Figure S53 – ^{1}H NMR spectrum of **5t**. * - Grease.

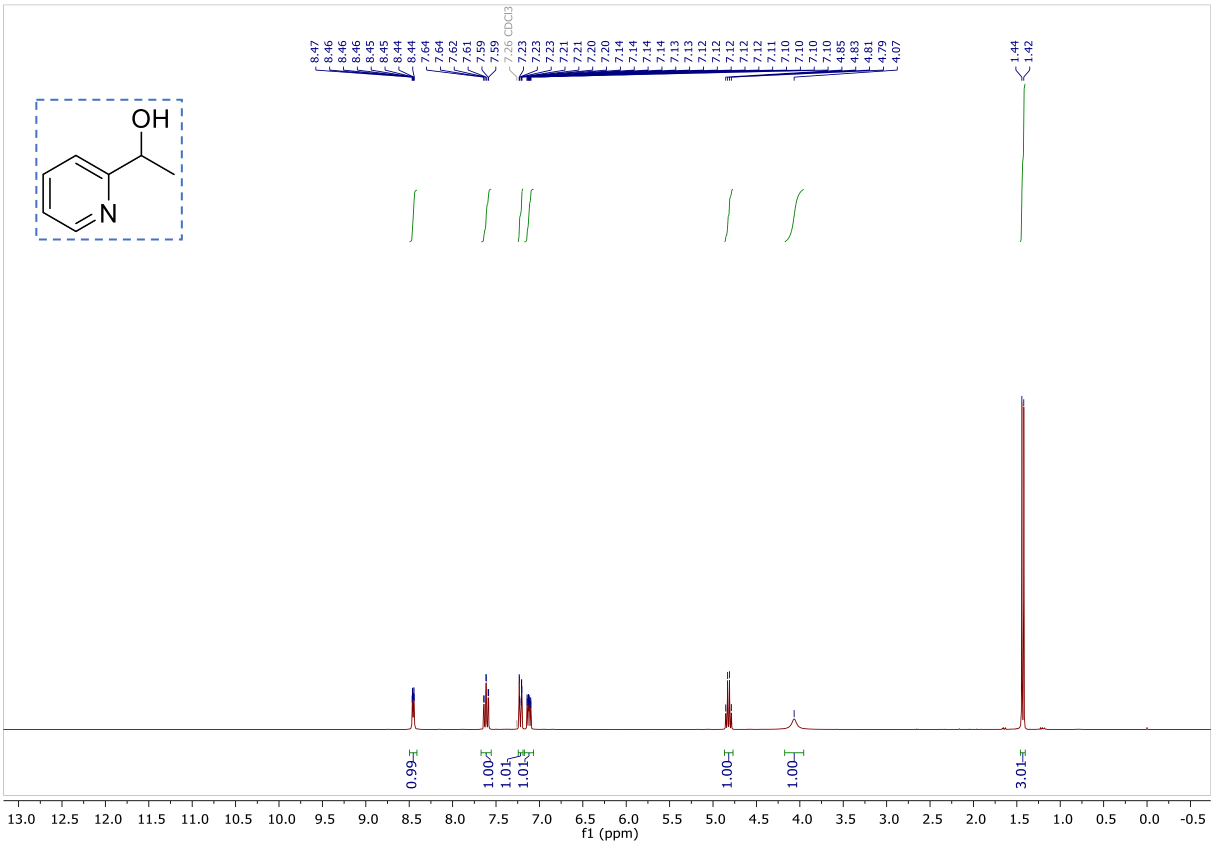

Figure S54 – ^{1}H NMR spectrum of **5u**.

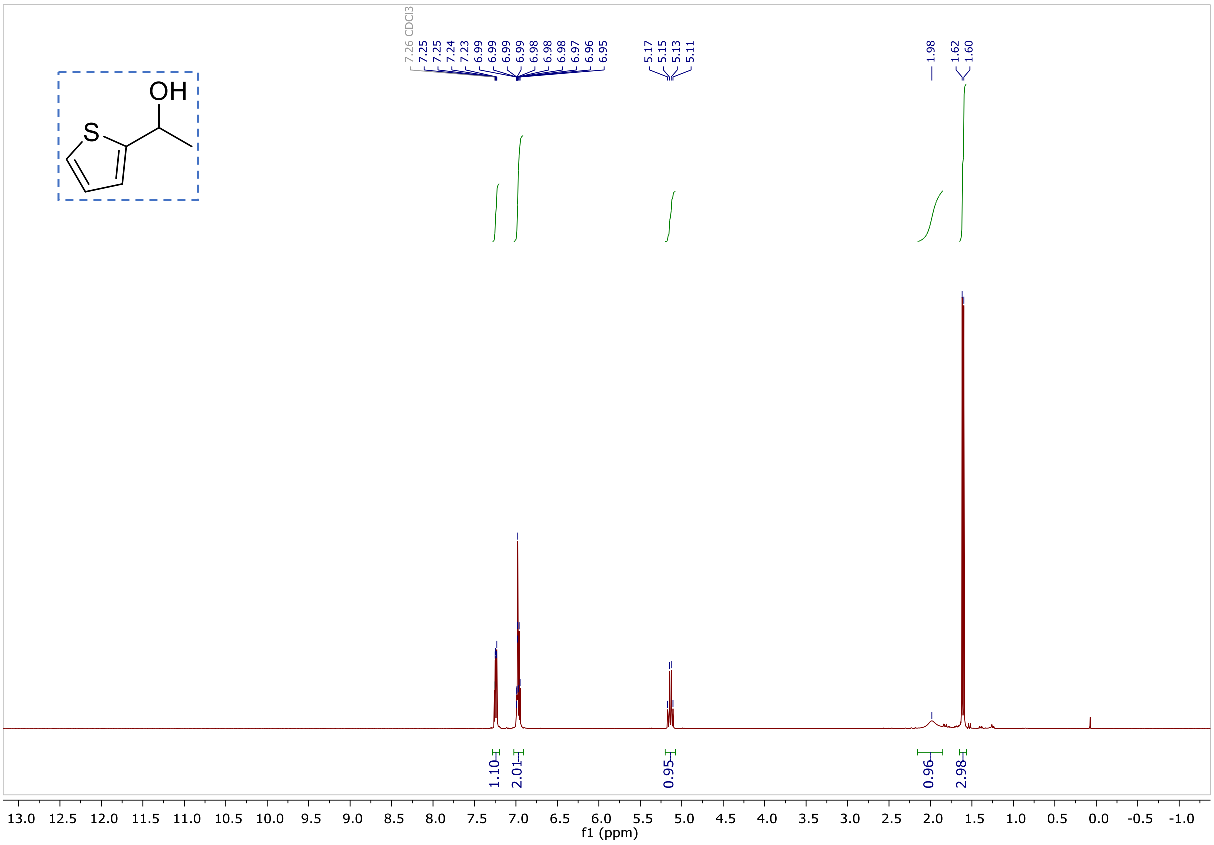

Figure S55 – ^{1}H NMR spectrum of **5v**.

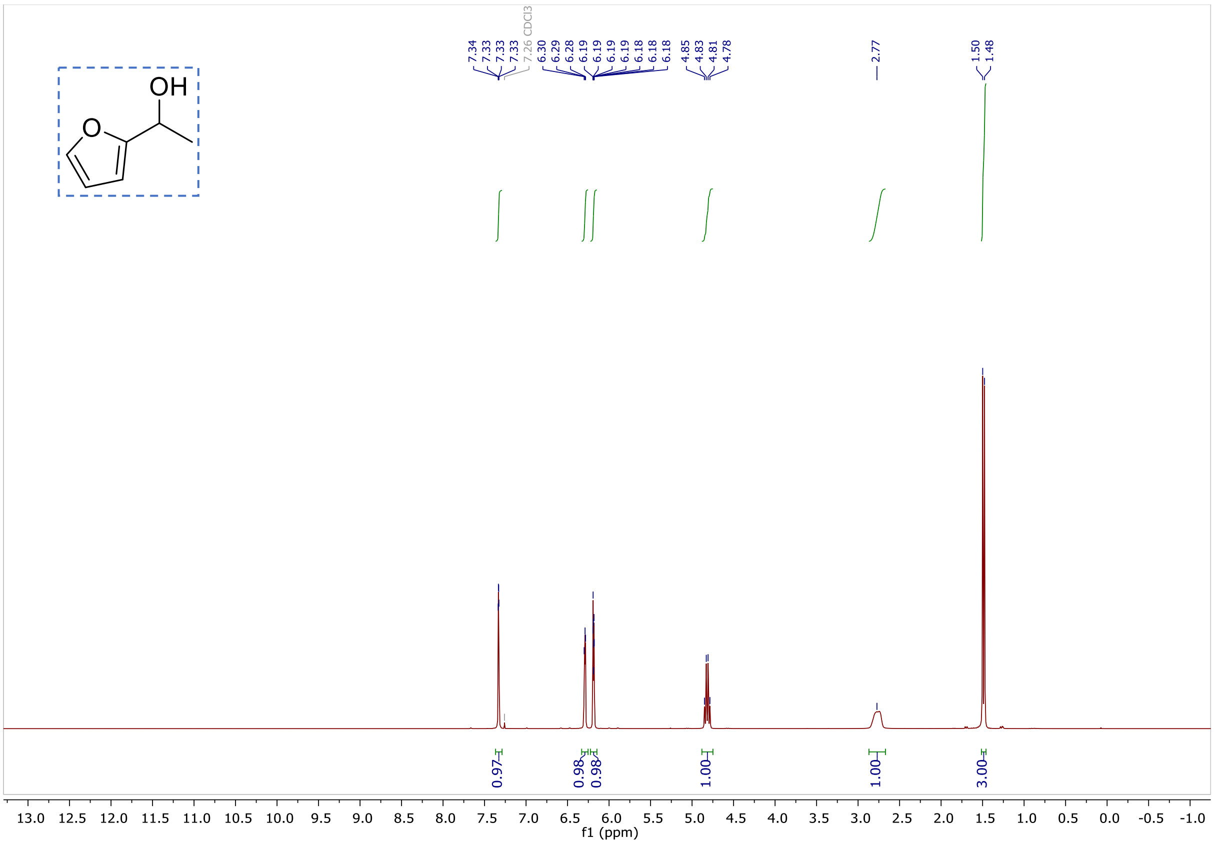

Figure S56 – ^{1}H NMR spectrum of **5w**.

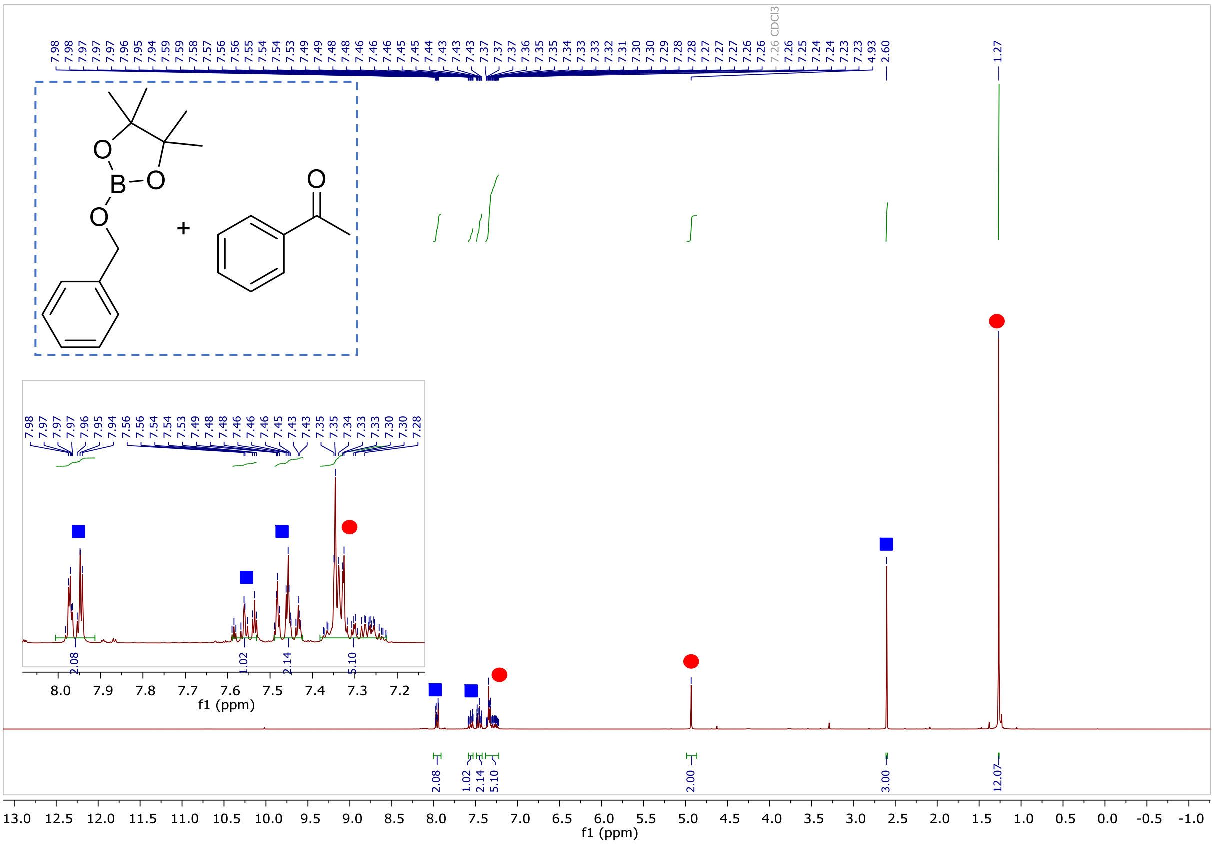

Figure S57 – ^{1}H NMR spectrum from the competitive hydroboration reaction of benzaldehyde and acetophenone (**1a**) with pinacolborane (**2**) as a reducing agent. Blue square (■) – signals from acetophenone; red circle (●) – signals from benzaldehyde hydroboration product.

6. Immobilization of $Pt(PPh_3)_4$ in [BMIM][NTf2]

50 mg (11.9 mmol) of [BMIM][NTf_2] was mixed with 10 mg (0.008 mmol) of $Pt(PPh_3)_4$. The mixture was stirred for 3 h at 60 °C under argon atmosphere. After this time, ^{1}H and ^{31}P NMR spectra were measured in $CDCl_3$.

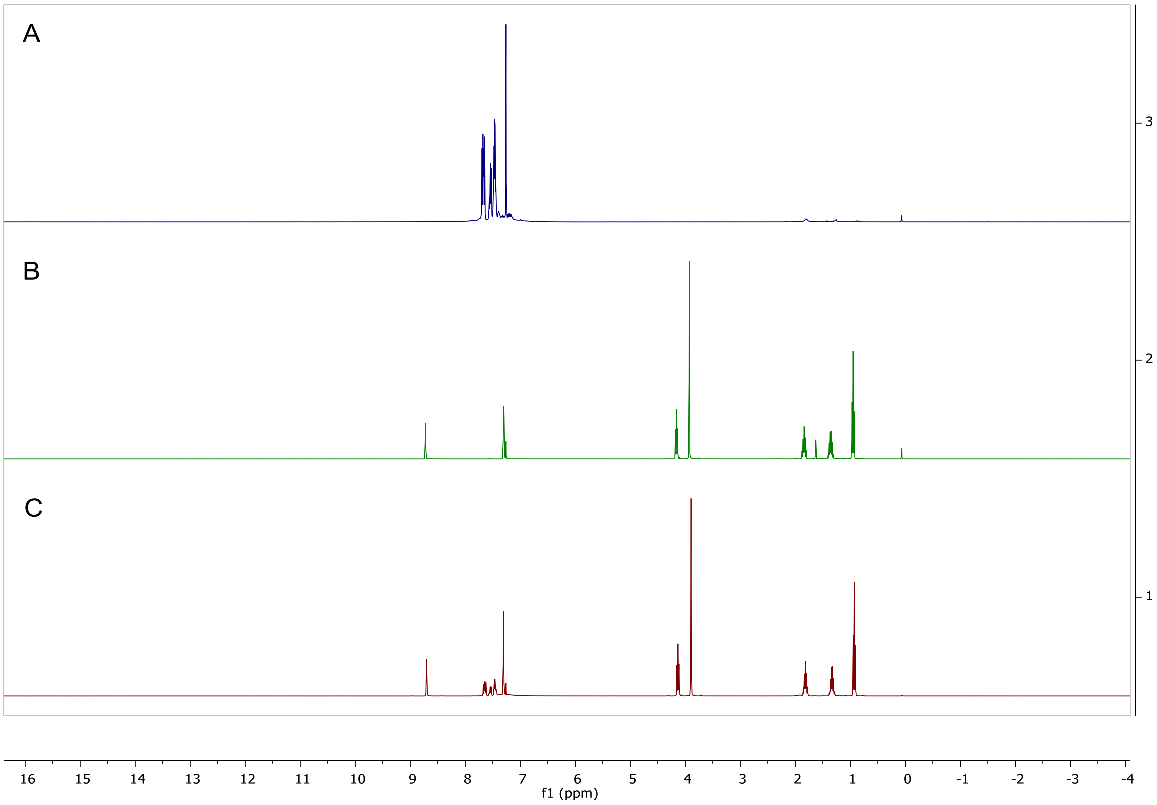

Figure S58 – ^{1}H NMR spectrum of $Pt(PPh_3)_4$ (A), [BMIM][NTf_2] (B) and mixture of $Pt(PPh_3)_4$ and BMIM][NTf_2] (C) .

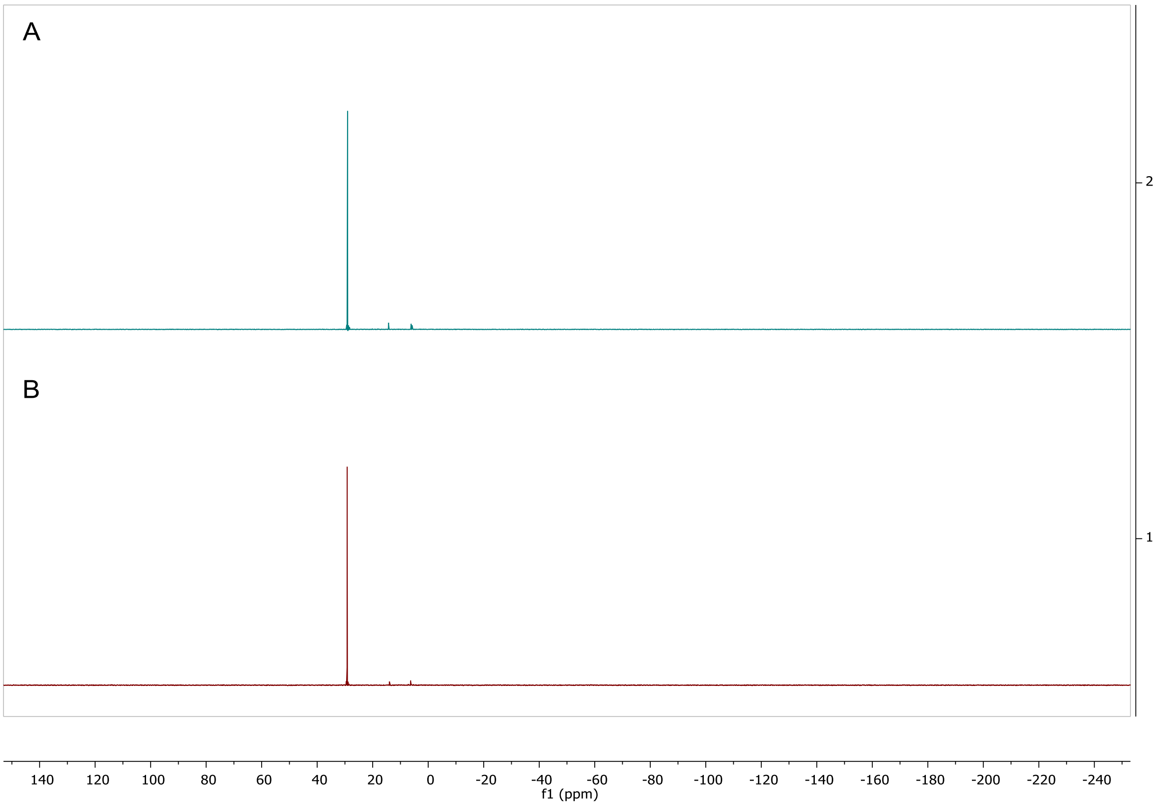

Figure S59 – ^{31}P NMR spectrum of $Pt(PPh_3)_4$ (A) and mixture of $Pt(PPh_3)_4$ and BMIM][NTf_2] (B).

7. Stability of HBpin in [BMIM][NTf$_2$]

To determine the stability of pinacolborane in the ionic liquid, pinacolborane (0.18 mmol, 32 mg) and [BMIM][NTf_2] (0.18 mmol, 75.5 mg) were mixed in equimolar ratio. The mixture was stirred for 3 h at 60 °C under argon atmosphere and then ^1H and ^{11}B NMR spectra were performed in $CDCl_3$. New signals or changes in the multiplicity of signals were not observed.

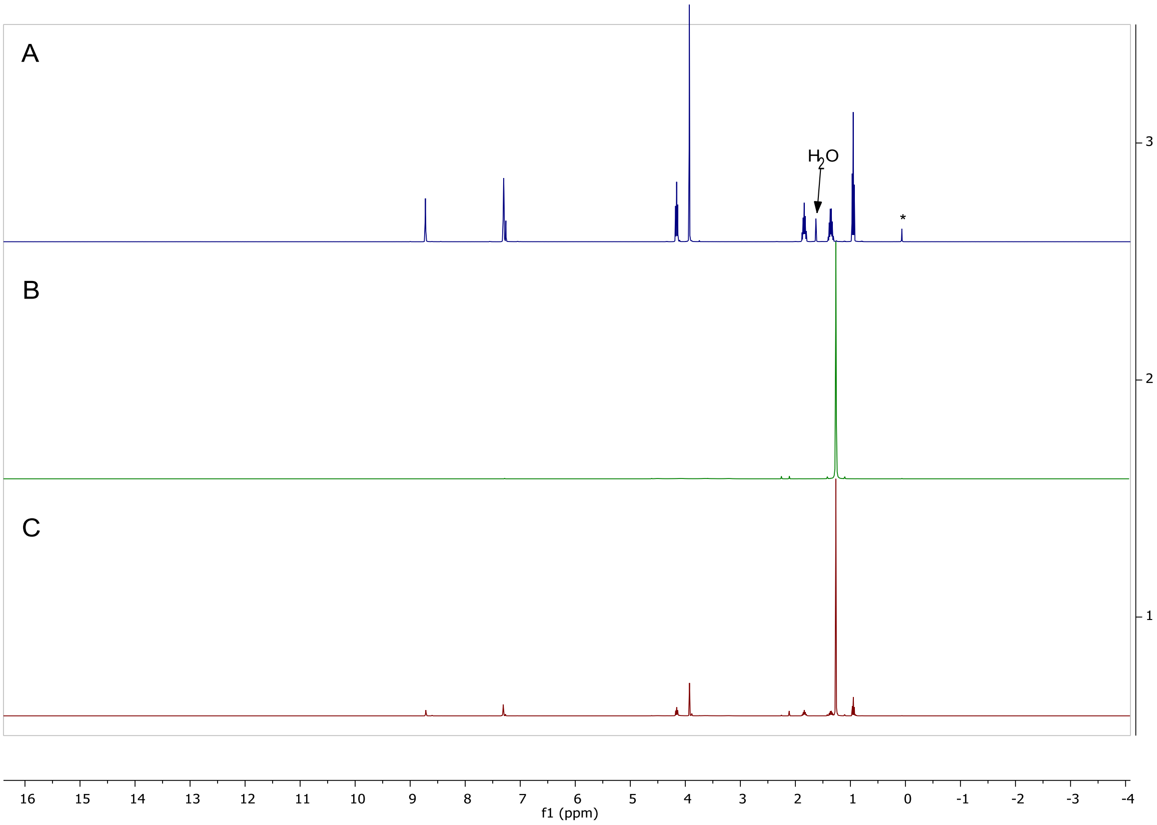

Figure S60 – ^{1}H NMR spectrum of [BMIM][NTf$_2$] (A), pinacolborane (B) and equimolar mixture of pinacolborane with [BMIM][NTf$_2$] (C). * - Grease.

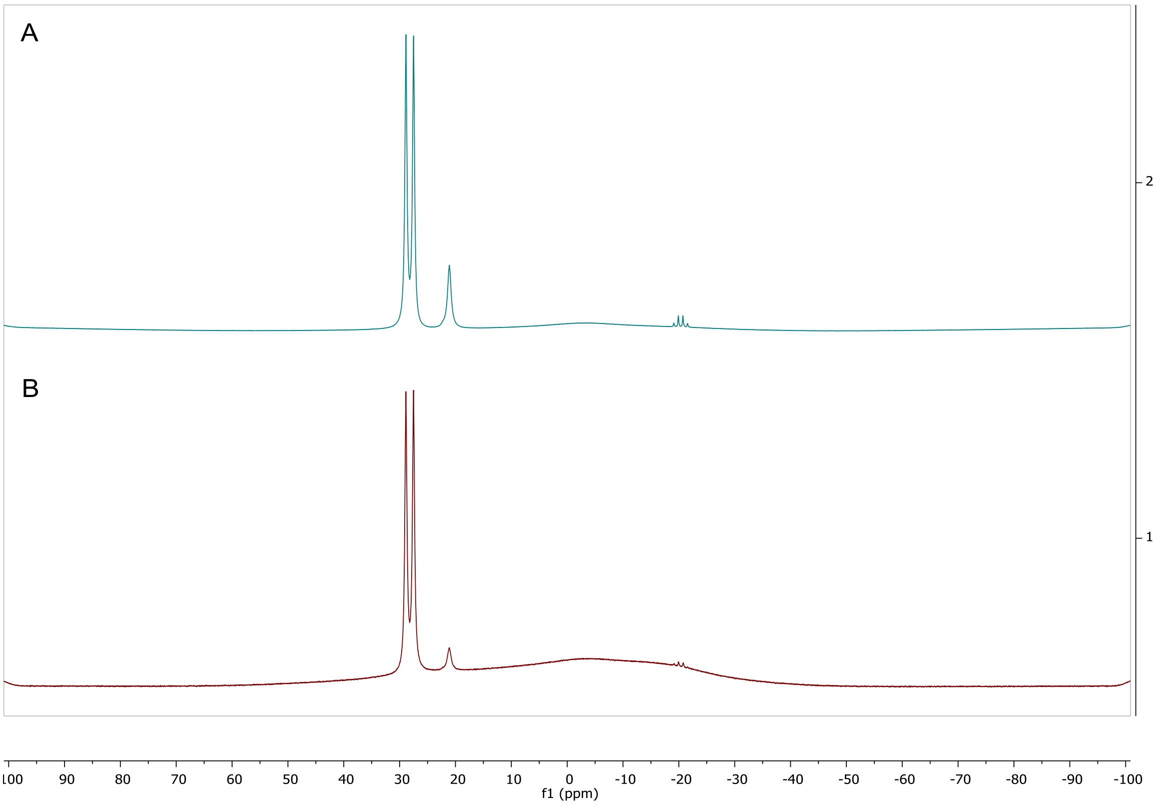

Figure S61 – ^{11}B NMR spectrum of pinacolborane (A) and an equimolar mixture of pinacolborane with [BMIM][NTf$_2$] (B)

8. Mechanistic studies

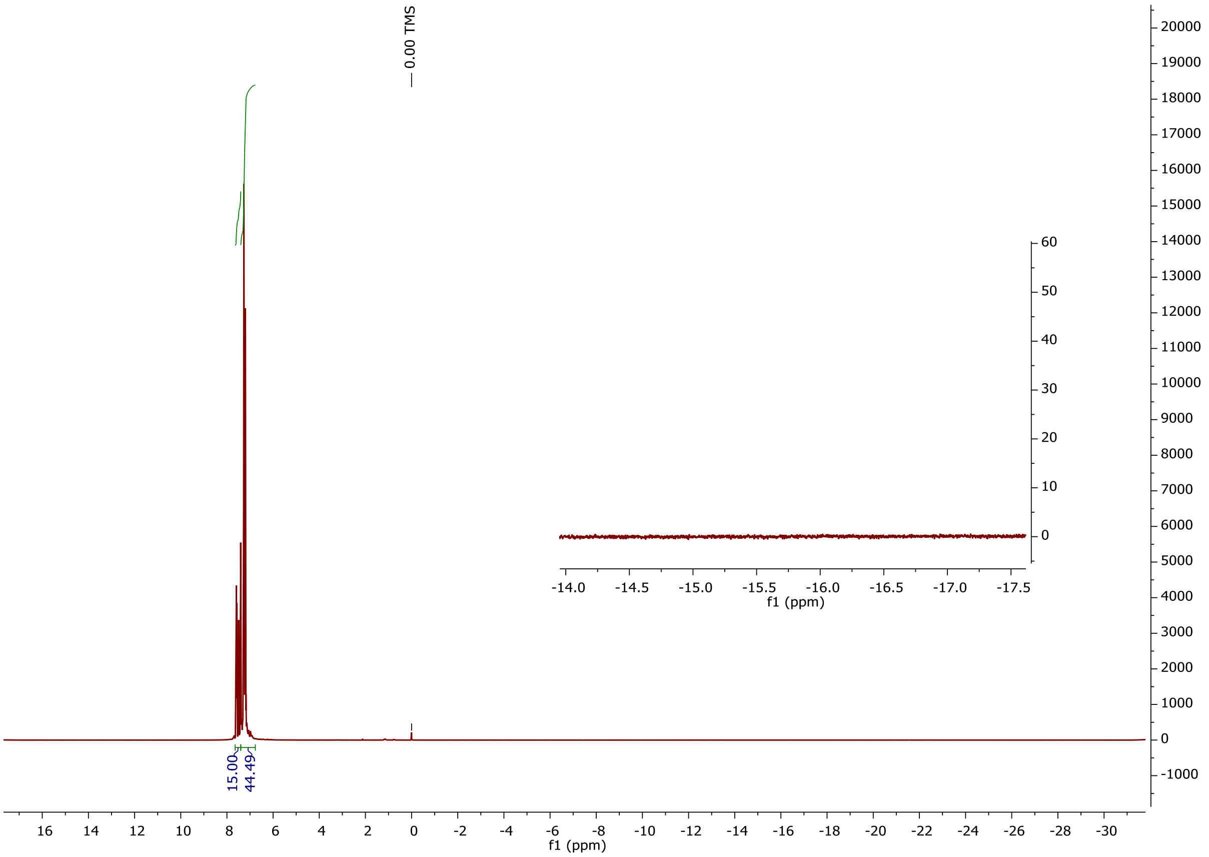

Figure S62 – Low-temperature ^1H NMR spectrum of $Pt(PPh_3)_4$.

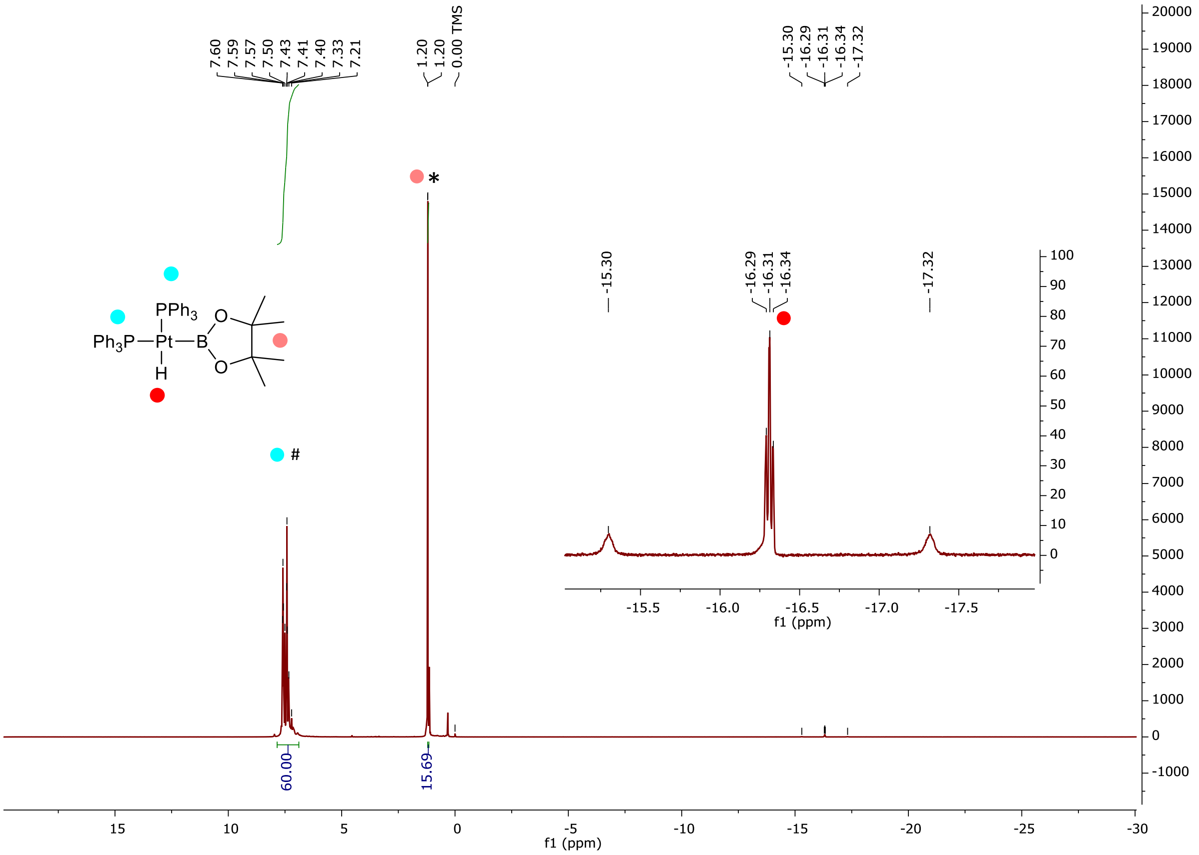

Figure S63 – Low-temperature ^{1}H NMR spectrum of the mixture of $Pt(PPh_3)_4$ (1 equiv.) and HBpin (1.2 equiv.). * Excess of HBpin. # Free PPh_3.

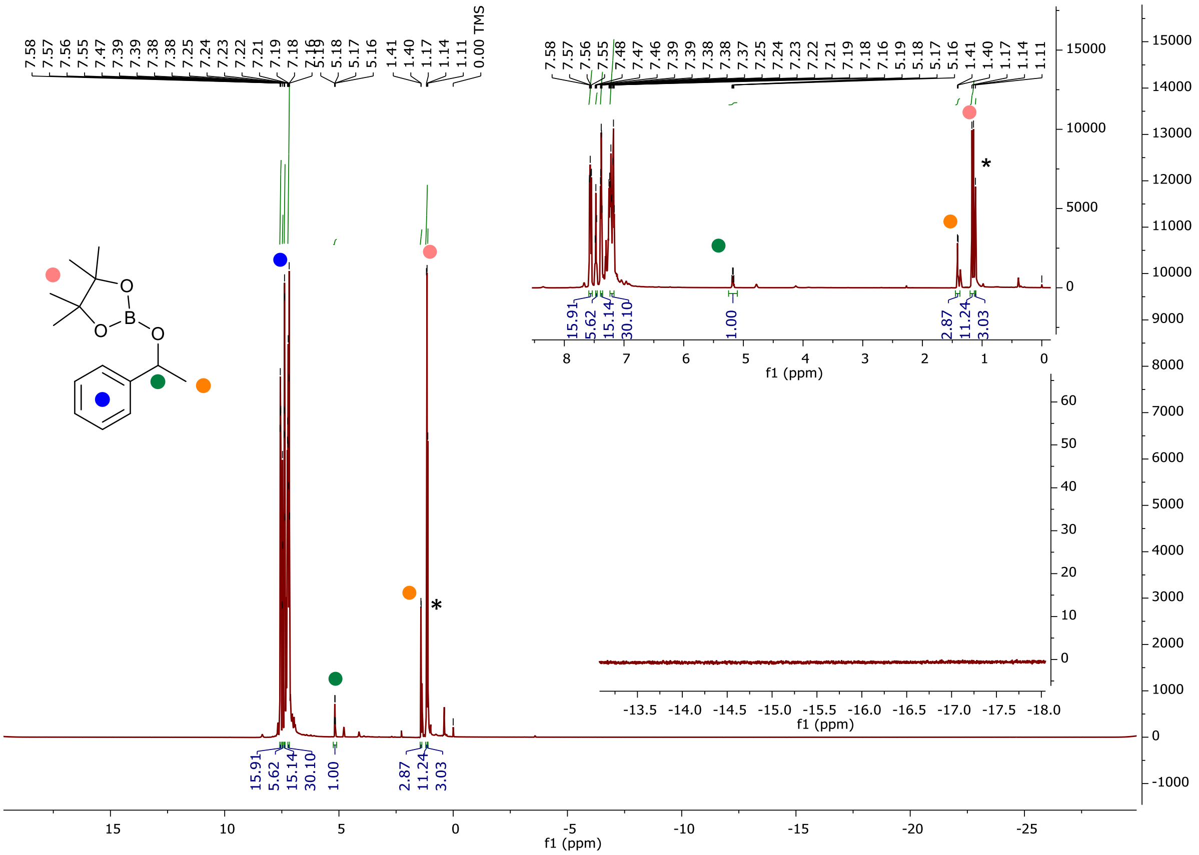

Figure S64 – Low-temperature ^{1}H NMR spectrum of the mixture of $Pt(PPh_3)_4$ (1 equiv.), HBpin (1.2 equiv.) and **1a** (1 eqiuv.) after 18 h at 55 °C. *Excess of HBpin.

9. References

1 Malatesta, L. & Cariello, C. 468. Platinum(0) compounds with triarylphosphines and analogous ligands. *J. Chem. Soc. (Resumed)*, 2323-2328 (1958).

2 Wang, W. *et al.* Catalyst-free and solvent-free hydroboration of ketones. *New J. Chem.* **43**, 10744-10749 (2019).

3 Zhang, G. *et al.* Highly selective hydroboration of alkenes, ketones and aldehydes catalyzed by a well-defined manganese complex. *Angew. Chem. Int. Ed.* **55**, 14369-14372 (2016).

4 Newland, R. J., Lynam, J. M. & Mansell, S. M. Small bite-angle 2-phosphinophosphinine ligands enable rhodium-catalysed hydroboration of carbonyls. *Chem. Commun.* **54**, 5482-5485 (2018).

5 Peddarao, T., Sarkar, N. & Nembenna, S. Mono- and bimetallic aluminum alkyl, alkoxide, halide and hydride complexes of a bulky conjugated bis-guanidinate(CBG) ligand and aluminum alkyls as precatalysts for carbonyl hydroboration. *Inorg. Chem.* **59**, 4693-4702 (2020).

6 DiBenedetto, T. A., Parsons, A. M. & Jones, W. D. Markovnikov-selective hydroboration of olefins catalyzed by a copper N-heterocyclic carbene complex. *Organometallics* **38**, 3322-3326 (2019).

7 Bole, L. J. *et al.* Progressing the frustrated lewis pair abilities of N-heterocyclic carbene/GaR_3 combinations for catalytic hydroboration of aldehydes and ketones. *Inorg. Chem.* **60**, 13784-13796 (2021).

8 Wang, W. *et al.* Grignard reagents-catalyzed hydroboration of aldehydes and ketones. *Tetrahedron* **76**, 131145 (2020).

9 Sarkar, N., Mahato, M. & Nembenna, S. Palladium-catalyzed selective reduction of carbonyl compounds. *Eur. J. Inorg. Chem.* **2020**, 2295-2301 (2020).

10 Mahato, S. *et al.* Hydroboration and reductive amination of ketones and aldehydes with HBpin by a bench stable Pd(II)-catalyst. *Org. Biomol. Chem.* **20**, 1103-1111 (2022).

11 Sarkar, N., Sahoo, R. K., Patro, A. G. & Nembenna, S. Aluminum-catalyzed selective hydroboration of carbonyls and dehydrocoupling of alcohols, phenols, amines, thiol, selenol, silanols with HBpin. *Polyhedron* **222**, 115902 (2022).

12 Romero, E. A., Peltier, J. L., Jazzar, R. & Bertrand, G. Catalyst-free dehydrocoupling of amines, alcohols, and thiols with pinacol borane and 9-borabicyclononane (9-BBN). *Chem.Commun.***52**, 10563-10565 (2016).

13 Jakhar, V. K., Barman, M. K. & Nembenna, S. Aluminum monohydride catalyzed selective hydroboration of carbonyl compounds. *Org. Lett.* **18**, 4710-4713 (2016).

14 Leong, B. X. *et al.* A versatile NHC-parent silyliumylidene cation for catalytic chemo- and regioselective hydroboration. *J. Am. Chem. Soc.* **141**, 17629-17636 (2019).

15 Li, T., Zhang, J. & Cui, C. Heterocyclic carbene-catalyzed hydride transfer in the hydroboration of carbonyl compounds. *Chin. J. Chem .* **37**, 679-683 (2019).

16 Willcox, D., Carden, J. L., Ruddy, A. J., Newman, P. D. & Melen, R. L. Asymmetric ketone hydroboration catalyzed by alkali metal complexes derived from BINOL ligands. *Dalton Trans.* **49**, 2417-2420 (2020).

17 Nagarjun, N. & Dhakshinamoorthy, A. A Cu-Doped ZIF-8 metal organic framework as a heterogeneous solid catalyst for aerobic oxidation of benzylic hydrocarbons. *New J. Chem.* **43**, 18702-18712 (2019).

18 Maier, T. M. *et al.* Low-valence anionic *alpha*-diimine iron complexes: synthesis, characterization, and catalytic hydroboration studies. *Inorg. Chem.* **59**, 16035-16052 (2020).

19 Estopiñá-Durán, S., McLean, E. B., Donnelly, L. J., Hockin, B. M. & Taylor, J. E. Arylboronic acid catalyzed C-alkylation and allylation reactions using benzylic alcohols. *Org. Lett.* **22**, 7547-7551 (2020).

20 Choudhary, N., Ghosh, T. & Mobin, S. M. Ketone hydrogenation by using ZnO−Cu(OH)Cl/MCM-41 with a splash of water: an environmentally benign approach. *Chem. - Asian J.* **15**, 1339-1348 (2020).

21 Ramachandran, P. V., Alawaed, A. A. & Hamann, H. J. $TiCl_4$-catalyzed hydroboration of ketones with ammonia borane. *J. Org. Chem.* **87**, 13259-13269 (2022).
22 Vilches-Herrera, M. *et al.* Reduction over condensation of carbonyl compounds through a transient hemiaminal intermediate using hydrazine. *J. Org. Chem.* **85**, 9213-9218 (2020).
23 Liu, J.-t., Yang, S., Tang, W., Yang, Z. & Xu, J. Iridium-catalyzed efficient reduction of ketones in water with formic acid as a hydride donor at low catalyst loading. *Green Chem.* **20**, 2118-2124 (2018).
24 Puls, F., Linke, P., Kataeva, O. & Knölker, H.-J. Iron-catalyzed wacker-type oxidation of olefins at room temperature with 1,3-diketones or neocuproine as ligands. *Angew. Chem. Int. Ed.* **60**, 14083-14090 (2021).
25 Liu, J., Li, W., Li, Y., Liu, Y. & Ke, Z. Selective C-alkylation between alcohols catalyzed by N-heterocyclic carbene molybdenum. *Chem. - Asian J.* **16**, 3124-3128 (2021).
26 Wang, S., Huang, H., Tsareva, S., Bruneau, C. & Fischmeister, C. Silver-catalyzed hydrogenation of ketones under mild conditions. *Adv. Synth. Catal.* **361**, 786-790 (2019).
27 Li, G.-X. *et al.* A unified photoredox-catalysis strategy for C(sp^3)–H hydroxylation and amidation using hypervalent iodine. *Chem. Sci.* **8**, 7180-7185 (2017).
28 Fujieda, N. *et al.* A well-defined osmium–cupin complex: hyperstable artificial osmium peroxygenase. *J. Am. Chem. Soc.* **139**, 5149-5155 (2017).
29 Liu, M. *et al.* Stepwise degradation of hydroxyl compounds to aldehydes via successive C–C bond cleavage. *Chem. Commun.* **55**, 925-928 (2019).
30 Pelter, A., Buss, D., Colclough, E. & Singaram, B. Hindered organoboron groups in organic chemistry. 23. The interactions of dimesitylboron stabilised carbanions with aromatic ketones and aldehydes to give alkenes. *Tetrahedron* **49**, 7077-7103 (1993).
31 Falconnet, A., Magre, M., Maity, B., Cavallo, L. & Rueping, M. Asymmetric magnesium-catalyzed hydroboration by metal-ligand cooperative catalysis. *Angew. Chem. Int. Ed.* **58**, 17567-17571 (2019).
32 Shinohara, K., Tsurugi, H., Anwander, R. & Mashima, K. Trivalent rare-earth metal amide complexes as catalysts for the hydrosilylation of benzophenone derivatives with $HN(SiHMe_2)_2$ by amine-exchange reaction. *Chem. Eur. J.* **26**, 14130-14136 (2020).
33 Junquera, L. B., Fernández, F. E., Puerta, M. C. & Valerga, P. Nickel(II) N-Heterocyclic carbene complexes: versatile catalysts for C–C, C–S and C–N coupling reactions. *Eur. J. Inorg. Chem.* **2017**, 2547-2556 (2017).
34 Zou, T., Pi, S.-S. & Li, J.-H. $FeCl_3$-catalyzed 1,2-addition reactions of aryl aldehydes with arylboronic acids. *Org. Lett.* **11**, 453-456 (2009).
35 Manikandan, T. S., Saranya, S. & Ramesh, R. Synthesis and catalytic evaluation of ruthenium(II) benzhydrazone complex in transfer hydrogenation of ketones. *Tetrahedron Lett.* **57**, 3764-3769 (2016).
36 Hsu, S.-F. & Plietker, B. Selective transfer hydrogenation and hydrogenation of ketones using a defined monofunctional (P^N(Bn)^N(Bn)^P)–Ru^{II} complex. *Chem. Eur. J.* **20**, 4242-4245 (2014).
